# An improved approximate-Bayesian model-choice method for estimating shared evolutionary history

**DOI:** 10.1186/1471-2148-14-150

**Published:** 2014-07-03

**Authors:** Jamie R Oaks

**Affiliations:** 1 Department of Ecology and Evolutionary Biology, University of Kansas, 1200 Sunnyside Avenue, Lawrence Kansas 66045, USA; 2 Department of Biology, University of Washington, Box 351800, Seattle, Washington 98195, USA

**Keywords:** Dirichlet-process prior, Approximate-Bayesian computation, Model choice, Phylogeography, Biogeography

## Abstract

**Background:**

To understand biological diversification, it is important to account for large-scale processes that affect the evolutionary history of groups of co-distributed populations of organisms. Such events predict temporally clustered divergences times, a pattern that can be estimated using genetic data from co-distributed species. I introduce a new approximate-Bayesian method for comparative phylogeographical model-choice that estimates the temporal distribution of divergences across taxa from multi-locus DNA sequence data. The model is an extension of that implemented in msBayes.

**Results:**

By reparameterizing the model, introducing more flexible priors on demographic and divergence-time parameters, and implementing a non-parametric Dirichlet-process prior over divergence models, I improved the robustness, accuracy, and power of the method for estimating shared evolutionary history across taxa.

**Conclusions:**

The results demonstrate the improved performance of the new method is due to (1) more appropriate priors on divergence-time and demographic parameters that avoid prohibitively small marginal likelihoods for models with more divergence events, and (2) the Dirichlet-process providing a flexible prior on divergence histories that does not strongly disfavor models with intermediate numbers of divergence events. The new method yields more robust estimates of posterior uncertainty, and thus greatly reduces the tendency to incorrectly estimate models of shared evolutionary history with strong support.

## Background

Understanding the processes that generate biodiversity and regulate community assembly is a major goal of evolutionary biology. Large-scale changes to the environment, including geological and climatic events, can affect the evolutionary history of entire communities of co-distributed species and their associated microbiota. For example, by partitioning communities, such an event can isolate groups of populations and cause a temporal cluster of speciation events across co-distributed taxa. Given the dynamic nature of our planet, such biogeographical processes likely play a significant role in determining diversification rates and patterns. At recent timescales, temporal clusters of diversification caused by biogeographical events can leave a signature in the genetic variation within and among the affected lineages. Thus, methods for accurately estimating models of shared evolutionary events across co-distributed taxa from genetic data are important for better understanding how regional and global biogeographical processes affect biodiversity.

This inference problem is challenging due to the stochastic nature by which mutations occur in populations and how they are inherited over generations [1,2]. Thus, a method for estimating historical patterns of divergences across taxa should explicitly model the stochastic mutational and ancestral processes that generate and filter the genetic variation we observe in present-day genetic data. An appealing approach would be a comparative, Bayesian model-choice method for inferring the probability of competing divergence histories while integrating over uncertainty in mutational and ancestral processes via models of nucleotide substitution and lineage coalescence. The sample space of such a model-choice procedure would include all models ranging from a single divergence-time parameter (i.e., simultaneous divergence of all co-distributed taxa) to the fully generalized model in which each taxon diverged at a unique time.

The software package msBayes implements such an approach in an approximate-Bayesian model-choice framework [3,4]. The method models temporally clustered divergences across taxa caused by a biogeographical event (or a “divergence event”) as a single, instantaneous occurrence. In other words, a divergence event causes a set of taxa to share the same moment of divergence along a continuous time scale (i.e., simultaneous divergence). Given aligned sequence data for *Y* pairs of populations, msBayes estimates the number of divergence events shared among the pairs, the timing of the events, and the assignment of pairs to the events, while integrating out uncertainty in demographic parameters and the genealogical histories of the sequences. Thus, the method samples over all possible divergence models of differing dimensionality (i.e., all the possible partitions of *Y* pairs to 1,2,…,*Y* divergence-time parameters), and, in so doing, estimates the posterior probability of each model.

msBayes has been used to address biogeographical questions in a variety of empirical systems. Some examples include (1) whether the rise of the Isthmus of Panama caused co-divergence among species of echinoids co-distributed across the Pacific and Atlantic sides of the isthmus [3], (2) if an historical seaway across the Baja Peninsula caused co-divergence across species of squamates and mammals co-distributed both north and south of the putative seaway [5], (3) if species of gall-wasps and their associated parasitoids share divergences across putative glacial refugia [6], and (4) whether repeated fragmentation of the oceanic Islands of the Philippines during Pleistocene sea-level fluctuations caused diversification of vertebrate taxa distributed across the islands [7]. Such applications of the method often result in strong posterior support for co-divergence among all or subsets of the taxa investigated (e.g., [3,5-12]).

For priors on divergence-time and demographic parameters, msBayes uses continuous uniform probability distributions. This causes divergence models with more divergence-time parameters to integrate over a *much* greater parameter space with low likelihood yet high prior density, which can result in small marginal likelihoods relative to models with fewer divergence-time parameters [13,14]. Given that the marginal likelihood of a model weighted by its prior is what determines its posterior probability, this can cause support for models with fewer divergence events [7,15]. This is not a critique of Bayesian model choice in general; comparing models by their marginal likelihoods provides a “natural” penalty for over-parameterization and can be a great strength of the Bayesian approach. However, given the sensitivity of marginal likelihoods to the prior, care is needed when selecting prior distributions [14]. Selecting distributions that will often place high prior density in large regions of parameter space with low likelihood can lead to small marginal likelihoods of parameter-rich models even if they are correct.

Furthermore, msBayes uses a discrete uniform prior over the number of divergence events 1,2,…,*Y*. Because there are many more possible assignments of population pairs to intermediate numbers of divergence events, this imposes a prior on divergence models that puts most of the prior mass on models with either very few or very many divergence-time parameters (see Figure five of [7]; for brevity I will refer to this prior as “U-shaped”). Given that models with many divergence events can have small marginal likelihoods due to the uniform priors on divergence-time parameters, the U-shaped prior will effectively create a strong prior preference for models with very few divergence events.

Recently, Oaks et al. [7,15] found via simulation that msBayes will often strongly support models with a small number of divergence events shared among taxa, even when divergences were random over broad timescales. They suggested this behavior was due to the combination of uniform priors on parameters causing small marginal likelihoods of richer models and the U-shaped prior on divergence models. Hickerson et al. [16] suggested the problem was caused by sampling error, and proposed as a solution an approximate-Bayesian model averaging approach that samples over empirically informed uniform priors. However, Oaks et al. [15] evaluated the approach proposed by Hickerson et al. [16] using simulations and found that it did not mitigate the method’s propensity to incorrectly infer clustered divergences, and often preferred priors that excluded the true values of the model’s parameters. Here, I describe a new approach that successfully mitigates spurious inference of co-divergence while avoiding negative side effects of empirically informed uniform priors.

In this study, I introduce a new method, implemented in the software dpp-msbayes, that extends the model of msBayes. I use this method to test whether alternative parameterizations and priors improve the behavior of the approximate-Bayesian model-choice approach to estimating shared divergence events. The new approach uses a Dirichlet-process prior (DPP) over all possible models of divergence, and gamma and beta probability distributions in place of uniform priors on many of the model’s parameters. Using simulations, I show that the new implementation has improved robustness, accuracy, and power compared to the original model. The results confirm that the improved performance of the new model is due to a combination of (1) more flexible priors on divergence-time and demographic parameters that avoid placing high prior density in improbable regions of parameter space, and (2) a diffuse Dirichlet-process prior that does not strongly disfavor divergence models with intermediate numbers of divergence events. After reanalyzing sequence data from 22 pairs of taxa from the Philippines [7] under the new model, I find a large amount of posterior uncertainty in the number of divergence events shared among the taxa; a result in contrast with the original msBayes model and congruent with intuition given the richness of the model and the relatively small amount of information in the data.

## Methods

### The model

In this section, I describe the model, which is a modification of the model implemented in msBayes[4,7]. The code implementing the new model is freely available in the open-source software package dpp-msBayes (https://github.com/joaks1/dpp-msbayes). To perform the analyses described below, I used the freely avaliable, open-source software package PyMsBayes (https://github.com/joaks1/PyMsBayes), which provides a multi-processing interface to msBayes and dpp-msBayes. I performed the work described below following the principles of Open Notebook Science. Using version-control software, I make progress in all aspects of the work freely and publicly available in real-time at https://github.com/joaks1/msbayes-experiments. All information necessary to reproduce my results is provided there. I follow much of the notation of Oaks et al. [7], but modify it to aid in the description of the new model. A summary of my notation can be found in Table 1.

**Table 1:** **Summary of the notation used throughout this work; modified from Oaks et al. **[7]

**Symbol**	**Description**
*Y*	Number of population pairs.
*n*_*i*_	The number of genome copies sampled from population pair *i*, with *n*_1,*i*_ sampled from population 1, and *n*_2,*i*_ from population 2.
*k*_*i*_	Number of loci sampled from population pair *i*.
*K*	Total number of unique loci sampled.
*X*_*i*, *j*_	Sequence alignment of locus *j* sampled from population pair *i*.
$S_{i,\;j}^{*}$	Population genetic summary statistics calculated from *X*_*i*, *j*_.
**X**	Vector containing the sequence alignments of each locus from each population pair: $\left(X_{1,1},\ldots ,X_{Y}{,}_{k_{Y}}\right)$.
**S**^∗^	Vector containing the summary statistics of each locus from each population pair: $\left({\text{S}}_{1,1}^{*},\ldots ,{\text{S}}_{Y,k_{Y}}^{*}\right)$.
*B*_*ε*_(**S**^∗^)	Multi-dimensional Euclidean space around the observed summary statistics, **S**^∗^.
*ε*	Radius of *B*_*ε*_(**S**^∗^), i.e., the tolerance of the ABC estimation.
*G*_*i*, *j*_	Gene tree of the sequences in *X*_*i*, *j*_.
**G**	Vector containing the gene trees of each locus from each population pair: $\left(G_{1,1},\ldots ,G_{Y,k_{Y}}\right)$.
|***τ***|	Number of population divergence-time parameters shared among the *Y* population pairs.
*τ*	Time of population divergence in 4*N*_*C*_ generations.
***τ***	Set of divergence-time parameters: {*τ*_1_,…,*τ*_|***τ***|_}.
*t*_*i*_	The index of the divergence-time in ***τ*** to which population pair *i* is mapped.
**t**	Vector of divergence-time indices: (*t*_1_,…,*t*_*Y*_).
*T*_*i*_	Time of divergence in 4*N*_*C*_ generations between the populations of pair *i*.
**T**	Vector of divergence times for each of the population pairs: (*T*_1_,…,*T*_*Y*_).
$T_{i,\;j}$	Scaled time of divergence between the populations of pair *i* for locus *j*.
	Vector containing the scaled divergence times of each locus from each population pair: $\left(T_{1,1},\ldots ,T_{Y,k_{Y}}\right)$.
*θ*_*D*1,*i*_,*θ*_*D*2,*i*_	Mutation-rate-scaled effective population size of the 1^*st*^ and 2^*nd*^ descendent population, respectively, of pair *i*.
*θ*_*A*,*i*_	Mutation-rate-scaled effective population size of the population ancestral to pair *i*.
***θ***_***D1***_,***θ***_***D2***_	Vectors (*θ*_*D*1,1_,…,*θ*_*D*1,*Y*_) and (*θ*_*D*2,1_,…,*θ*_*D*2,*Y*_), respectively.
***θ***_***A***_	Vector containing the *θ*_*A*_ parameters for each population pair: (*θ*_*A*,1_,…,*θ*_*A*,*Y*_).
*υ*_*j*_	Mutation-rate multiplier of locus *j*.
***υ***	Vector containing the locus-specific mutation-rate multipliers: (*υ*_1_,…,*υ*_*K*_).
*α*	The shape parameter of the gamma prior distribution on *υ*.
*ζ*_*D*1,*i*_,*ζ*_*D*2,*i*_	*θ*-scaling parameters that determine the magnitude of the population bottleneck in the 1^*st*^ and 2^*nd*^ descendant population of pair *i*,
	respectively. The bottleneck in each descendant population begins immediately after divergence.
***ζ***_***D1***_,***ζ***_***D2***_	Vectors (*ζ*_*D*1,1_,…,*ζ*_*D*1,*Y*_) and (*ζ*_*D*2,1_,…,*ζ*_*D*2,*Y*_), respectively.
*τ*_*B*,*i*_	Proportion of time between present and *T*_*i*_ when the bottleneck ends for the descendant populations of pair *i*.
***τ***_***B***_	Vector containing the *τ*_*B*_ parameters for each population pair: (*τ*_*B*,1_,…,*τ*_*B*,*Y*_).
m_*i*_	Symmetric migration rate between the descendant populations of pair *i*.
**m**	Vector containing the migration rates for each population pair: (*m*_*i*_,…,*m*_*Y*_).
*ρ*_*i*, *j*_	*θ*-scaling constant provided by the investigator for locus *j* of pair *i*. This constant is required to scale *θ* for differences in ploidy among loci
	or differences in generation times among taxa.
*ν*_*i*, *j*_	*θ*-scaling constant provided by the investigator for locus *j* of pair *i*. This constant is required to scale *θ* for differences in mutation rates
	among loci or among taxa.
***ρ***	Vector of ploidy and/or generation-time scaling constants: $\left(\rho_{1,\;1},\ldots ,\rho_{\text{Y},k_{Y}}\right)$
***ν***	Vector of mutation-rate scaling constants: $\left(\nu_{1,\;1},\ldots ,\nu_{\text{Y},k_{Y}}\right)$
$\bar{\text{T}}$	Mean of divergence times across the *Y* population pairs.
$s_{T}^{2}$	Variance of divergence times across the *Y* population pairs.
*D*_*T*_	Dispersion index of divergence times across the *Y* population pairs $\left(s_{T}^{2}/\bar{\text{T}}\right)$.
**n**	Number of samples from the joint prior.
*Λ* Vector of parameter values drawn from the joint prior.	
**S** Vector containing the summary statistics calculated from data simulated under parameter values drawn from the prior (*Λ*).	
***Λ*** Random sample of *Λ*_1_,…,*Λ*_**n**_ drawn form the prior.	
Summary statistic vectors *S*_1_,…,*S*_*n*_ for each *Λ*_1_,…,*Λ*_**n**_ drawn from the prior.	

I assume an investigator is interested in inferring the distribution of divergence times among *Y* pairs of populations. For each pair *i*, *n*_*i*_ genome copies have been sampled, with *n*_1,*i*_ copies sampled from population 1, and *n*_2,*i*_ sampled from population 2. From these genomes, let *k*_*i*_ be the number of DNA sequence loci collected for population pair *i*, and *K* be the total number of unique loci sampled across the *Y* pairs of populations. I use *X*_*i*, *j*_ to represent the multiple sequence alignment of locus *j* for population pair *i*. $X=(X_{1,1},\ldots ,X_{Y,k_{Y}})$ is the full dataset, i.e., a vector of sequence alignments for all pairs and loci. Let *G*_*i*, *j*_ represent the gene tree upon which *X*_*i*, *j*_ evolved according to fixed HKY85 substitution model parameters *ϕ*_*i*,*j*_. The investigator must specify the parameters of all $\phi =(\phi_{1,1},\ldots ,\phi_{\text{Y},k_{Y}})$ substitution models by which the alignments evolved along the $G=(G_{1,1},\ldots ,G_{\text{Y},k_{Y}})$ gene trees. Furthermore, the investigator must specify a vector of fixed constants $\rho =(\rho_{1,1},\ldots ,\rho_{\text{Y},k_{Y}})$ that scale the population-size parameters for known differences in ploidy among loci and/or differences in generation times among population pairs. Lastly, the investigator must also specify a vector of fixed constants $\nu =(\nu_{1,1},\ldots ,\nu_{\text{Y},k_{Y}})$ that scale the population-size parameters for known differences in mutation rates among loci and/or among taxa.

With **X**,***ϕ***,***ρ***, and ***ν*** in hand, the joint posterior distribution of the model is given by Bayes’ rule as

(1) $$\begin{array}{l} p(G,T,\Theta ,\upsilon ,\alpha |X,\phi ,\rho ,\nu )\\ \;=\frac{p(X|G,T,\Theta ,\upsilon ,\alpha ,\phi ,\rho ,\nu )p(G,T,\Theta ,\upsilon ,\alpha |\phi ,\rho ,\nu )}{p(X|\phi ,\rho ,\nu )} \end{array}$$

which can be expanded using the chain rule of probability into components that are assumed to be independent to get

(2) $$\begin{array}{l} p(G,T,\Theta ,\upsilon ,\alpha |X,\phi ,\rho ,\nu )\\ \;=\frac{p(X|G,\phi ),p(G|T,\Theta ,\upsilon ,\rho ,\nu )p(\upsilon |\alpha )p(\alpha )p(T)p(\Theta )}{p(X|\phi ,\rho ,\nu )}, \end{array}$$

where ***T***=(*T*_1_,…,*T*_*Y*_) is a vector of population divergence times for each of the *Y* pairs of populations, ***Θ***=(*Θ*_1_,…,*Θ*_*Y*_) is a vector of the demographic parameters for each of the *Y* population pairs, ***υ***=(*υ*_1_,…*υ*_*K*_) is a vector of locus-specific mutation-rate multipliers for each of the *K* loci, *α* is the shape parameter of a gamma-distributed prior on *υ*, and *p*(**X**|***ϕ***,***ρ***,***ν***), is the probability of the data (or the marginal likelihood of the model) given the fixed constants provided by the investigator.

To avoid calculating the likelihood terms of Equation 2, I distill each sequence alignment *X* into a vector of insufficient summary statistics *S*, thus replacing the full dataset $X=(X_{1,1},\ldots ,X_{Y,k_{Y}})$ with vectors of summary statistics for each alignment $S^{*}=\left({\text{S}}_{1,1}^{*},\ldots ,S_{Y,k_{Y}}^{*}\right)$ Optionally, for each population pair, the means of the summary statistics can be calculated across the *k* loci, and the vector can be further reduced to $S^{*}=\left(S_{1}^{*},\ldots ,S_{Y}^{*}\right)$. With **S**^∗^ in hand, we can estimate the approximate joint posterior distribution

(3) $$\begin{array}{l} p(G,T,\Theta ,\upsilon ,\alpha |{\text{B}}_{\varepsilon }(S^{*}),\phi ,\rho ,\nu )\\ \;\;\;=\frac{p({\text{B}}_{\varepsilon }(S^{*})|G,\phi )p(G|T,\Theta ,\upsilon ,\rho ,\nu )p(\upsilon |\alpha )p(\alpha )p(T)p(\Theta )}{p({\text{B}}_{\varepsilon }(S^{*})|\phi ,\rho ,\nu )}, \end{array}$$

where *B*_*ε*_(**S**^∗^) is the multidimensional Euclidean space around the vector of summary statistics, the radius of which is the tolerance *ε*. The sources of approximation are the insufficiency of the statistics and the *ε* being greater than zero. I describe the full model in detail before delving into the numerical method of estimating the approximate model.

#### Likelihood and gene-tree prior terms of Equation 2

The likelihood and gene-tree prior terms of Equation 2 can be expanded out as a product over population pairs and loci

(4) $$\begin{array}{l} p(X|G,\phi )p\left(G|T,\Theta ,\upsilon ,\rho ,\nu \right)\\ \;=\overset{Y}{\underset{i=1}{\prod }}\overset{k_{i}}{\underset{j=1}{\prod }}p\left(X_{i,\;j}|G_{i,\;j},\phi_{i,\;j}\right)p\left(G_{i,\;j}|T_{i},\Theta_{i},\upsilon_{j},\rho_{i,j},\nu_{i,j}\right). \end{array}$$

The first term, *p*(*X*_*i*, *j*_|*G*_*i*, *j*_,*ϕ*_*i*, *j*_), is the probability of the sequence alignment of locus *j* for population pair *i* given the gene tree and HKY85 [17] substitution model parameters [18, i.e., the “Felsenstein likelihood”]. The model allows for an intra-locus recombination rate *r*, which, for simplicity, is assumed to be zero in Equation 2. If *r* is non-zero, this term requires an additional product over the columns (sites) of each sequence alignment to allow sites to have different genealogies. The second term, p(*G*_*i*, *j*_|*T*_*i*_,*Θ*_*i*_,*υ*_*j*_,*ρ*_*i*, *j*_,*ν*_*i*, *j*_), is the probability of the gene tree under a multi-population coalescent model (i.e., species tree) where the ancestral population of pair *i* diverges and gives rise to the two sampled descendant populations. Each *Θ* contains the following demographic parameters: The mutation-rate-scaled effective sizes (*θ* = 4*Nμ*) of the ancestral, *θ*_*A*_, and descendant populations, *θ*_*D*1_ and *θ*_*D*2_; the proportion of the first, *ζ*_*D*1_, and second population, *ζ*_*D*2_, that persist during bottlenecks that begin immediately after divergence in forward-time; the proportion of time between present and divergence when the bottlenecks end for both populations, *τ*_*B*_; and the symmetric migration rate between the descendant populations, *m*. Thus, the probability of the *n*_*i*_−1 coalescence times (node heights) of gene tree *G*_*i*, *j*_ is given by a multi-population Kingman-coalescent model [19] where the ancestral population of size *θ*_*A*,*i*_*ρ*_*i*, *j*_*ν*_*i*, *j*_*υ*_*j*_ diverges at time *T*_*i*_ into two descendant populations of constant size *θ*_*D*1,*i*_*ρ*_*i*, *j*_*ν*_*i*, *j*_*υ*_*j*_*ζ*_*D*1,*i*_ and *θ*_*D*2,*i*_*ρ*_*i*, *j*_*ν*_*i*, *j*_*υ*_*j*_*ζ*_*D*2,*i*_, which, after time *T*_*i*_*τ*_*B*,*i*_, grow exponentially to their present size *θ*_*D*1,*i*_*ρ*_*i*, *j*_*ν*_*i*, *j*_*υ*_*j*_ and *θ*_*D*2,*i*_*ρ*_*i*, *j*_*ν*_*i*, *j*_*υ*_*j*_, respectively. Following divergence, the descendant populations of pair *i* exchange migrants at a symmetric rate of *m*_*i*_.

#### Additional prior terms of Equation 2

The term *p*(*α*) is the prior density function for the shape parameter of the gamma-distributed prior on rate heterogeneity among loci. This prior is *α*∼*U*(1,20). The prior probability of the vector of locus-specific mutation-rate multipliers given *α* then expands out as a product over the loci

(5) $$p(\upsilon |\alpha )=\overset{K}{\underset{j=1}{\prod }}p(\upsilon_{j}|\alpha ),$$

where each *υ* is independently and identically distributed (*iid*) as *υ*∼*Gamma*(*α*,1/*α*). If the recombination rate *r* is allowed to be non-zero, the prior term *p*(*r*) would be added to Equation 2, and the prior would be *r*∼*Gamma*(*a*_*r*_,*b*_*r*_), where *a*_*r*_ and *b*_*r*_ are specified by the investigator.

The prior term for the demographic parameters, *p*(***Θ***), expands out into its components and as a product over the *Y* pairs of populations

(6) $$\begin{array}{l} p(\Theta )=\overset{Y}{\underset{i=1}{\prod }}p(\theta_{A,i})p(\theta_{D1,i})p(\theta_{D2,i})p(\zeta_{D1,i})p(\zeta_{D2,i})p(\tau_{B,i})p(m_{i}). \end{array}$$

The priors for the demographic parameters are $\theta_{A}∼\text{Gamma}(a_{\theta_{A}},b_{\theta_{A}}),\theta_{D1}∼\text{Gamma}(a_{\theta_{D}},b_{\theta_{D}}),\theta_{D2}∼\text{Gamma}(a_{\theta_{D}},b_{\theta_{D}}),\zeta_{D1}∼\text{Beta}(a_{\zeta_{D}},b_{\zeta_{D}}),\zeta_{D2}∼\text{Beta}(a_{\zeta_{D}},b_{\zeta_{D}}),\tau_{B}∼U(0,1)$, and *m*∼*Gamma*(*a*_*m*_,*b*_*m*_), where the hyper-parameters of each prior distribution can be specified by the investigator. By default, *θ*_*A*_, *θ*_*D*1_, and *θ*_*D*2_ share the same prior (i.e., $a_{\theta_{A}}=a_{\theta_{D}}$ and $b_{\theta_{A}}=b_{\theta_{D}}$), but a separate gamma-distributed prior can be assigned to *θ*_*A*_. Also, the *ζ*_*D*1_,*ζ*_*D*2_, and *m* parameters are optional (i.e., the investigator can assume that there has been no migration between populations of each pair and/or the population size of each descendant population has been constant through time).

#### Priors on divergence models

The prior term for the vector of divergence times for each of the *Y* pairs of populations, ***T***, can be expanded as

(7) p(T)=p(t)p(τ|t),

where ***τ*** is an ordered set of divergence-time parameters {*τ*_1_,…,*τ*_|***τ***|_} whose length |***τ***| can range from 1 to *Y*, and **t** is a vector of indices (*t*_1_,…,*t*_*Y*_), where t_*i*_∈{1,…,|***τ***|}. These indices map each of the *Y* pairs of populations to a divergence-time parameter in ***τ***. Thus, ***T*** is the result of applying the mapping function

(8) $$f(\tau ,t,i)=\tau_{t_{i}}$$

to each population pair *i*, such that ***T*** = (*T*_1_ = *f*(***τ***,***t***,1),…,*T*_*Y*_=*f*(***τ***,***t***,*Y*)).

Biologically speaking, ***τ*** contains the times of divergence events, the length of which |***τ***| is the number of divergence events shared across the *Y* pairs of populations. For example, if ***τ*** contains a single divergence-time parameter *τ*_1_, all *Y* pairs of populations are constrained to diverge at this time (i.e., **t** would contain the index 1 repeated *Y* times, and ***T*** would contain the value *τ*_1_ repeated *Y* times), whereas if it contains *Y* divergence-time parameters, the model is fully generalized to allow all of the pairs to diverge at unique times.

Unlike the model implemented in msBayes, here I place priors on **t** and *τ*, rather than |***τ***| and *τ*. As a result, **t** determines the number of divergence-time parameters (|***τ***|) in the model. Below, I first describe the prior used for *τ* and the timescale it imposes on the model before discussing the priors implemented for **t**.

Each *τ* within ***τ*** is *iid* as *τ*∼*Gamma*(*a*_*τ*_,*b*_*τ*_), where *a*_*τ*_ and *b*_*τ*_ are specified by the investigator. Thus, given the number of unique divergence-time classes in **t**, this determines the probability of prior term *p*(***τ***|**t**). The divergence times are in coalescent units relative to the size of a constant reference population, which I denote *θ*_*C*_, that is equal to the expectation of the prior on the size of the descendant populations

(9) $$\theta_{C}=E(\theta_{D}),$$

Given the size of the descendant populations are *iid* as $\theta_{D1}∼\text{Gamma}(a_{\theta_{D}},b_{\theta_{D}})$ and $\theta_{D2}∼\text{Gamma}(a_{\theta_{D}},b_{\theta_{D}})$, this becomes

(10) $$\theta_{C}=a_{\theta_{D}}b_{\theta_{D}}.$$

More specifically, the *τ* parameters are in units of *θ*_*C*_/*μ* generations, which I denote as 4*N*_*C*_ generations. Thus, each *τ* within ***τ*** is proportional to time and can be converted to the number of generations of the reference population, which I denote $\tau_{G_{C}}$, by assuming a mutation rate and multiplying by the effective size of the reference population

(11) $$\tau_{G_{C}}=\tau \times \frac{\theta_{C}}{\mu }=\tau \times \frac{a_{\theta_{D}}b_{\theta_{D}}}{\mu }.$$

Thus, for each of the divergence times in ***τ*** to be on the same scale, the relative mutation rates among the pairs of populations are assumed to be known and fixed according to the user-provided values in ***ν***.

As described by Oaks et al. [7], to get the divergence times in units proportional to the expected number of mutations, they must be scaled by the realized population size for locus *j* of population-pair *i*

(12) $$T_{i,\;j}={\text{T}}_{i}\times \frac{\theta_{C}}{{\bar{\theta }}_{D,i}\rho_{i,j}},$$

where ${\bar{\theta }}_{D,i}$ is the mean of *θ*_*D*1_ and *θ*_*D*2_ for pair *i*. This gives us the vector of scaled divergence times $T=(T_{1,1},\ldots ,T_{\text{Y},k_{Y}})$.

As for the prior term *p*(**t**), the total sample space of **t** is all the possible partitionings of the *Y* pairs of populations into 1 to *Y* divergence-time classes, where each partitioning consists of non-overlapping and non-empty subsets whose union is the *Y* pairs. Hereinafter, I refer to these partitionings as “ordered” divergence models or partitions. The total number of possible partitions is a sum of the Stirling numbers of the second kind over all possible numbers of categories |***τ***|

(13) BY=∑|τ|=1Y1|τ|!∑j=0|τ|−1(−1)j|τ|j|τ|−jY,

which is the Bell number [20]. The original msBayes model samples over the unordered realizations of **t**, such that the sample space is reduced to all the possible integer partitions of *Y*[4,7,21-23] (Additional file 1: Table S1). I denote the set of all possible integer partitions of the *Y* pairs of populations as *a*(*Y*) and the length of that set as |*a*(*Y*)|, and I hereinafter refer to these integer partitions as “unordered” divergence models or partitions. The advantages, disadvantages, and justification of ignoring the order of **t** is discussed in detail below.

I implement two prior probability distributions over the space of all possible divergence models (**t**). The first simply gives all possible unordered partitions of *Y* elements equal probability

(14) $$p(t)=\frac{1}{\left|a(\text{Y})\right|},$$

i.e., a discrete uniform prior over all the integer partitions of *Y* (unordered divergence models). I denote this prior as **t**∼*DU*{*a*(*Y*)}.

The second prior is based on the Dirichlet process, which is a stochastic process that groups random variables into an unknown number of discrete parameter classes [24,25]. The Dirichlet process has been used as a non-parametric Bayesian approach to many inference problems in evolutionary biology [26-31]. Here, I use the Dirichlet process to place a prior over all possible ordered partitions of *Y* population pairs into divergence-time parameter classes (i.e., “divergence events”). As discussed above, the time of each divergence-time parameter is drawn from the base distribution *τ*∼*Gamma*(*a*_*τ*_,*b*_*τ*_). The partitioning of the population pairs to divergence-time classes is controlled by the concentration parameter *χ*, which determines how clustered the process will be. I take a hierarchical approach and use a prior probability distribution (i.e., hyperprior) for *χ*[32]. More specifically, I use a gamma-distributed prior *χ*∼*Gamma*(*a*_*χ*_,*b*_*χ*_), where *a*_*χ*_ and *b*_*χ*_ are specified by the investigator. I use **t**∼*DP*(*χ*) to denote this Dirichlet-process prior.

This provides a great deal of flexibility for specifying the prior uncertainty regarding divergence models. The concentration parameter *χ* determines the prior probability that any two pairs of populations *i* and *j* will be assigned to the same divergence-time parameter

(15) $$p(t_{i}=t_{j})=\frac{1}{1+\chi },$$

and also the prior probability of the number of divergence-time parameters

(16) $$p\left(|\tau ||\chi ,\text{Y}\right)=\frac{c(\text{Y},|\tau |)\chi^{\left|\tau \right|}}{\prod_{i=1}^{Y}\left(\chi +i-1\right)},$$

where *c*(·,·) are the unsigned Stirling numbers of the first kind. Equations 15 and 16 show that smaller values of *χ* will favor fewer divergence-time parameters, and thus more clustered models of divergence, whereas larger values will favor more divergence-time parameters, and thus less clustered models of divergence.

### Differences between this model and the original msBayes model

#### The prior on divergence models

One of the key differences between this model and that of msBayes[4] is the prior distribution on divergence models. As discussed in Oaks et al. [7], in msBayes the prior used for **t** is a combination of a discrete uniform prior over the possible number of divergence events |***τ***| from 1 to *Y* with a multinomial distribution on the number of times each index of ***τ*** appears in **t**, with the constraint that all *τ* parameters are represented at least once (see Equation two of [7]). I denote this prior used by msBayes as **t**∼*DU*{1,…,*Y*}. Oaks et al. [7] discuss how placing a uniform prior over the number of divergence parameters (denoted |***τ***| here, and as *Ψ* in [4]) imposes an “U-shaped” prior over divergence models (**t**; see Figure five(B) of [7]). To avoid this, I place priors directly on the sample space of divergence models, thus eliminating the parameter *Ψ* from the model. I introduce two priors on divergence models: (1) a prior that is uniform over all unordered divergence models, and (2) a Dirichlet-process prior on all ordered divergence models. The latter provides an investigator with a great deal of flexibility in expressing their prior beliefs about models of divergence.

#### Estimating ordered divergence models

As mentioned above, msBayes samples over unordered divergence models (i.e., unordered partitions of the *Y* pairs of populations). That is, the identity of each population pair, and all the information associated with it, is discarded. In my implementation, inference can be done on either unordered or ordered models of divergence. This is discussed in more detail in the description of the ABC implementation below.

#### The priors on nuisance parameters

I have replaced the use of continuous uniform distributions for priors on many of the model’s parameters (*τ*,*θ*_*A*_,*θ*_*D*1_,*θ*_*D*2_,*ζ*_*D*1_,*ζ*_*D*2_,*r*,*m*) with more flexible parametric distributions from the exponential family. I introduce gamma-distributed priors for rate parameters that have a sample space of all positive real numbers (*τ*,*θ*_*A*_,*θ*_*D*1_,*θ*_*D*2_,*r*,*m*), and beta-distributed priors for parameters that are proportions bounded by zero and one (*ζ*_*D*1_ and *ζ*_*D*2_). These priors provide an investigator with much greater flexibility in expressing prior uncertainty regarding the parameters of the model.

In addition, I have modified the prior on the sizes of the descendant populations of each pair. As described by Oaks et al. [7], msBayes uses the joint prior

(17) $$\theta_{D1},\theta_{D2}∼\text{Beta}(1,1)\times 2\times U(a_{\theta },b_{\theta_{D}}),$$

such that the user-specified uniform prior on descendant population size is a prior on the *mean* size of the two descendant populations of each pair. Under my model, the sizes of the descendant populations of each pair are *iid* as $\theta_{D1}∼\text{Gamma}(a_{\theta_{D}},b_{\theta_{D}})$ and $\theta_{D2}∼\text{Gamma}(a_{\theta_{D}},b_{\theta_{D}})$. This relaxes the assumption that the sizes of the two descendant populations are interdependent and negatively correlated.

#### Flexibility in parameterizing the model

In the new implementation, I provide the ability to control the richness of the model. For the *θ* parameters, by default, the model is fully generalized to allow each population pair to have three parameters: *θ*_*A*_,*θ*_*D*1_, and *θ*_*D*2_. Furthermore, if an investigator prefers to reduce the number of parameters, any model of *θ* parameters nested within this general model can also be specified, including the most restricted model where the ancestral and descendant populations of each pair share a single *θ* parameter.

I also provide the option of eliminating the parameters associated with the post-divergence bottlenecks in the descendant populations of each pair (*τ*_*B*_, *ζ*_*D*1_, and *ζ*_*D*2_), which constrains the descendant populations to be of constant size from present back to the divergence event. Also, rather than eliminate the bottleneck parameters, I allow *ζ*_*D*1_ and *ζ*_*D*2_ to be constrained to be equal, which removes one free parameter from the model for each of the population pairs.

Overall, my implementation allows an investigator to specify a model that has as many as seven parameters per population pair (*θ*_*A*_,*θ*_*D*1_,*θ*_*D*2_,*τ*_*B*_,*ζ*_*D*1_,*ζ*_*D*2_, and *m*) or as few as one parameter per pair (*θ*), in addition to the *n*_*i*_−1 coalescence-time parameters (i.e., the node heights of the gene tree).

#### Time scale

As described above, divergence times are in units of *θ*_*C*_/*μ* generations, where *θ*_*C*_ is the expectation of the prior on descendant-population size. As described by Oaks et al. [7], in msBayes, *θ*_*C*_ is half of the upper limit of the continuous uniform prior on the mean of the descendant population sizes. This is only equal to the expectation of the prior if the lower limit of the prior is zero.

### ABC estimation of the posterior of the model

#### Sampling from the prior

To estimate the approximate posterior of Equation 3, I use an ABC rejection algorithm. The first step of this algorithm entails collecting a random sample of parameter values from the joint prior and their associated summary statistics. Each sample is generated by (1) drawing values of all the model’s parameters, which I denote *Λ*, from their respective prior distributions; (2) simulating gene trees $G=(G_{1,1},\ldots ,G_{Y,k_{Y}})$ for each locus of each population pair by drawing coalescent times from a multi-population Kingman-coalescent model given the demographic parameters; (3) simulating sequence alignments $X=(X_{1,1},\ldots ,X_{Y,k_{Y}})$ along the gene trees under the HKY85 substitution parameters $\phi =(\phi_{1,1},\ldots ,\phi_{Y,k_{Y}})$ that have the same number of sequences and sequence lengths as the observed dataset; and (4) calculating population genetic summary statistics $S=(S_{1,1},\ldots ,S_{Y,k_{Y}})$ from the simulated sequence alignments. Optionally, an additional step can be performed to reduce the summary statistics to the means across loci for each population pair to get **S**=(*S*_1_,…,*S*_*Y*_). Either way, **S** contains the same summary statistics as those estimated from the observed data *S*^∗^. After repeating this procedure *n* times, we have a random sample of parameter vectors ***Λ***=(*Λ*_1_,…,*Λ*_**n**_) from the model prior and their associated vectors of summary statistics $S=(S_{1},\ldots ,S_{n})$.

For all of the analyses below, I use four summary statistics for each pair of populations: *π*[33], *θ*_*W*_[34], *π*_*net*_[35], and *SD*(*π*−*θ*_*W*_) [36]. Furthermore, in addition to model parameters, each sample *Λ* also contains four statistics that summarize **T**: the mean ($\bar{T}$), variance $\left(s_{T}^{2}\right)$, dispersion index ($D_{T}=s_{T}^{2}/\bar{T}$), and the number of divergence time parameters (|***τ***|). Previously, these have been denoted as *E*(*τ*)*Var*(*τ*),*Ω*, and *Ψ*, respectively [3,4,7]. I use $\bar{T}$ and $s_{T}^{2}$ in place *E*(*τ*) and *Var*(*τ*) to make clear that these values do not represent the prior or posterior expectation/variance of divergence times. I use *D*_*T*_ in place of *Ω* to clarify that this is a statistic rather than a parameter of the model. Lastly, I use |***τ***| in place of *Ψ*, because the number of divergence-time parameters is no longer a parameter in the new implementation.

#### Obtaining an approximate posterior from the prior samples

I use a rejection algorithm to retain an approximate posterior sample of *Λ* from the prior sample ***Λ***=(*Λ*_1_,…,*Λ*_**n**_). First, the observed summary statistics **S**^∗^, and the summary statistics of the prior samples $S=(S_{1},\ldots ,S_{n})$, are standardized using the means and standard deviations of the statistics from the prior sample (i.e., the prior mean is subtracted from each statistic, and the difference is divided by the prior standard deviation). After all statistics are standardized, the Euclidean distance between **S**^∗^ and each **S** within  is calculated. The samples that fall within a range of tolerance *ε* around **S**^∗^ are retained. The range of tolerance is determined by specifying the desired number of posterior samples to be retained. Post-hoc adjustment of the posterior sample can also be performed with a number of regression techniques [37-39]. For analyses below, I use the general linear model (GLM) regression adjustment [39] as implemented in ABCtoolbox v1.1 [40], which Oaks et al. [7] showed performs very similarly to weighted local-linear regression and multinomial logistic regression adjustments [37] for msBayes posteriors.

#### Ordering of taxon-specific summary statistics

As alluded to in the model description, msBayes does not maintain the order of the taxon-specific summary statistics *S* within each **S**. Rather, the summary statistics are re-ordered by descending values of average pairwise differences between the descendant populations (*π*_*b*_) [4,41]. This has the advantage of reducing the sample space of possible divergence models **t**, but there are at least two disadvantages. First, additional information in the data is lost. By discarding the identity of the *Y* pairs of populations, all pair-specific information about the amount of data (e.g., the number of gene copies collected from each of the populations [*n*_1_ and *n*_2_], the number of loci, and the length of the loci), and the taxon- and locus-specific parameters (*ϕ*,*ν*,*ρ*, and *υ*) is lost. Second, the results are more difficult to interpret, because divergence models and parameter estimates cannot be directly associated to the taxa under study.

The re-ordering of the summary statistic vectors also has an important implication for the ABC algorithm. When calculating the Euclidean distance between the observed data and each simulated dataset, the summary statistics being compared often represent sequence alignments of *different* taxon pairs and/or loci. More specifically, the summary statistics calculated from the observed sequence alignments are being compared to summary statistics calculated from datasets simulated with potentially *different* (1) numbers of sequences (*n*_1_ and *n*_2_), (2) length of alignments, (3) numbers of loci (*k*), (4) HKY85 model parameters (*ϕ*), (5) mutation-rate multipliers (*ν*), and (6) ploidy multipliers (*ρ*).

In the original descriptions of the msBayes method [3,4], this re-ordering is justified by the fact that the expected value of *π*_*b*_ is unrelated to sample size *n*_1_ and *n*_2_ and thus exchangeable among pairs. This is incorrect for two reasons. First, the entire vector of summary statistics *S* for each pair of populations is re-ordered across pairs, which implies that the justification for re-ordering *π*_*b*_ applies to all the statistics within each *S*. However, the expectations for statistics that estimate gross diversity (e.g., *π* and *θ*_*W*_) are not independent of sample size for structured populations (e.g., the divergent pairs of populations modeled by msBayes), and other statistics are not independent of sample size in general (e.g., *SD*(*π*−*θ*_*W*_)). Second, and more importantly, having the same expectation does not ensure random variables are exchangeable. Rather, for variables to be exchangeable their marginal distributions must be the same (i.e., they must be identically distributed). *None* of the summary statistics used by msBayes, including *π*_*b*_, have this property when there is any variation among taxa or loci in the (1) numbers of sequences (*n*_1_ and *n*_2_), (2) length of alignments, (3) numbers of loci (*k*), (4) HKY85 model parameters (*ϕ*), (5) mutation-rate multipliers (*ν*), or (6) ploidy multipliers (*ρ*). Whenever such variation is present (i.e., nearly all empirical applications), the taxon-specific summary statistics *S* are not exchangeable, and the reshuffling of the summary statistic vectors is not mathematically valid.

The magnitude of the affect of this violation of exchangeability is not known. Huang et al. [4] demonstrated that the reordering of the summary statistic vectors can greatly increase the method’s tendency to infer a single divergence event. By definition, if the summary statistic vectors were exchangeable, the reordering would not change the likelihood or posterior (barring sampling error). Thus, the results of Huang et al. [4] suggest the reordering of the statistics is potentially introducing sizeable error to the analysis.

For comparability with msBayes, I maintain the option for re-ordering taxon-specific summary statistics by *π*_*b*_. However, by default, the order is preserved, and ordered divergence models are estimated. In all of the simulation-based analyses described below, the summary statistic vectors *are* exchangeable, because the simulated datasets have the same (1) numbers of sequences, (2) length of sequences, (3) numbers of loci, (4) HKY85 model parameters, (5) mutation-rate multipliers, and (6) ploidy multipliers.

### Assessing model-choice behavior and robustness

Following the simulation-based approach of Oaks et al. [7], I characterize the behavior of several models under the ideal conditions where the data are generated from parameters drawn from the same prior distributions used for analysis (i.e., the prior is correct). I selected the following four model priors for these analyses (Table 2). 

1. The *M*_*msBayes*_ model represents the original msBayes implementation with the U-shaped prior on unordered divergence models and uniform priors on divergence-time and demographic parameters; **t**∼*DU*{1,…,*Y*},*τ*∼*U*(0,10),*θ*_*A*_∼*U*(0,0.05), and ${\bar{\theta }}_{D}∼U(0,0.05)$.

**Table 2:** The models evaluated in the simulation-based analyses

	**Priors**			
**Model**	**t**	***τ***	***θ***	
*M*_*m* *s* *B* *a* *y* *e* *s*_	**t**∼*DU*{1,…,*Y*}	*τ*∼*U*(0,10 [ 25 *MGA*])	*θ*_*A*_∼*U*(0,0.05)	${\bar{\theta }}_{D}∼U(0,0.05)$
*M*_*U* *s* *h* *a* *p* *e* *d*_	**t**∼*DU*{1,…,*Y*}	*τ*∼*Exp*(*mean*=2.887[ 7.22*MGA*])	*θ*_*A*_∼*θ*_*D*1_∼*θ*_*D*2_∼*Exp*(*mean*=0.025)	
*M*_*U* *n* *i* *f* *o* *r* *m*_	**t**∼*DU*{*a*(*Y*)}	*τ*∼*Exp*(*mean*=2.887[ 7.22*MGA*])	*θ*_*A*_∼*θ*_*D*1_∼*θ*_*D*2_∼*Exp*(*mean*=0.025)	
*M*_*D* *P* *P*_	**t**∼*DP*(*χ*∼*Gamma*(·,·))	*τ*∼*Exp*(*mean*=2.887[ 7.22*MGA*])	*θ*_*A*_∼*θ*_*D*1_∼*θ*_*D*2_∼*Exp*(*mean*=0.025)	

For the *M*_*DPP*_ model, the prior on the concentration parameter, ***χ∼****Gamma****(·,·)***, was set to *Gamma*(2,2) for the validation analyses and *Gamma*(1.5,18.1) for the power analyses. The distributions of divergence times are given in units of 4*N*_*C*_ generations followed in brackets by units of millions of generations ago (MGA), with the former converted to the latter assuming a per-site rate of 1***×***10^−8^ mutations per generation. For model *M*_*msBayes*_, the priors for theta parameters are ***θ***_*A*_***∼****U*(0, 0.05) and ***θ***_*D*1_,***θ***_*D*2_***∼****Beta*(1, 1)***×***2***×****U*(0, 0.05). The later is summarized as ${\bar{\theta }}_{\;\text{D}}∼\text{U}(\text{0, 0.05})$. For the *M*_*DPP*_ and M_*Uniform*_, and *M*_*Ushaped*_ models, ***θ***_*A*_,***θ***_*D*1_, and ***θ***_*D*2_ are independently and exponentially distributed with a mean of 0.025.

2. The *M*_*Ushaped*_ model with the U-shaped prior of msBayes on unordered divergence models, but with exponential priors on divergence-time and demographic parameters; **t**∼*DU*{1,…,*Y*},*τ*∼*Exp*(*mean*=2.887),*θ*_*A*_∼*Exp*(*mean*=0.025),*θ*_*D*1_∼*Exp*(*mean*=0.025), and *θ*_*D*2_∼*Exp*(*mean*=0.025).

3. The *M*_*Uniform*_ model with a uniform prior over unordered divergence models and exponential priors on divergence-time and demographic parameters; **t**∼*DU*{*a*(*Y*)},*τ*∼*Exp*(*mean*=2.887),*θ*_*A*_∼*Exp*(*mean*=0.025),*θ*_*D*1_∼*Exp*(*mean*=0.025), and *θ*_*D*2_∼*Exp*(*mean*=0.025).

4. The *M*_*DPP*_ model with a Dirichlet-process prior on ordered divergence models and exponential priors on divergence-time and demographic parameters; **t** ∼ *DP*(*χ*∼*Gamma*(2,2)),*τ* ∼ *Exp*(*mean*=2.887), *θ*_*A*_ ∼ *Exp*(*mean*=0.025),*θ*_*D*1_ ∼ *Exp*(*mean*=0.025), and *θ*_*D*2_∼*Exp*(*mean*=0.025).

I selected the exponential prior on divergence time used in models *M*_*DPP*_,*M*_*Uniform*_, and *M*_*Ushaped*_ to have the same variance as the uniform prior in model *M*_*msBayes*_. I selected the exponential prior on population size used in models *M*_*DPP*_, *M*_*Uniform*_, and *M*_*Uniform*_ to have the same mean as the uniform prior in model *M*_*msBayes*_, so that all four models have the same *θ*_*C*_ and thus the same units of time. All of the models were the same in other respects, with three free *θ* parameters for each population pair, two uniformly distributed (*beta*(1,1)) *ζ*_*D*_ parameters per pair, no migration, no recombination, and re-sorting of taxon-specific summary statistics by *π*_*b*_ (i.e., sampling unordered divergence models). For all simulations, I used a data structure of eight population pairs, with a single 1000 base-pair locus sampled from 10 individuals from each population.

For each of the four models, I simulated 1×10^6^ samples from the prior and 50,000 datasets, also drawn from the prior. I then analyzed each of the simulated datasets, retaining a posterior of 1000 samples from the respective prior. A GLM-regression adjusted posterior was also estimated from each of the posterior samples [39]. To assess the robustness of each of the four models, I also analyzed the datasets simulated under the other three models. Overall, for each model, I produced 200,000 posterior estimates, 50,000 from the datasets simulated under that model, and 150,000 from the datasets simulated under the other three models.

For each set of 50,000 simulated datasets, I used the posterior estimates to assess the model-choice behavior of each model. I did this by assigning the 50,000 estimates of the posterior probability of one-divergence event to 20 bins of width 0.05, and plotted the estimated posterior probability of each bin against the proportion of replicates in that bin with a true value consistent with one divergence event [7,42]. Ideally, the estimated posterior probability of the one-divergence model should estimate the probability that the one-divergence model is correct. For large numbers of simulation replicates, the proportion of the replicates in each bin for which the one-divergence model is true will approximate the probability that the one-divergence model is the correct model. Thus, if the method has the desirable behavior such that the estimated posterior probability of the one-divergence model is an unbiased estimate of the probability that the one-divergence model is correct, the points should fall near the identity line. For example, let us say the method estimates a posterior probability of 0.90 for 1000 datasets simulated from the prior. If the method is accurately estimating the probability that the one-divergence model is correct given the data, then the one-divergence model should be the true model in approximately 900 of the 1000 replicates. Any trend away from the identity line indicates the method is biased in the sense that it is not accurately estimating the probability that the one-divergence model is the correct model.

I constructed these plots using two criteria for the one-divergence model: (1) the number of divergence-time parameters (|***τ***|=1) and (2) the dispersion index of divergence times (*D*_*T*_<0.01). For the latter, *D*_*T*_<0.01 has been commonly used as an arbitrary criterion for a single “simultaneous” divergence event (e.g., [3,5,6]). I focused on the one-divergence model to assess model-choice behavior, because it is often of biogeographic interest and is easily comparable among the three different priors used on divergence models.

In addition to the four models above, I also assessed the behavior of a model that samples over ordered divergence models (i.e., the order of the taxon-specific summary statistic vectors were maintained for the observed and simulated datasets); all other settings were identical to the *M*_*DPP*_ model. I denote this model as *M*°_*DPP*_. I simulated 1×10^6^ prior samples and 50,000 datasets, and analyzed them as above. I was not able to analyze the simulated datasets of the other models under the ordered model, because the identity of the population pairs is not contained in the simulations of the other models.

### Assessing power

I evaluated the power of the same four models (Table 2) to detect random variation in divergence times using methods similar to Oaks et al. [7]. For all power simulations, I used a data structure identical to that of the empirical dataset of Philippine vertebrates analyzed by Oaks et al. [7], which consists of 22 pairs of populations. Due to the larger number of pairs, I used a different hyperprior on the concentration parameter for the *M*_*DPP*_ model; I used a prior of **t**∼*DP*(*χ*∼*Gamma*(1.5,18.1)) over divergence models for the model *M*_*DPP*_. All other aspects of the four models in Table 2 were identical to those used in the validation analyses described above. For each of the four models, I generated 2 × 10^6^ samples from the prior.

Next, I simulated datasets from three series of models in which the divergence times of the 22 pairs were random (i.e., no clustering; |***τ***|=22). The models comprising each series differ in the variance of the distribution from which the divergence times are randomly drawn. When the variance of random divergence times is small, all of the models in Table 2 are expected to struggle to detect this variation and will often incorrectly estimate highly clustered models of divergence (i.e., few divergence events). The goal is to assess how much temporal variation in random divergence times is necessary before the behavior of the models of Table 2 begins to improve. This will determine the timescales over which the models can reliably detect random variation in divergence times and avoid spurious inference of clustered divergence models.

Specifically, I simulated datasets from the following three series of six models (Table 3). 

1. The ${ℳ}_{\text{msBayes}}$ models are identically distributed as *M*_*msBayes*_ except the divergence times for each of the 22 pairs of populations are randomly drawn from a series of uniform distributions, *U*(0,*τ*_*max*_), where *τ*_*max*_ was set to: 0.2, 0.4, 0.6, 0.8, 1.0, and 2.0, in 4*N*_*C*_ generations.

**Table 3:** **The models used to simulate pseudo-replicate datasets for assessing the power of the models in Table**2

	**Priors**			
**Model series**	**t**	***τ***	***θ***	
${ℳ}_{\text{msBayes}}$	|***τ***|=22	*τ*∼*U*(0,0.2 [ 0.5 *MGA*])	*θ*_*A*_∼*U*(0,0.05)	${\bar{\theta }}_{D}∼U(0,0.05)$
	|***τ***|=22	*τ*∼*U*(0,0.4 [ 1.0 *MGA*])	*θ*_*A*_∼*U*(0,0.05)	${\bar{\theta }}_{D}∼U(0,0.05)$
	|***τ***|=22	*τ*∼*U*(0,0.6 [ 1.5 *MGA*])	*θ*_*A*_∼*U*(0,0.05)	${\bar{\theta }}_{D}∼U(0,0.05)$
	|***τ***|=22	*τ*∼*U*(0,0.8 [ 2.0 *MGA*])	*θ*_*A*_∼*U*(0,0.05)	${\bar{\theta }}_{D}∼U(0,0.05)$
	|***τ***|=22	*τ*∼*U*(0,1.0 [ 2.5 *MGA*])	*θ*_*A*_∼*U*(0,0.05)	${\bar{\theta }}_{D}∼U(0,0.05)$
	|***τ***|=22	*τ*∼*U*(0,2.0 [ 5.0 *MGA*])	*θ*_*A*_∼*U*(0,0.05)	${\bar{\theta }}_{D}∼U(0,0.05)$
${ℳ}_{\text{Uniform}}$	|***τ***|=22	*τ*∼*U*(0,0.2 [ 0.5 *MGA*])	*θ*_*A*_∼*θ*_*D*1_∼*θ*_*D*2_∼*Exp*(*mean*=0.025)	
	|***τ***|=22	*τ*∼*U*(0,0.4 [ 1.0 *MGA*])	*θ*_*A*_∼*θ*_*D*1_∼*θ*_*D*2_∼*Exp*(*mean*=0.025)	
	|***τ***|=22	*τ*∼*U*(0,0.6 [ 1.5 *MGA*])	*θ*_*A*_∼*θ*_*D*1_∼*θ*_*D*2_∼*Exp*(*mean*=0.025)	
	|***τ***|=22	*τ*∼*U*(0,0.8 [ 2.0 *MGA*])	*θ*_*A*_∼*θ*_*D*1_∼*θ*_*D*2_∼*Exp*(*mean*=0.025)	
	|***τ***|=22	*τ*∼*U*(0,1.0 [ 2.5 *MGA*])	*θ*_*A*_∼*θ*_*D*1_∼*θ*_*D*2_∼*Exp*(*mean*=0.025)	
	|***τ***|=22	*τ*∼*U*(0,2.0 [ 5.0 *MGA*])	*θ*_*A*_∼*θ*_*D*1_∼*θ*_*D*2_∼*Exp*(*mean*=0.025)	
${ℳ}_{\text{Exp}}$	|***τ***|=22	*τ*∼*Exp*(*mean*=0.058 [ 0.14 *MGA*])	*θ*_*A*_∼*θ*_*D*1_∼*θ*_*D*2_∼*Exp*(*mean*=0.025)	
	|***τ***|=22	*τ*∼*Exp*(*mean*=0.115 [ 0.29 *MGA*])	*θ*_*A*_∼*θ*_*D*1_∼*θ*_*D*2_∼*Exp*(*mean*=0.025)	
	|***τ***|=22	*τ*∼*Exp*(*mean*=0.173 [ 0.43 *MGA*])	*θ*_*A*_∼*θ*_*D*1_∼*θ*_*D*2_∼*Exp*(*mean*=0.025)	
	|***τ***|=22	*τ*∼*Exp*(*mean*=0.231 [ 0.58 *MGA*])	*θ*_*A*_∼*θ*_*D*1_∼*θ*_*D*2_∼*Exp*(*mean*=0.025)	
	|***τ***|=22	*τ*∼*Exp*(*mean*=0.289 [ 0.72 *MGA*])	*θ*_*A*_∼*θ*_*D*1_∼*θ*_*D*2_∼*Exp*(*mean*=0.025)	
	|***τ***|=22	*τ*∼*Exp*(*mean*=0.577 [ 1.44 *MGA*])	*θ*_*A*_∼*θ*_*D*1_∼*θ*_*D*2_∼*Exp*(*mean*=0.025)	

The distributions of divergence times are given in units of 4*N*_*C*_ generations followed in brackets by units of millions of generations ago (MGA), with the former converted to the latter assuming a per-site rate of 1 ***×*** 10^−8^ mutations per generation. For all of the ${ℳ}_{\text{msBayes}}$ models, the priors for theta parameters are ***θ***_*A*_***∼****U*(0, 0.05) and ***θ***_*D*1_,***θ***_*D*2_***∼****Beta*(1, 1)***×***2***×****U*(0, 0.05. The later is summarized as ${\bar{\theta }}_{\;\text{D}}∼\text{U}(\text{0, 0.05})$. For the ${ℳ}_{\text{Uniform}}$ and ${ℳ}_{\text{Exp}}$ models, ***θ***_*A*_,***θ***_*D*1_, and ***θ***_*D*2_ are independently and exponentially distributed with a mean of 0.025.

2. The ${ℳ}_{\text{Uniform}}$ models are identically distributed as *M*_*Uniform*_ and *M*_*DPP*_ except the 22 divergence times are randomly drawn from the same series of uniform priors as above.

3. The ${ℳ}_{\text{Exp}}$ models are also identically distributed as *M*_*Uniform*_ and *M*_*DPP*_ except the 22 divergence times are randomly drawn from a series of of exponential distributions: *Exp*(*mean*=0.058), *Exp*(*mean*=0.115), *Exp*(*mean*=0.173), *Exp*(*mean*=0.231), *Exp*(*mean*=0.289), and *Exp*(*mean*=0.577). These exponential distributions have the same variance as their uniform counterparts in the first two series of models.

For each of the six models in each of the three series of models, I simulated 1000 datasets (18,000 datasets in total). I then analyzed each simulated dataset under all four prior models (Table 2), producing 72,000 posterior estimates, each with 1000 samples. I also estimated a GLM-regression adjusted posterior from each of the posterior samples [39].

### An empirical application

I also assessed the behavior of the newly implemented models when applied to the empirical dataset of Oaks et al. [7], which is comprised of sequence data from 22 pairs of taxa from the Philippine Islands ([43]; Dryad DOI: 10.5061/dryad.5s07m). I analyzed these data under five different models, which are detailed in Table 4. All of these models except one ($M_{\text{DPP}}^{\text{simple}}$) have six free demographic parameters per pair of taxa (*θ*_*A*_,*θ*_*D*1_,*θ*_*D*2_,*τ*_*B*_,*ζ*_*D*1_,and *ζ*_*D*2_), in addition to the *n*_*i*_−1 coalescent times. Three of these models use a Dirichlet-process prior on divergence models: $M_{\text{DPP}},M_{\text{DPP}}^{\text{inform}},\text{and}\;M_{\text{DPP}}^{\text{simple}}$. The **M**_*DPP*_ model represents the priors that Oaks et al. [7] would have selected to reflect their prior uncertainty about the parameters of the model if provided the more flexible distributions that are now implemented. To assess prior sensitivity, the $M_{\text{DPP}}^{\text{inform}}$ model uses a more informative exponentially distributed prior on divergence times, but otherwise is identical to **M**_*DPP*_. To assess sensitivity to parameterization, I also applied the simplest possible model under the new implementation $\left(M_{\text{DPP}}^{\text{simple}}\right)$ with only a single demographic parameter (*θ*) per taxon pair, in addition to the *n*_*i*_−1 coalescent times. I also applied the original msBayes model (**M**_*msBayes*_) with priors selected to make the results directly comparable to those of the **M**_*DPP*_ model; the uniform prior on divergence times was selected to have the same variance as the exponential prior of the **M**_*DPP*_ model, and the prior on population size was selected to have the same mean so that the models are on the same timescale. I also applied a model with a uniform distribution over divergence models (**M**_*Uniform*_). For each of these models, I simulated 2 × 10^7^ samples from the prior, and retained an approximate posterior of the 10,000 samples with the smallest Euclidean distance from the summary statistics calculated from the empirical sequence alignments.

**Table 4:** **The models used to analyze the data from the 22 pairs of taxa from the Philippines (M), and a subset of nine of those pairs from the Islands of Negros and Panay (** M**)**

**Model**	**Priors**
**M**_*m* *s* *B* *a* *y* *e* *s*_	**t**∼*DU*{1,…,*Y*} *τ*∼*U*(0,34.64 [ 17.3 *MGA*]) *θ*_*A*_∼*U*(0,0.01) *θ*_*D*1_,*θ*_*D*2_∼*Beta*(1,1)×2×*U*(0,0.01) *ζ*_*D*1_∼*U*(0,1) *ζ*_*D*2_∼*U*(0,1)
**M**_*U* *n* *i* *f* *o* *r* *m*_	**t**∼*DU*{*a*(*Y*)} *τ*∼*Exp*(*mean*=10 [ 5 *MGA*]) *θ*_*A*_∼*Exp*(*mean*=0.005) *θ*_*D*1_∼*Exp*(*mean*=0.005) *θ*_*D*2_∼*Exp*(*mean*=0.005)
	*ζ*_*D*1_∼*Beta*(5,1) *ζ*_*D*2_∼*Beta*(5,1)
**M**_*D* *P* *P*_	**t**∼*DP*(*χ*∼*Gamma*(1.5,18.1)) *τ*∼*Exp*(*mean*=10 [ 5 *MGA*]) *θ*_*A*_∼*Exp*(*mean*=0.005) *θ*_*D*1_∼*Exp*(*mean*=0.005)
	*θ*_*D*2_∼*Exp*(*mean*=0.005) *ζ*_*D*1_∼*Beta*(5,1) *ζ*_*D*2_∼*Beta*(5,1)
$M_{\text{DPP}}^{\text{inform}}$	**t**∼*DP*(*χ* ∼*Gamma*(1.5,18.1)) *τ* ∼*Exp*(*mean*=6 [ 3 *MGA*]) *θ*_*A*_ ∼*Exp*(*mean*=0.005) *θ*_*D*1_ ∼*Exp*(*mean*=0.005)
	*θ*_*D*2_ ∼*Exp*(*mean*=0.005) *ζ*_*D*1_∼*Beta*(5,1) *ζ*_*D*2_∼*Beta*(5,1)
$M_{\text{DPP}}^{\text{simple}}$	**t**∼*DP*(*χ*∼*Gamma*(1.5,18.1)) *τ*∼*Exp*(*mean*=10 [ 5 *MGA*]) *θ*_*A*_=*θ*_*D*1_=*θ*_*D*2_∼*Exp*(*mean*=0.005) *ζ*_*D*1_=*ζ*_*D*2_=1.0
$M_{\text{DPP}}$	**t**∼*DP*(*χ*∼*Gamma*(1.5,5.0)) *τ*∼*Exp*(*mean*=10 [ 5 *MGA*]) *θ*_*A*_∼*Exp*(*mean*=0.005) *θ*_*D*1_=*θ*_*D*2_∼*Exp*(*mean*=0.005)
	*ζ*_*D*1_=*ζ*_*D*2_=1.0

In addition to the *n****−***1 coalescent times, the $M_{\text{DPP}}^{\text{simple}}$ has only a single ***θ*** parameter for each taxon pair. The remaining **M** models have three ***θ***, two ***ζ***_*D*_, and one ***τ***_*B*_ parameter. The distributions of divergence times are given in units of 4*N*_*C*_ generations followed in brackets by units of millions of generations ago (MGA), with the former converted to the latter assuming a per-site rate of 1 ***×*** 10^−8^ mutations per generation. The $M_{\text{DPP}}$ model (and its $M_{\text{DPP}}^{\circ }$ counterpart that samples over ordered divergence models) has only two ***θ*** parameters (the descendant populations of each pair share the same ***θ*** parameter, and there are no bottleneck parameters).

To compare models that sample over ordered versus unordered models of divergence, I also analyzed the data from the subset of nine-taxon pairs that are sampled from the Islands of Negros and Panay in the Philippines. The model I used for these analyses had a Dirichlet-process prior over divergence models and two demographic parameters (*θ*_*A*_ and (*θ*_*D*_) for each pair of taxa, in addition to the *n*_*i*_−1 coalescent times (see Table 4 for details). One of the models, which I denote $M{\text{°}}_{\text{DPP}}$, maintained the identity of the taxon pairs and sampled over ordered models of divergence, while the other ($M_{\text{DPP}}$) re-sorted the summary statistics of the pairs by *π*_*b*_, losing the identity of the taxa and thus sampled over unordered models of divergence. For both analyses, I simulated 5 ×10^7^ samples from the prior and retained an approximate posterior of 10,000 samples.

## Results

### Validation analyses: Estimation accuracy

In terms of estimating the variance of divergence times (*D*_*T*_), the models with exponentially distributed priors (*M*_*Ushaped*_, *M*_*Uniform*_, and *M*_*DPP*_) perform similarly when applied to datasets generated under all four of the models in Table 2 (Additional file 1: Figure S1). The *M*_*msBayes*_ model performs similarly to these models when applied to its own datasets, but is sensitive to model violations and is more biased when applied to data generated under the other three models (Additional file 1: Figure S1). Results are similar for the GLM-adjusted estimates of *D*_*T*_, albeit the regression adjustment tends to improve estimates of this continuous statistic for all the models (Additional file 1: Figure S2).

The same general pattern is seen for estimates of $\bar{T}$, with (1) all four models performing similarly when applied to the data generated under the *M*_*msBayes*_ model, (2) the models with exponentially distributed priors performing similarly when applied to data generated under the other three models, and (3) the *M*_*msBayes*_ model is sensitive to model violations and is more biased whenever applied to data generated under other models (Additional file 1: Figure S3). Also, the regression adjustment tends to slightly improve estimates of this continuous statistic for all of the models (Additional file 1: Figure S4).

In terms of estimating the number of divergence events (|***τ***|), the *M*_*DPP*_ model has the lowest root mean square error (RMSE) when applied to data generated under most of the models of Table 2 (Additional file 1: Figure S5). The *M*_*msBayes*_ model performs slightly better when applied to its own data, but is the worst performer when applied to data generated under other models (Additional file 1: Figure S5). There is a trend of *M*_*DPP*_>*M*_*Uniform*_>*M*_*Ushaped*_>*M*_*msBayes*_ in terms of estimation accuracy as measured by RMSE when the models are applied to data generated under most of the models (Additional file 1: Figure S5). Unlike for the continuous statistics, regression adjustment of this discrete statistic tends to increase estimation bias; all of the models tend to underestimate |***τ***| after the GLM-adjustment (Additional file 1: Figure S6).

### Validation analyses: Model-choice accuracy

**Figure 1:**
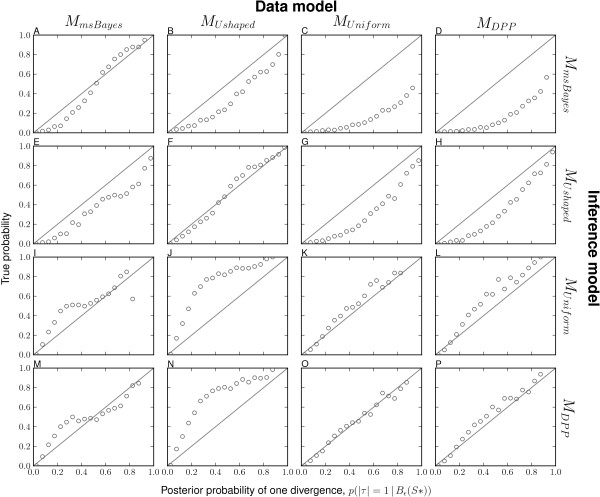
**Comparison of model-choice accuracy.** Model-choice accuracy for models **(A–D)***M*_*msBayes*_, **(E–H)***M*_*Ushaped*_, **(I–L)***M*_*Uniform*_, and **(M–P)***M*_*DPP*_ when analyzing data generated under models **(A, E, I, and M)***M*_*msBayes*_, **(B, F, J, and N)***M*_*Ushaped*_, **(C, G, K, and O)***M*_*Uniform*_, and **(D, H, L, and P)***M*_*DPP*_. The unadjusted posterior probability of a single divergence event, based on |***τ***|=1, from 50,000 posterior estimates are assigned to bins of width 0.05 and plotted against the proportion of replicates in each bin where the truth is |***τ***|=1.

**Figure 2:**
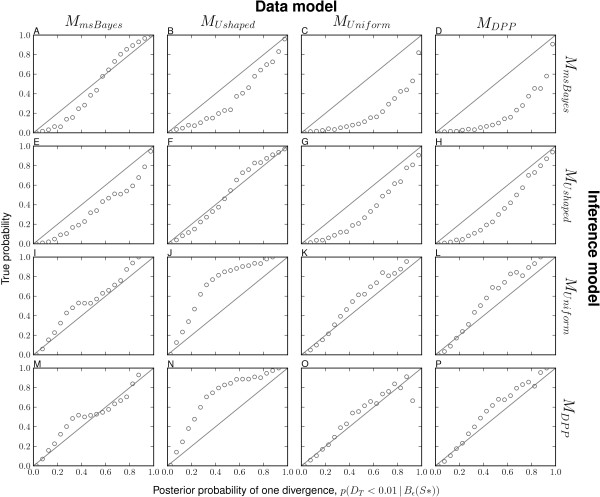
**Comparison of model-choice accuracy using*D***_***T***_** threshold.** Model-choice accuracy for models **(A–D)***M*_*msBayes*_, **(E–H)***M*_*Ushaped*_, **(I–L)***M*_*Uniform*_, and **(M–P)***M*_*DPP*_ when analyzing data generated under models **(A, E, I, and M)***M*_*msBayes*_, **(B, F, J, and N)***M*_*Ushaped*_, **(C, G, K, and O)***M*_*Uniform*_, and **(D, H, L, and P)***M*_*DPP*_. The unadjusted posterior probability of a single divergence event, based on *D*_*T*_<0.01, from 50,000 posterior estimates are assigned to bins of width 0.05 and plotted against the proportion of replicates in each bin where the truth is *D*_*T*_<0.01.

The msBayes model, and my modification of it, is a model-choice method with the primary purpose of estimating the probabilities of models of divergence across taxa. Thus, it is critical to assess the method’s ability to accurately estimate the posterior probabilities of divergence models. Consistent with the findings of Oaks et al. [7], my results demonstrate that the unadjusted estimates of divergence-model posterior probabilities are generally more accurate than regression-adjusted estimates (compare the plots along the upper-left to lower-right diagonal for Figure 1 versus Additional file 1: Figure S7 and Figure 2 versus Additional file 1: Figure S8). Regression adjustment results in biased estimates of the posterior probability of the one-divergence model when all model assumptions are satisfied, which is well illustrated in Additional file 1: Figure S8. As a result, I will focus my discussion of the results on the unadjusted estimates.

I find that all four models accurately estimate the posterior probability of the one-divergence model when applied to their own datasets (i.e., when the prior is correct; see diagonal of Figures 1 and 2). The *M*_*Uniform*_ and *M*_*DPP*_ models show robustness to prior violations and perform well when applied to data generated under other models (Figures 1 and 2). However, both are less accurate and tend to underestimate the probability of the one-divergence model when applied to the data generated under *M*_*Ushaped*_ (Figures 1 and 2). In contrast, the *M*_*msBayes*_ model is biased toward overestimating the posterior probability of the one-divergence model when applied to data generated under the other three models (Figures 1 and 2). This bias is particularly strong whenever divergence models are not distributed under its U-shaped prior (Figure 1C–D). The other model with the U-shaped prior on divergence models, but exponential priors on parameters (*M*_*Ushaped*_), performs similarly to the *M*_*msBayes*_ model in that it performs well when applied to its own data, but overestimates the probability of the one-divergence model when applied to data generated by the other models (Figures 1 and 2). However, the bias is stronger in the *M*_*msBayes*_ model than *M*_*Ushaped*_.

Overall, the results suggest that the *M*_*DPP*_ and *M*_*Uniform*_ models are relatively robust in terms of model-choice accuracy, and when model violations do cause them to be biased, they tend to under-estimate the probability of the model with a single, shared divergence event. In contrast, the *M*_*msBayes*_ model is very sensitive to model violations, and strongly over-estimates the probability of the one-divergence model whenever the model is misspecified. Furthermore, the results suggest that using exponentially distributed priors on nuisance parameters rather than uniform priors helps the *M*_*Ushaped*_ model perform better than *M*_*msBayes*_, but it is still hindered by the U-shaped prior on divergence models and tends to over-estimate the probability of the one-divergence model whenever there are violations of the model.

### Validation analyses: Ordered divergence models

The results show that the method performs similarly when sampling over ordered models of divergence (Additional file 1: Figures S9 and S10). This suggests that the method is not adversely affected by the increase in the number of possible discrete models (from 22 unordered to 4140 ordered models) when there are eight pairs of populations. This is encouraging, because, as discussed above, estimating unordered models of divergence by shuffling the summary statistic vectors calculated from the sequence alignments is not valid for most empirical datasets. Given these results, estimation of unordered divergence models should be avoided for empirical applications of the method.

### Power analyses: Estimation accuracy

All of the models I evaluated (Table 2) struggle to estimate the variance of divergence times *D*_*T*_ regardless of which of the three series of models (Table 3) the data were generated under (Additional file 1: Figures S11–13). The models with the U-shaped prior on divergence models (*M*_*msBayes*_ and *M*_*Ushaped*_) tend to underestimate the variance in divergence times (Plots A–L of Additional file 1: Figures S11–13). whereas the models with Uniform or Dirichlet-process priors over divergence models tend to overestimate variance in divergence times (Plots M–X of Additional file 1: Figures S11–13).

When the divergence times of the 22 population pairs are randomly drawn from a series of exponential priors (${ℳ}_{\text{Exp}}$), the *M*_*DPP*_ model is the best estimator of *D*_*T*_, followed by *M*_*Uniform*_ (Additional file 1: Figure S11). The *M*_*msBayes*_ model is strongly biased toward underestimating *D*_*T*_, estimating values of zero for most of the replicates across all the data models of ${ℳ}_{\text{Exp}}$ (Additional file 1: Figure S11). The results of the *M*_*Ushaped*_ model are intermediate between those of *M*_*msBayes*_ and the new models *M*_*DPP*_ and *M*_*Uniform*_ (Additional file 1: Figure S11).

Similarly, when the true divergence times are randomly drawn from a series of uniform priors (${ℳ}_{\text{Uniform}}$), the *M*_*DPP*_ and *M*_*Uniform*_ models tend to over-estimate the variance in divergence times, whereas the *M*_*msBayes*_ model underestimates *D*_*T*_, estimating values of zero for most replicates across all the data models of ${ℳ}_{\text{Uniform}}$ (Additional file 1: Figure S12). Again, the performance of the *M*_*Ushaped*_ model is intermediate between the *M*_*msBayes*_ and *M*_*DPP*_/ *M*_*Uniform*_ models (Additional file 1: Figure S12). The results are very similar when the four models are applied to the data simulated under the ${ℳ}_{\text{msBayes}}$ series of models (Additional file 1: Figure S13).

### Power analyses: Model choice

**Figure 3:**
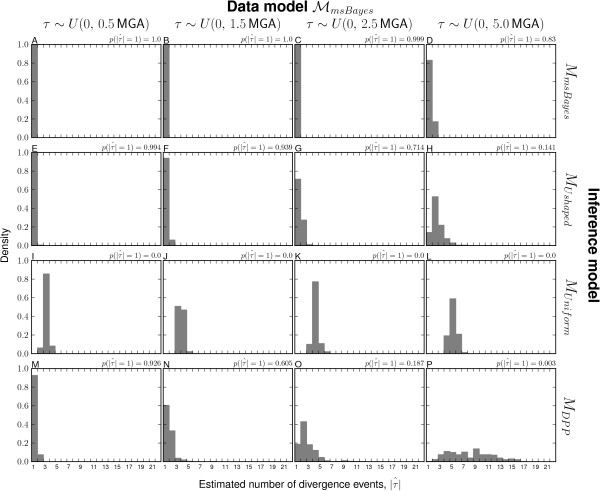
**Power to avoid spurious estimation of clustered divergences when divergence times are random.** The power of models **(A–D)***M*_*msBayes*_, **(E–H)***M*_*Ushaped*_, **(I–L)***M*_*Uniform*_, and **(M–P)***M*_*DPP*_ to detect random variation in divergence times as simulated under the ${ℳ}_{\text{msBayes}}$ series of models. The plots illustrate the estimated number of divergence events ($\left|\hat{\tau }\right|$) from analyses of 1000 datasets simulated under each of the ${ℳ}_{\text{msBayes}}$ models, with the the estimated probability of the model inferring one divergence event, $p(|\hat{\tau }|=1)$, given for each combination. The 22 divergence times were randomly drawn as indicated above each column of plots, where time is respresented as millions of generations ago (MGA) according to a per-site rate of 1 × 10^−8^ mutations per generation. Four of the six data-generating models of the ${ℳ}_{\text{msBayes}}$ series are shown; please see Additional file 1: Figure S14 for all results.

**Figure 4:**
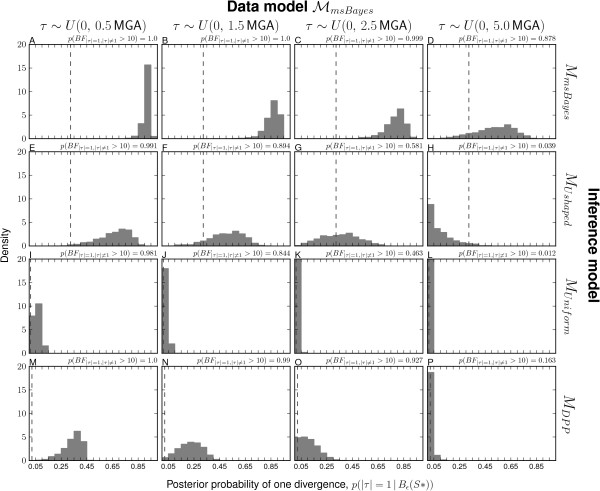
**Power to avoid spurious support for one divergence event when divergence times are random.** The tendency of models **(A–D)***M*_*msBayes*_, **(E–H)***M*_*Ushaped*_, **(I–L)***M*_*Uniform*_, and **(M–P)***M*_*DPP*_ to support one divergence event when there is random variation in divergence times as simulated under the ${ℳ}_{\text{msBayes}}$ series of models. The plots illustrate histograms of the estimated posterior probability of the one divergence model, *p*(|***τ***|=1|*B*_*ε*_(**S**^∗^)), from analyses of 1000 datasets simulated under each of the ${ℳ}_{\text{msBayes}}$ models. The 22 divergence times were randomly drawn as indicated above each column of plots, where time is respresented as millions of generations ago (MGA) according to a per-site rate of 1 × 10^−8^ mutations per generation. Four of the six data-generating models of the ${ℳ}_{\text{msBayes}}$ series are shown; please see Additional file 1: Figure S17 for all results.

The modifications of the msBayes model decrease the method’s bias toward clustered divergences when applied to data generated under random divergence times (Figure 3 and Additional file 1: Figures S14–16). The *M*_*msBayes*_ model performs the worst of the four models across all three series of data-generating models, inferring a single divergence event across most of the 18,000 simulations (Figure 3A–D and plots A–F of Additional file 1: Figures S14–16). Importantly, the *M*_*msBayes*_ model tends to strongly support these estimates of one divergence across most of the simulations (Figure 4A–D and plots A–F of Additional file 1: Figures S17–19). The *M*_*DPP*_ model also prefers the one-divergence model when divergences are random over narrow windows of time, but performs much better when divergences are random over a timescale of 1–2 coalescent units (Figure 3M–P and plots S–X of Additional file 1: Figures S14–16). However, even when *M*_*DPP*_ infers the one-divergence model over narrow timescales, the posterior probability support is always low (Figure 4M–P and plots S–X of Additional file 1: Figures S17–19). The *M*_*Uniform*_ model never infers the one-divergence model in any of the simulation replicates but still tends to infer relatively few (4–6) divergence events when divergences are random over longer periods (Figure 3I–L and plots M–R of Additional file 1: Figures S14–16). Using exponential priors on divergence-time and demographic parameters does increase the power of the *M*_*Ushaped*_ model compared to *M*_*msBayes*_ across all three series of data models, but the U-shaped prior still prevents the model from performing as well as the *M*_*DPP*_ and *M*_*Uniform*_ models (Figure 3 and Additional file 1: Figures S14–16).

**Figure 5:**
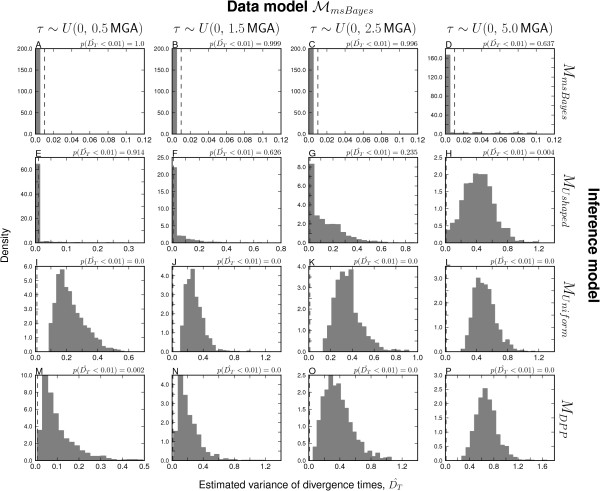
**Power to avoid spurious estimation of small temporal variance in divergences when divergence times are random.** The power of models **(A–D)***M*_*msBayes*_, **(E–H)***M*_*Ushaped*_, **(I–L)***M*_*Uniform*_, and **(M–P)***M*_*DPP*_ to detect random variation in divergence times as simulated under the ${ℳ}_{\text{msBayes}}$ series of models. The plots illustrate the estimated dispersion index of divergence times ($\left|\hat{\tau }\right|$) from analyses of 1000 datasets simulated under each of the ${ℳ}_{\text{msBayes}}$ models, with the the estimated probability of the model inferring one divergence event, $p(|\hat{\tau }|<0.01)$, given for each combination. The 22 divergence times were randomly drawn as indicated above each column of plots, where time is respresented as millions of generations ago (MGA) according to a per-site rate of 1 × 10^−8^ mutations per generation. Four of the six data-generating models of the ${ℳ}_{\text{msBayes}}$ series are shown; please see Additional file 1: Figure S20 for all results.

The improved power of the new models is even more pronounced when looking at estimates of the variance of divergence times (*D*_*T*_) across the simulations (Figure 5 and Additional file 1: Figures S20–22). The performance among the models is so different, that the histograms of *D*_*T*_ estimates cannot be plotted along a shared x-axis. The *M*_*DPP*_ and *M*_*Uniform*_ models perform similarly across all three series of data models, inferring values of *D*_*T*_ consistent with one divergence event (*D*_*T*_<0.01) in almost none of the replicates across all the simulations. In contrast, the *M*_*msBayes*_ model infers values consistent with a single divergence event in most of the replicates across all the simulations. Using exponential priors on divergence-time and demographic parameters greatly increases the power of the *M*_*Ushaped*_ model to detect variation in divergence times relative to *M*_*msBayes*_, but it still has less power than the models with Dirichlet-process or uniform priors across divergence models (Figure 5 and Additional file 1: Figure S20–22). Although the *D*_*T*_ threshold of 0.01 is arbitrary, Oaks et al. [7] did show via simulation that the true value of *D*_*T*_ will almost always be greater than 0.01 when divergences are random over periods of 0.1 coalescent units or more (see Figure Sfour of [7]).

As mentioned above, the increased power of the new models is also evident when looking at the estimated posterior probability of the one-divergence model across the power analyses (Figure 4 and Additional file 1: Figures S17–19). The *M*_*DPP*_ and *M*_*Uniform*_ models estimate low posterior probability of |***τ***|=1 across all of the simulations. This is in contrast to the *M*_*msBayes*_ model, which infers high posterior probabilities of a single divergence for most replicates across all simulations (Figure 4 and Additional file 1: Figures S17–19). The exponential priors on divergence-time and demographic parameters (model *M*_*Ushaped*_) result in lower estimates of the probability of one divergence when compared to *M*_*msBayes*_, but higher estimates when compared to *M*_*Uniform*_ and *M*_*DPP*_ (Figure 4 and Additional file 1: Figures S17–19). The *M*_*DPP*_ and *M*_*Uniform*_ models do frequently support the one-divergence model according to a Bayes factor criterion of greater than 10, but still less frequently than the *M*_*msBayes*_ model. This result is not surprising given the extremely small prior probability of the one-divergence model under the *M*_*DPP*_ and *M*_*Uniform*_ models (i.e., very few posterior samples of the one-divergence model will result in a large Bayes factor under these models). However, the small posterior probability of the one-divergence model estimated under *M*_*DPP*_ and *M*_*Uniform*_ should prevent an investigator from overinterpreting the Bayes factor as strong support for clustered divergences.

**Figure 6:**
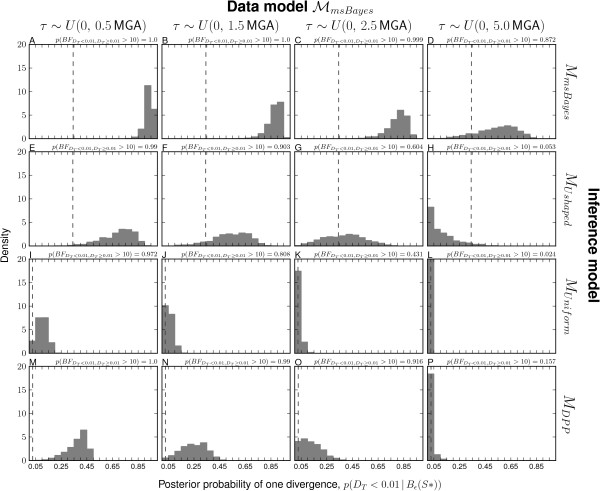
**Power to avoid spurious support for no temporal variance in divergences (i.e.,*D***_***T***_***<0.01*) when divergence times are random.** The tendency of models **(A–D)***M*_*msBayes*_, **(E–H)***M*_*Ushaped*_, **(I–L)***M*_*Uniform*_, and **(M–P)***M*_*DPP*_ to support one divergence event when there is random variation in divergence times as simulated under the ${ℳ}_{\text{msBayes}}$ series of models. The plots illustrate histograms of the estimated posterior probability of the one divergence model, *p*(*D*_*T*_<0.01|*B*_*ε*_(**S**^∗^)), from analyses of 1000 datasets simulated under each of the ${ℳ}_{\text{msBayes}}$ models. The 22 divergence times were randomly drawn as indicated above each column of plots, where time is respresented as millions of generations ago (MGA) according to a per-site rate of 1 × 10^−8^ mutations per generation. Four of the six data-generating models of the ${ℳ}_{\text{msBayes}}$ series are shown; please see Additional file 1: Figure S23 for all results.

Lastly, when looking at the estimated posterior probability of *D*_*T*_ being consistent with one shared divergence (*p*(*D*_*T*_<0.01|*B*_*ε*_(**S**^∗^))), I find the same pattern of model behavior, with *M*_*DPP*_ and *M*_*Uniform*_ inferring low probabilities across all simulations, *M*_*msBayes*_ inferring high probabilities, and *M*_*Ushaped*_ inferring intermediate values (Figure 6 and Additional file 1: Figures S23–25).

### Empirical results

**Figure 7:**
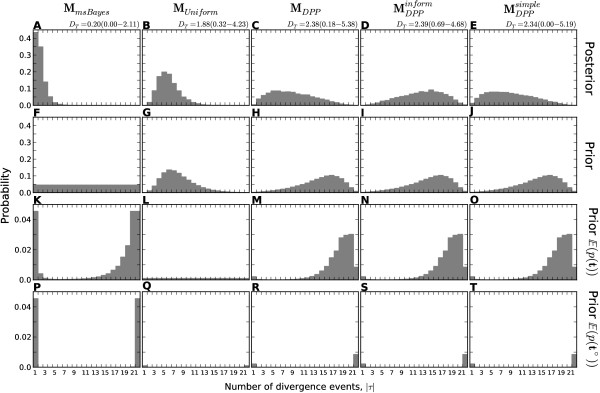
**Estimated number of divergence events for 22 taxa from the Philippines.** The **(A–E)** posterior and **(F–J)** prior probabilities of the number of divergence events (|***τ***|) when the data of the 22 pairs of taxa from the Philippines are analyzed under the five models indicated at the top of each column of plots (Table 4). The average prior probability of an **(K–O)** unordered and **(P–T)** ordered model of divergence (**t**) with |***τ***| divergence-time parameters is also shown. The posterior median of the dispersion index of divergence times (*D*_*T*_) is also given for each model, followed by the 95% highest posterior density interval in parentheses.

As expected based on the results of Oaks et al. [7], when the Philippines data are analyzed under the **M**_*msBayes*_ model, there is strong support for very few divergence events shared among all 22 pairs of taxa, with a maximum *a posteriori* (MAP) estimate of one-shared divergence (Figure 7A). When these data are analyzed using models allowed by the new implementation, there is much less support for highly clustered models and much greater uncertainty regarding the number of divergence events shared among the taxa, especially under the DPP models (Figure 7B–E). Figure 7 also shows the prior distribution across the number of divergence events (|***τ***|) for each model, as well as the average prior probability of an unordered and ordered model of divergence (***t***) across |***τ***|. Estimates under the new models tend to be similar to the prior, which is expected under such a parameter-rich model when there is limited information from the data (four summary statistics from a single locus for each pair of taxa).

The disparity between the results of the **M**_*msBayes*_ model and the new models is even more pronounced when looking at the 10 divergence models (**t**) estimated to have the highest probability under each of the models (Additional file 1: Figures S26–30). Again, the new models estimate more divergences, a large amount of posterior uncertainty, and an order of magnitude smaller probability for their respective MAP-divergence model when compared to the **M**_*msBayes*_ model (Additional file 1: Figures S26–30).

**Figure 8:**
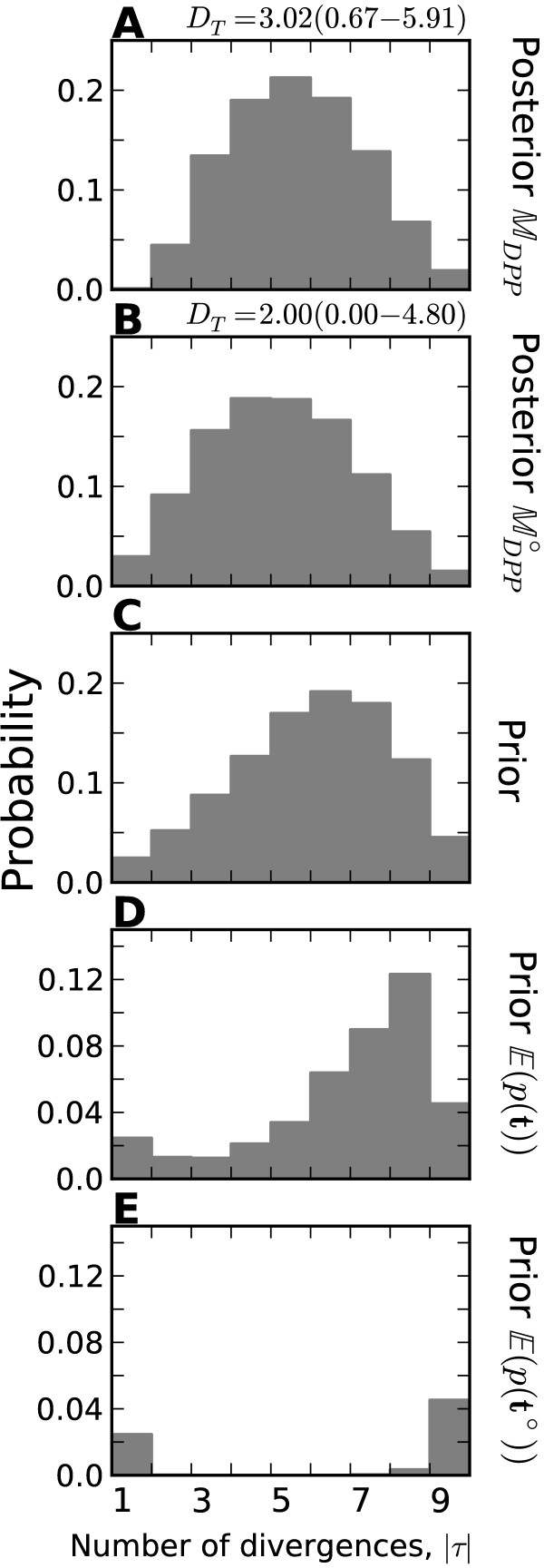
**Estimated number of divergence events for 9 taxa from the Philippines.** The posterior probabilities of the number of divergence events, |***τ***|, when the data of the 9 pairs of taxa from Negros and Panay Islands are analyzed under the DPP model that samples over **(A)** unordered and **(B)** ordered models of divergence (Table 4). Both models share the same **(C)** prior probability of the number of divergence events, and the average prior probability of an **(D)** unordered and **(E)** ordered model of divergence (**t**) with |***τ***| divergence-time parameters. The posterior median of the dispersion index of divergence times (*D*_*T*_) is also given for each model, followed by the 95% highest posterior density interval in parentheses.

Figure 8 shows the estimated posterior probability distribution over the number of divergence events when the data from the nine-taxon pairs from the Islands of Negros and Panay are analyzed under DPP models that sample over unordered ($M_{\text{DPP}}$) and ordered ($M{\text{°}}_{\text{DPP}}$) models of divergence. The results are similar under both models and, again, yield a large amount of uncertainty about the number of divergence events that is similar to the prior uncertainty.

The small difference between the results of the $M_{\text{DPP}}$ and $M{\text{°}}_{\text{DPP}}$ models is consistent across multiple analyses, and thus could be due to error introduced to the $M_{\text{DPP}}$ model by the invalid shuffling of the summary statistic vectors. Both models estimate a similar set of 10 unordered divergence models with the highest posterior probability (Additional file 1: Figures S31 and S32).

The main advantages of the $M{\text{°}}_{\text{DPP}}$ model over the $M_{\text{DPP}}$ are that (1) the incorrect shuffling of the summary statistic vectors is avoided, (2) the identity of the taxa is maintained, and thus a fully marginalized estimate of divergence times across the taxa can be obtained (Additional file 1: Figure S33), and (3) the probability of co-divergence among any set of taxa can be estimated from the posterior sample.

## Discussion

My results demonstrate that using alternative priors on parameters and divergence models improved the behavior of the msBayes model. In the new implementation, model-choice estimation is more accurate and shows greater robustness to model violations (Figures 1 and 2). The original model is very sensitive to violations and, when present, strongly over-estimates the probability of one-divergence event shared across all taxa (Figures 1 and 2). When more appropriate priors are used for divergence-time and demographic parameters, and either a Dirichlet-process or uniform prior applied across divergence models, the model is less sensitive to violations, and, when violations do cause bias, the method tends to underestimate the probability of models with temporally clustered divergences (Figures 1 and 2). Given that clustered models are often of particular interest to biogeographers, this behavior of the new method can be considered conservative.

The modifications also improve the method’s power to detect random variation in divergence times, reducing the tendency to estimate clustered divergences (Figures 3, 4, 5 and 6). My results are similar to those of Oaks et al. [7] in that I find msBayes will often infer strong support for clustered divergences when divergences are random over quite broad timescales (Figures 3, 4, 5 and 6). My results expand on this by showing that this behavior is consistent across a range of conditions underlying the data. The new method, dpp-msBayes, has greater power to detect random temporal variation in divergences, is less prone to spurious inference of clustered divergence models, and much less likely to incorrectly infer such models with strong support (Figures 3, 4, 5 and 6).

By evaluating a model intermediate between the old and new implementation (*M*_*Ushaped*_), I was able to determine the relative affects of my modifications to the model. Across all of the analyses, the results show that using better priors on divergence-time and demographic parameters alone does improve the performance of the method. The magnitude of the bias toward inferring support for the one-divergence model when there are model violations is reduced when the exponential priors are used in place of the uniform priors (Figures 1 and 2). Furthermore, using exponential priors improves the method’s power to detect temporally random divergences (Figures 3, 4, 5 and 6). Throughout the analyses, the intermediate model (*M*_*Ushaped*_) performs better than the msBayes model, but not as well as the models with alternative priors on divergence models. This suggests, as predicted by Oaks et al. [7,15], that the tendency of msBayes to erroneously support models of temporally clustered divergences is caused by a combination of (1) small marginal likelihoods of models with more *τ* parameters due to uniform priors on divergence-time and demographic parameters and (2) the U-shaped prior on divergence models giving low prior density to models with intermediate numbers of divergence parameters. The former essentially rules out models with many *τ* parameters, which causes the latter to act like an "L-shaped" prior with a spike of prior density on the one-divergence model. Given the parameter richness of the model and the relatively small amount of information in the summary statistics, it is not surprising that the combination of these two factors can create a strong tendency to infer clustered models of divergence.

While the modifications improve the behavior of the model, I urge caution when using the method and interpreting its results. The method attempts to approximate the posterior of a very parameter-rich model using relatively little information from the data. For example, when applied to the dataset of 22 taxon pairs from the Philippines [7], the model has as many as 604–625 free parameters (depending on |***τ***|), and samples over 1002 unordered divergence models. Even under the simplest possible model allowed under the new implementation, the model still has 471–492 free parameters. Furthermore, the stochastic coalescent and mutational processes being modeled predict a large amount of variation in possible datasets even when the parameter values are known. The richness and stochastic nature of the model makes for a difficult inference problem, especially when using a small number of summary statistics calculated from the sequence alignments of each taxon pair. The population-genetic summary statistics used by the method contain little information about many of the free parameters in the model. Thus, I expect the improved method will still be sensitive to priors, and the power, while improved, may still be low. While there is much less prior sensitivity under the new model compared to those observed by Oaks et al. [7], there is still an effect when comparing the results of the empirical data analyzed under a diffuse (**M**_*DPP*_) and informative $\left(M_{\text{DPP}}^{\text{inform}}\right)$ divergence-time prior (Figure 7C versus D). The fact that the posterior shifts toward the prior under the informative prior suggests that the shift away from the prior toward fewer divergence events under the diffuse prior might still be caused by small marginal likelihoods of models with more divergence-time parameters (Figure 7).

Nonetheless, it is reassuring to see a large amount of posterior uncertainty when the new implementation is applied to the empirical datasets (Figures 7 and 8). Applications of the msBayes model often result in strong posterior support for estimated scenarios (e.g., [3,5-12]), as I found here (Figure 7). Given the richness of the model, the variance of the processes being modeled, and the relatively small amount of information in the summary statistics calculated from the sequence data, finding strong posterior support for any scenario is unexpected. Based on results of the empirical and power analyses (Figures 4, 6, 7 and 8), the new implementation more accurately reflects posterior uncertainty and avoids spurious support for biogeographical scenarios.

I also urge caution when using dpp-msBayes due to the lack of theoretical validation of Bayesian model choice when the full data are replaced by summary statistics that are insufficient for discriminating across models under comparison [44], which is certainly the case here. Robert et al. [44] demonstrated that ABC estimates of model posterior probabilities can be inaccurate when such across-model insufficient statistics are used.

Given all of these caveats, I encourage investigators to view this method as a means of exploring their data for general temporal patterns of divergences across taxa, rather than a rigorous means of evaluating hypotheses. As recommended by Oaks et al. [7], any results from the method should be accompanied by (1) analyses under a variety of priors to assess the assumptions underlying model inference and the prior sensitivity of the results, and (2) simulation-based power analyses to provide insight into the temporal resolution of the method. Both approaches are important to help guide the interpretation of results.

Given the difficulty of this estimation problem, I anticipate that full-likelihood methods that can leverage all of the information present in the sequence data will become increasingly important for robustly estimating shared evolutionary history across taxa [45]. With improving numerical methods for sampling over models of differing dimensionality [46,47], advances in Monte Carlo techniques [48], and increasing efficiency of likelihood calculations [49], analyzing rich comparative phylogeograpical models in a full-likelihood Bayesian framework is becoming computationally practical, especially when considering that simulating millions of random datasets from the prior under the simple ABC rejection approach is inefficient and computationally nontrivial.

## Conclusions

I introduced a new model for estimating shared divergence histories across taxa from DNA sequence data within an approximate-Bayesian model-choice framework. The new method, dpp-msBayes, takes a non-parametric approach to the problem by using a Dirichlet-process prior on the temporal distribution of divergences across taxa. The new method shows improved robustness, accuracy, and power compared to the existing method, msBayes. Compared to msBayes, the new approach better estimates posterior uncertainty, which greatly reduces the chances of incorrectly estimating biogeographical scenarios of shared divergence events. This is important, because models of shared divergence events are often ofparticular interest to researchers who employ these methods. This new tool will allow evolutionary biologists to better leverage comparative genetic data to assess the affects of regional and global biogeographical processes on biodiversity.

## Competing interests

The author declare that he has no competing interests.

## Authors’ contributions

All aspects of this work were done by JRO.

## Supplementary Material

Additional file 1 **Supporting table and figures.** PDF of supporting **Table S1** and **Figures S1-S33.** As referenced in the main text. Click here for file
