# Supplementary material for: An improved approximate-Bayesian model-choice method for estimating shared evolutionary history
Source: BMC Evol Biol. 2014 Jul 3;14:150. doi: 10.1186/1471-2148-14-150 (PMC4227068; doi:10.1186/1471-2148-14-150)
Supplement: Additional file 1 — Supporting table and figures. PDF of supporting Table S1 and Figures S1-S33. As referenced in the main text. [file 1471-2148-14-150-S1.pdf]

Supporting Information

Oaks, J. R. An Improved Approximate-Bayesian Model-choice Method for Estimating Shared Evolutionary History.

Table S1: An example showing the number of divergence events ($|\boldsymbol{\tau}|$) and the associated sample space of the unordered divergence models (integer partitions of Y pairs) and ordered divergence models (partitions of Y pairs) for $Y = 4$ pairs of populations.

$\lvert\boldsymbol{\tau}\rvert$	Unordered divergence models	Ordered divergence models
1	4	1111
2	$3 + 1$; $2 + 2$	1112, 1121, 1211, 2111, 1122, 1212, 1221
3	$2 + 1 + 1$	1123, 1213, 1231, 1223, 1232, 1233
4	$1 + 1 + 1 + 1$	1234

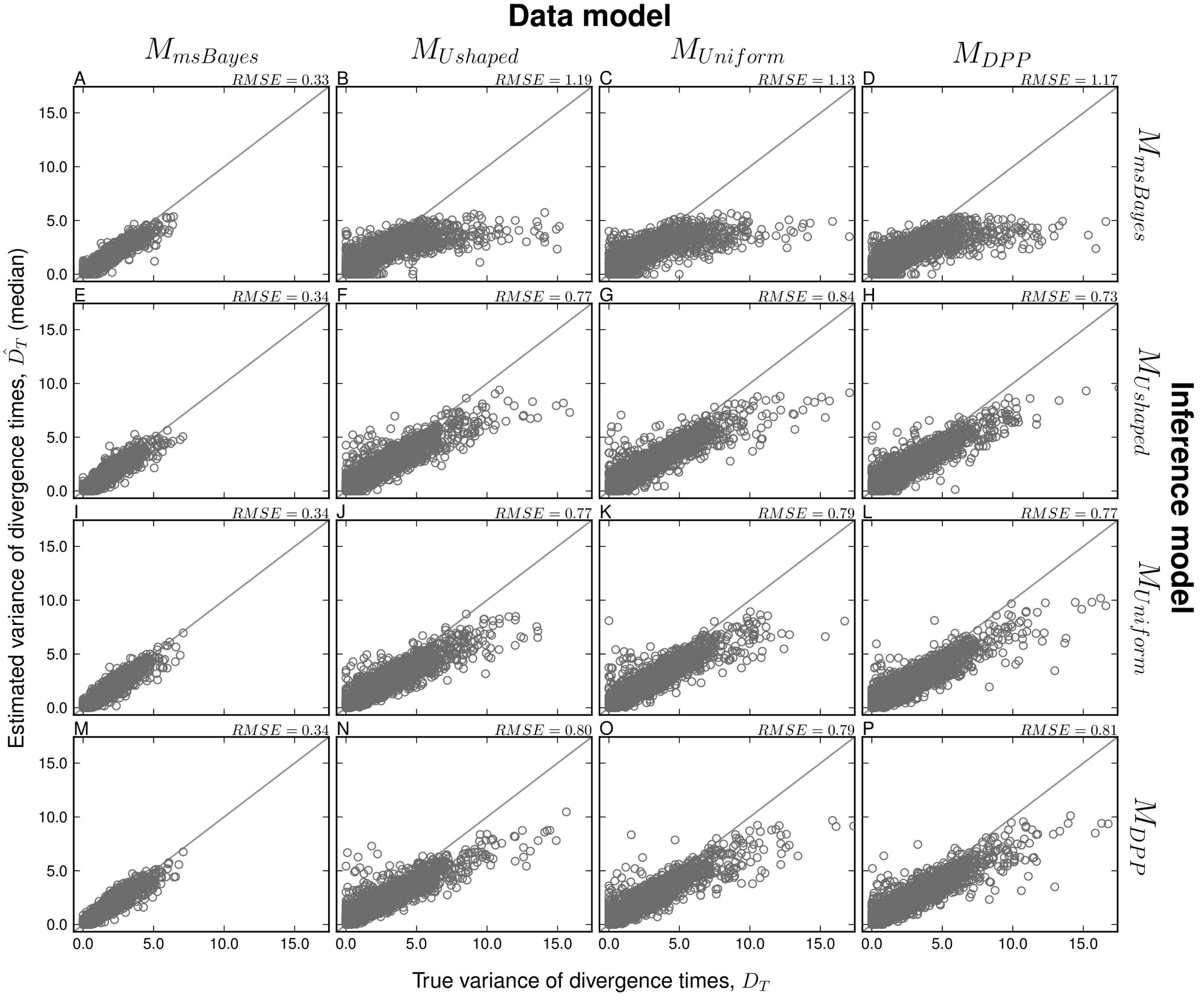

Figure S1: Accuracy of unadjusted estimates of D_T when data generated under models (A, E, I, & M) $M_{msBayes}$, (B, F, J, & N) $M_{Ushaped}$, (C, G, K, & O) $M_{Uniform}$, and (D, H, L, & P) M_{DPP} are analyzed with models (A–D) $M_{msBayes}$, (E–H) $M_{Ushaped}$, (I–L) $M_{Uniform}$, and (M–P) M_{DPP}. A random sample of 5000 posterior estimates (from 50,000) are plotted. The root mean square error (RMSE) calculated from the 5000 estimates is provided.

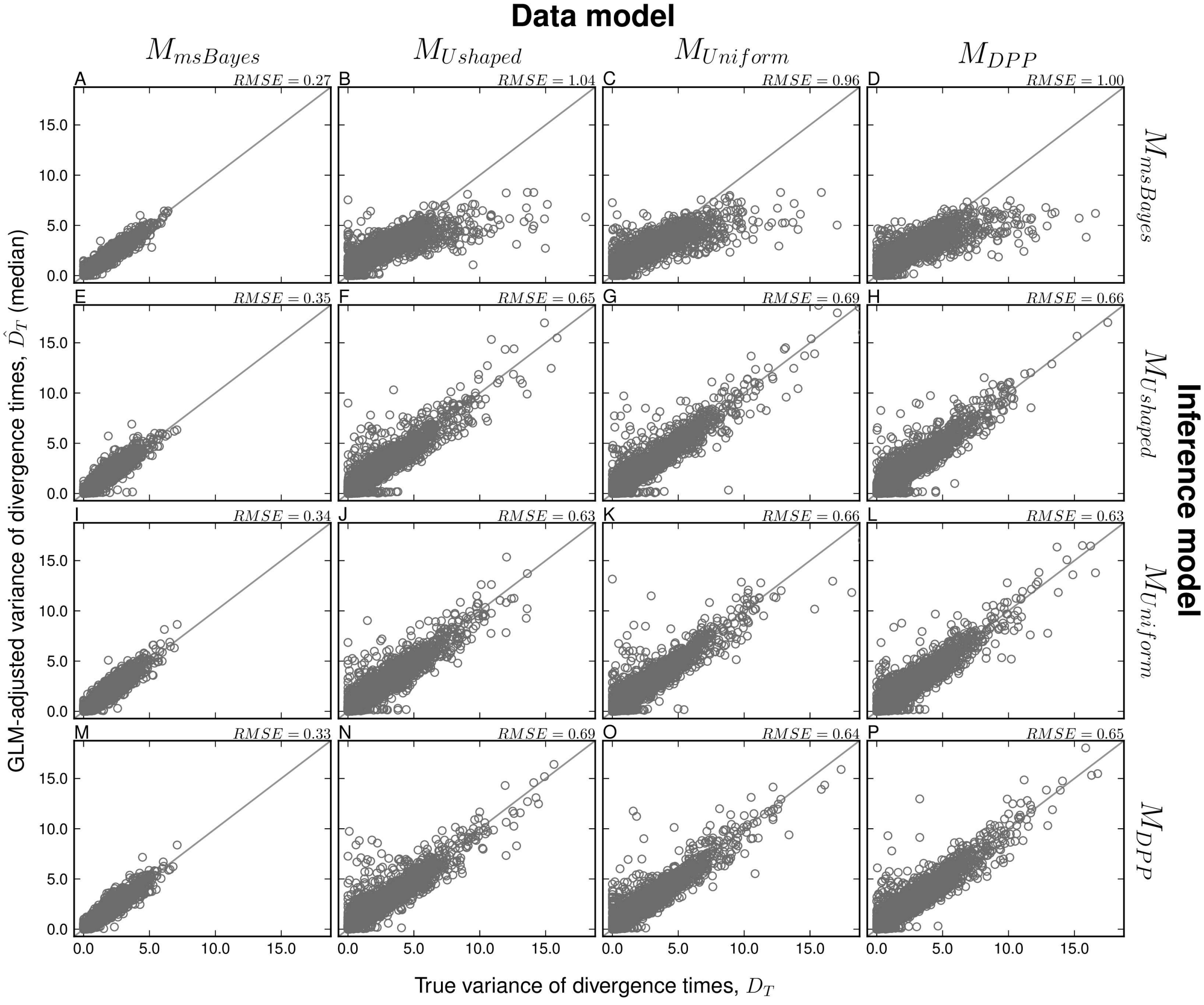

Figure S2: Accuracy of GLM-adjusted estimates of D_T when data generated under models (A, E, I, & M) $M_{msBayes}$, (B, F, J, & N) $M_{Ushaped}$, (C, G, K, & O) $M_{Uniform}$, and (D, H, L, & P) M_{DPP} are analyzed with models (A–D) $M_{msBayes}$, (E–H) $M_{Ushaped}$, (I–L) $M_{Uniform}$, and (M–P) M_{DPP}. A random sample of 5000 posterior estimates (from 50,000) are plotted. The root mean square error (RMSE) calculated from the 5000 estimates is provided.

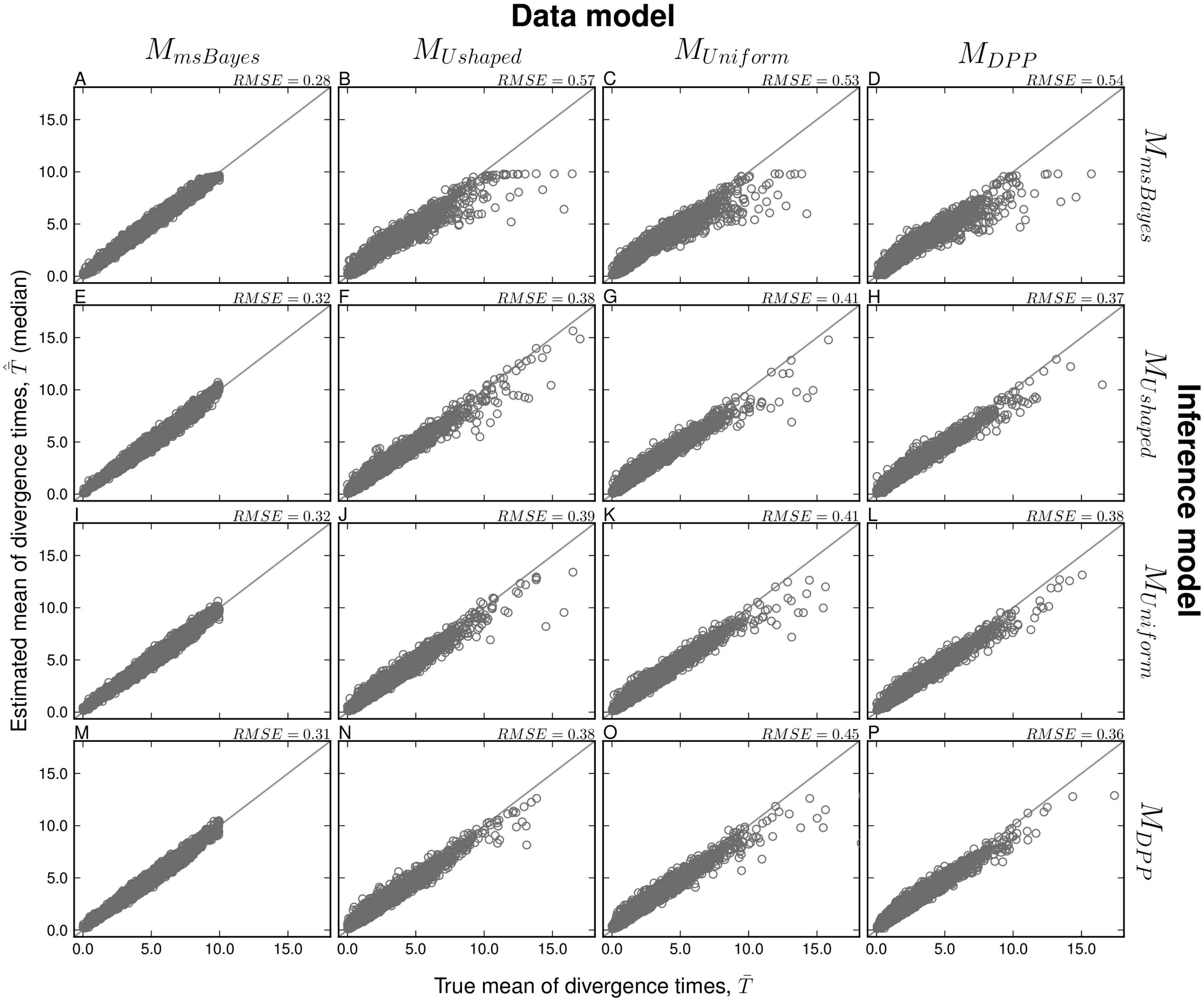

Figure S3: Accuracy of unadjusted estimates of $\bar{T}$ when data generated under models (A, E, I, & M) $M_{msBayes}$, (B, F, J, & N) $M_{Ushaped}$, (C, G, K, & O) $M_{Uniform}$, and (D, H, L, & P) M_{DPP} are analyzed with models (A–D) $M_{msBayes}$, (E–H) $M_{Ushaped}$, (I–L) $M_{Uniform}$, and (M–P) M_{DPP}. A random sample of 5000 posterior estimates (from 50,000) are plotted. The root mean square error (RMSE) calculated from the 5000 estimates is provided.

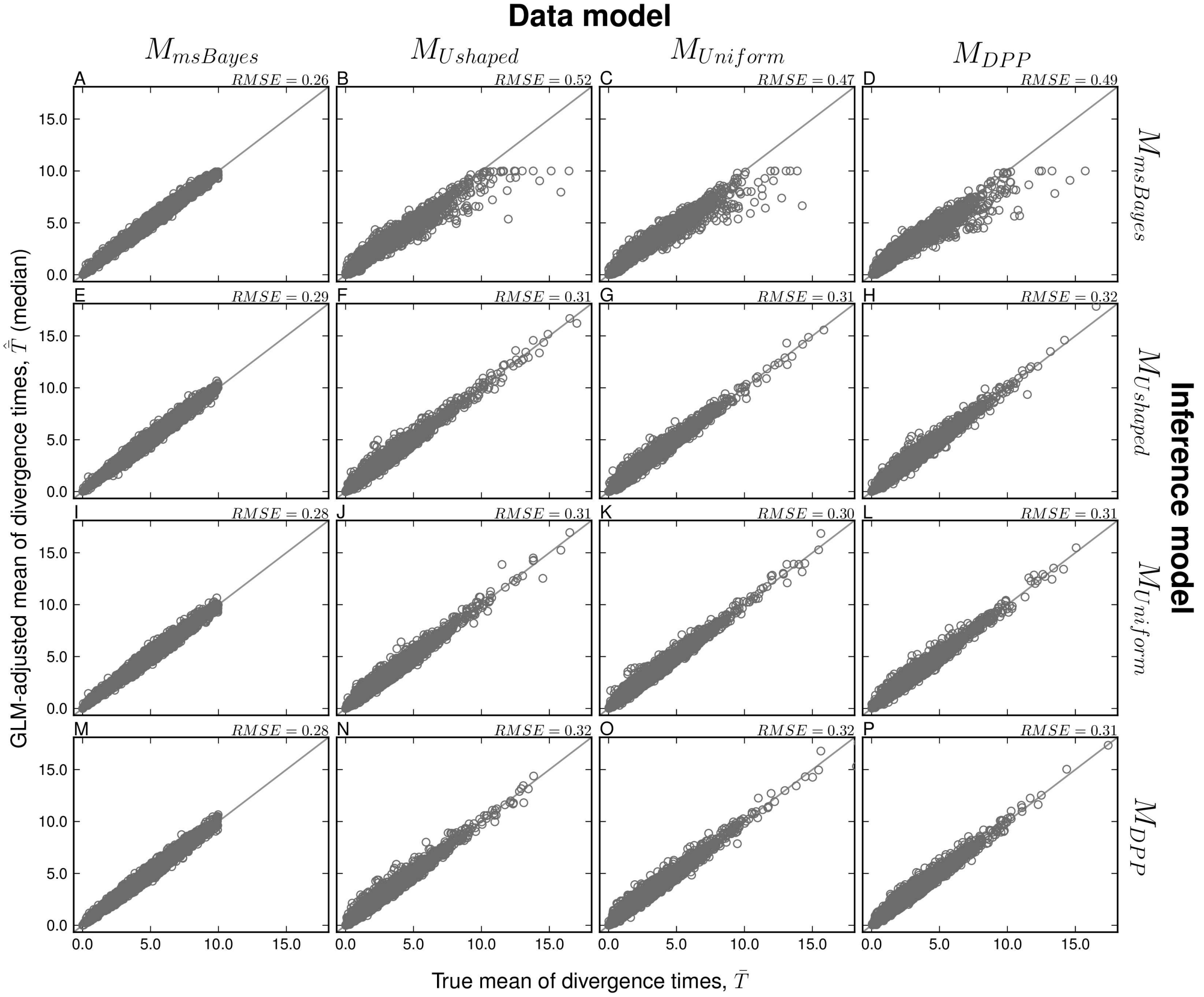

Figure S4: Accuracy of GLM-adjusted estimates of $\bar{T}$ when data generated under models (A, E, I, & M) $M_{msBayes}$, (B, F, J, & N) $M_{Ushaped}$, (C, G, K, & O) $M_{Uniform}$, and (D, H, L, & P) M_{DPP} are analyzed with models (A–D) $M_{msBayes}$, (E–H) $M_{Ushaped}$, (I–L) $M_{Uniform}$, and (M–P) M_{DPP}. A random sample of 5000 posterior estimates (from 50,000) are plotted. The root mean square error (RMSE) calculated from the 5000 estimates is provided.

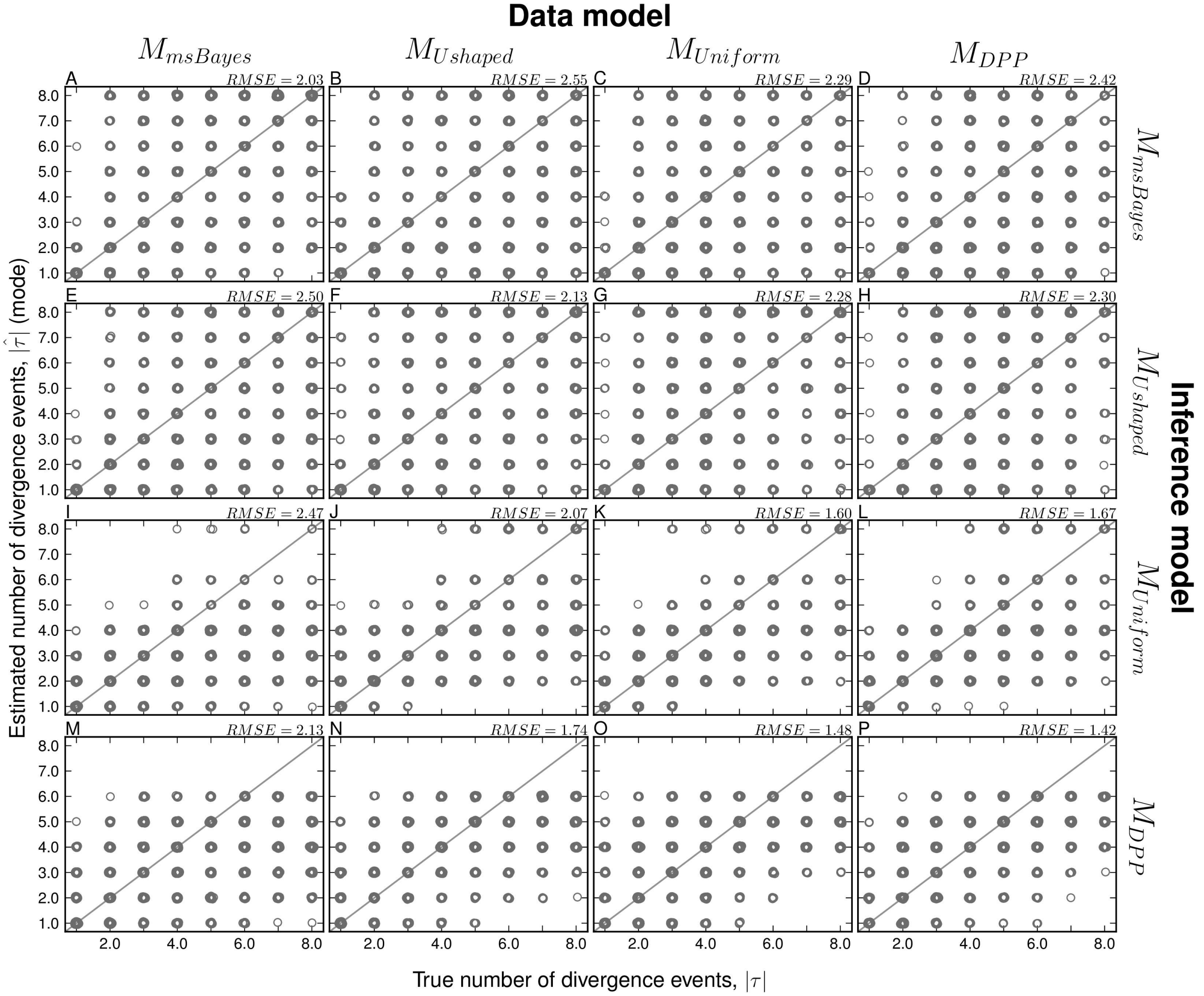

Figure S5: Accuracy of unadjusted estimates of $|\boldsymbol{\tau}|$ when data generated under models (A, E, I, & M) $M_{msBayes}$, (B, F, J, & N) $M_{Ushaped}$, (C, G, K, & O) $M_{Uniform}$, and (D, H, L, & P) M_{DPP} are analyzed with models (A–D) $M_{msBayes}$, (E–H) $M_{Ushaped}$, (I–L) $M_{Uniform}$, and (M–P) M_{DPP}. A random sample of 5000 posterior estimates (from 50,000) are plotted. The root mean square error (RMSE) calculated from the 5000 estimates is provided. Random normal variates ($N(0, 0.005)$) have been added to the estimates and true values of $|\boldsymbol{\tau}|$ to reduce overlap of plot symbols.

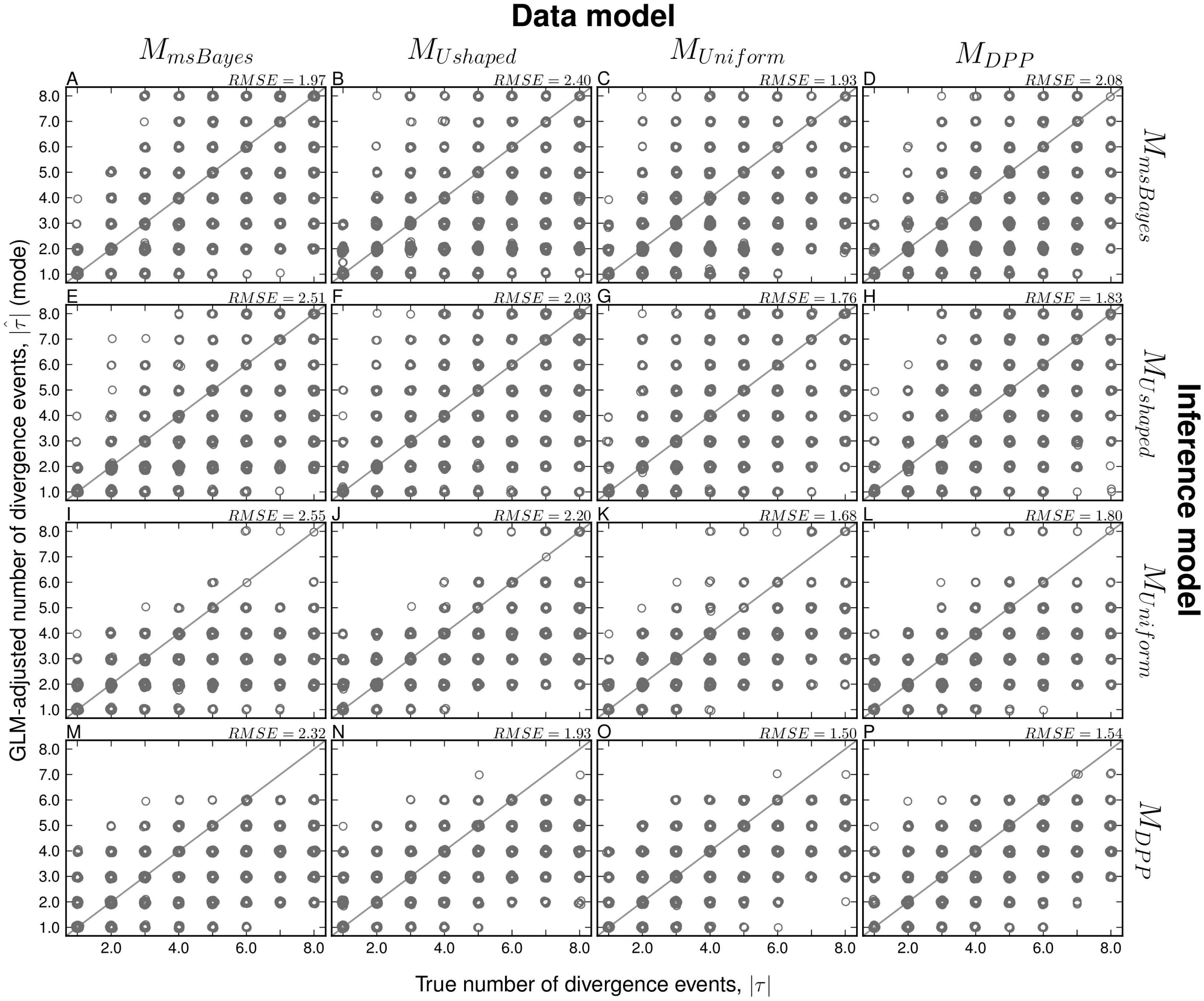

Figure S6: Accuracy of GLM-adjusted estimates of $|\boldsymbol{\tau}|$ when data generated under models (A, E, I, & M) $M_{msBayes}$, (B, F, J, & N) $M_{Ushaped}$, (C, G, K, & O) $M_{Uniform}$, and (D, H, L, & P) M_{DPP} are analyzed with models (A–D) $M_{msBayes}$, (E–H) $M_{Ushaped}$, (I–L) $M_{Uniform}$, and (M–P) M_{DPP}. A random sample of 5000 posterior estimates (from 50,000) are plotted. The root mean square error (RMSE) calculated from the 5000 estimates is provided. Random normal variates ($N(0, 0.005)$) have been added to the estimates and true values of $|\boldsymbol{\tau}|$ to reduce overlap of plot symbols.

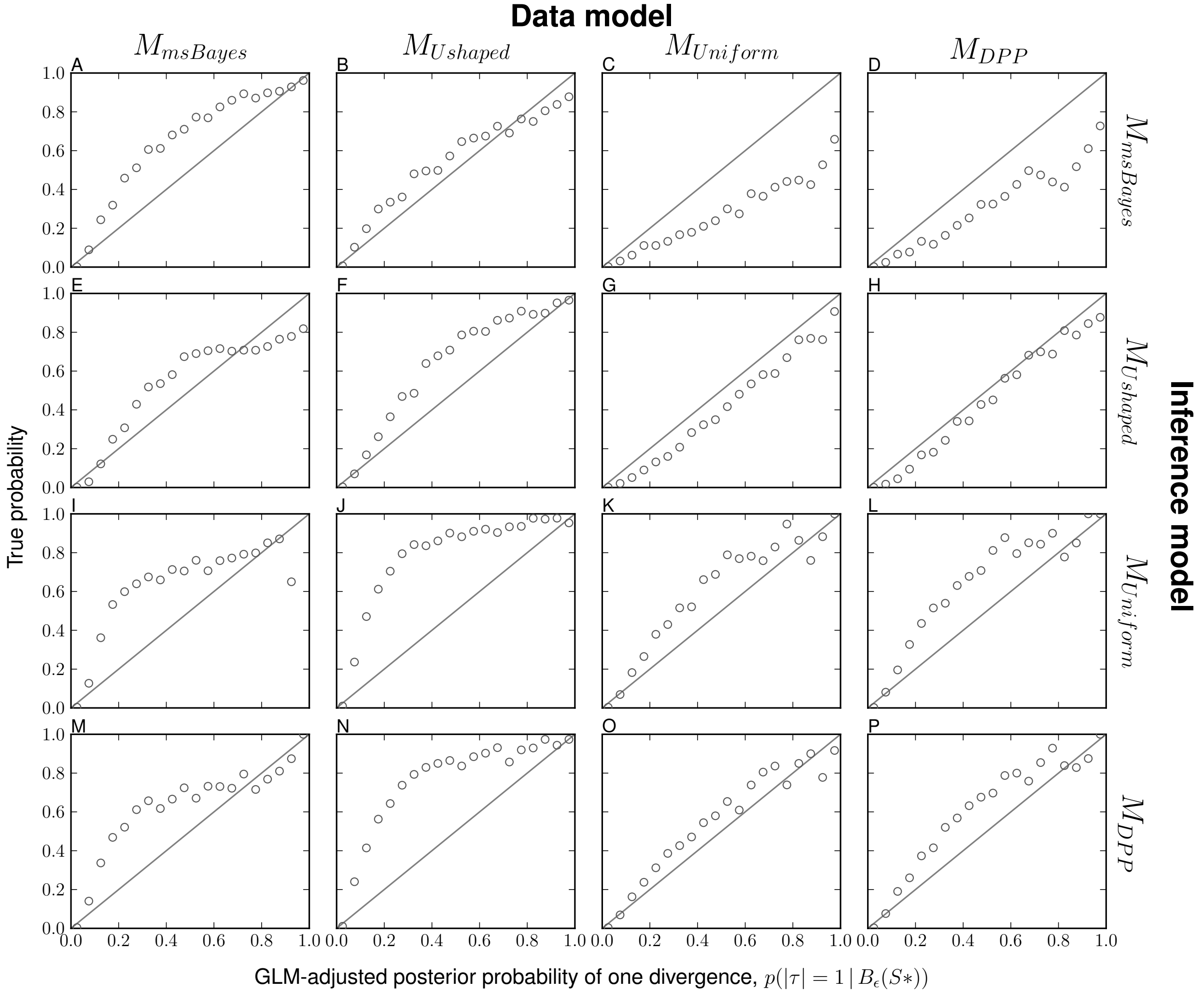

Figure S7: Model-choice accuracy for models (A–D) $M_{msBayes}$, (E–H) $M_{Ushaped}$, (I–L) $M_{Uniform}$, and (M–P) M_{DPP} when analyzing data generated under models (A, E, I, & M) $M_{msBayes}$, (B, F, J, & N) $M_{Ushaped}$, (C, G, K, & O) $M_{Uniform}$, and (D, H, L, & P) M_{DPP}. The GLM-adjusted posterior probability of a single divergence event, based on $|\boldsymbol{\tau}| = 1$, from 50,000 posterior estimates are assigned to bins of width 0.05 and plotted against the proportion of replicates in each bin where the truth is $|\boldsymbol{\tau}| = 1$.

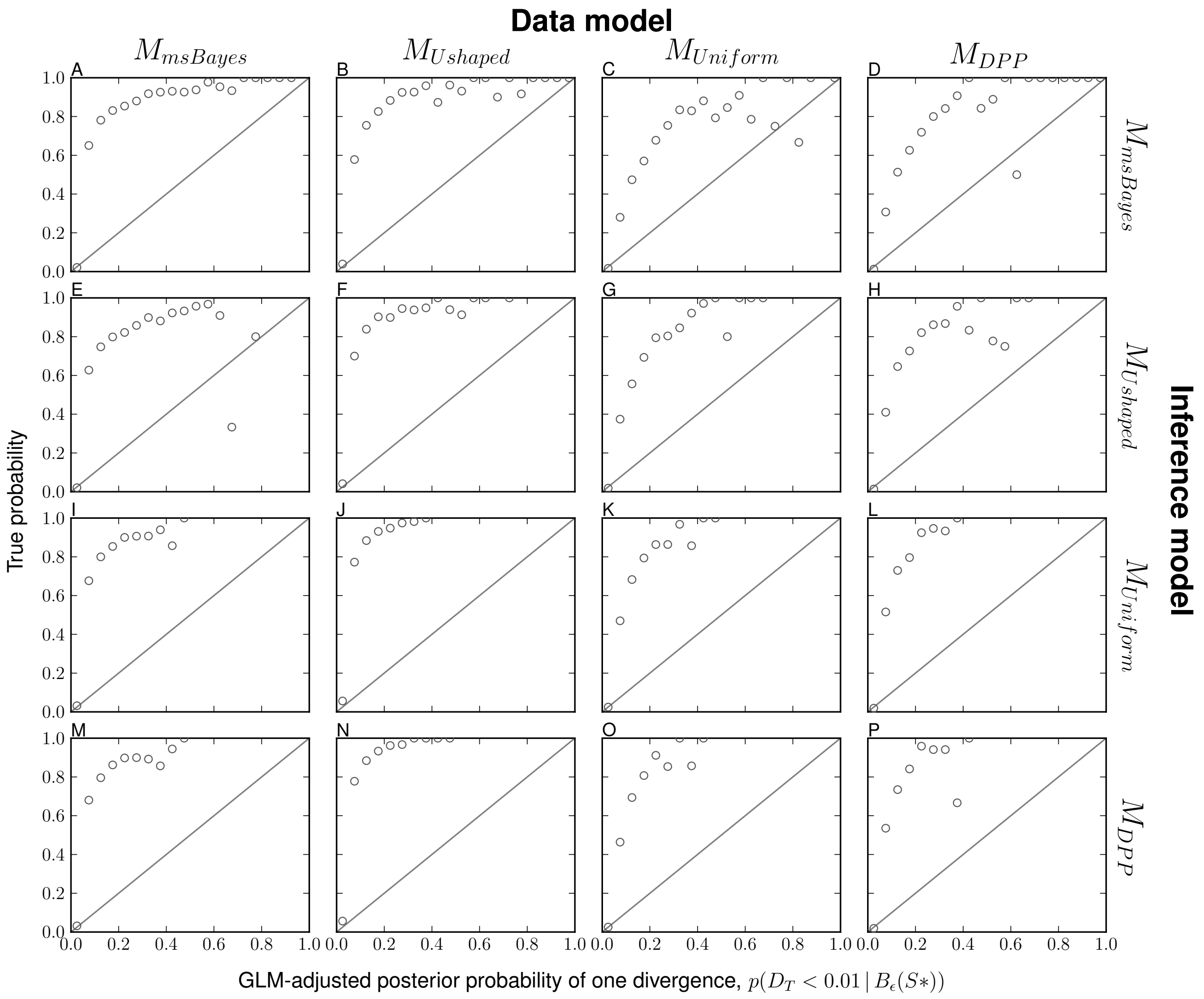

Figure S8: Model-choice accuracy for models (A–D) $M_{msBayes}$, (E–H) $M_{Ushaped}$, (I–L) $M_{Uniform}$, and (M–P) M_{DPP} when analyzing data generated under models (A, E, I, & M) $M_{msBayes}$, (B, F, J, & N) $M_{Ushaped}$, (C, G, K, & O) $M_{Uniform}$, and (D, H, L, & P) M_{DPP}. The GLM-adjusted posterior probability of a single divergence event, based on $D_T < 0.01$, from 50,000 posterior estimates are assigned to bins of width 0.05 and plotted against the proportion of replicates in each bin where the truth is $D_T < 0.01$.

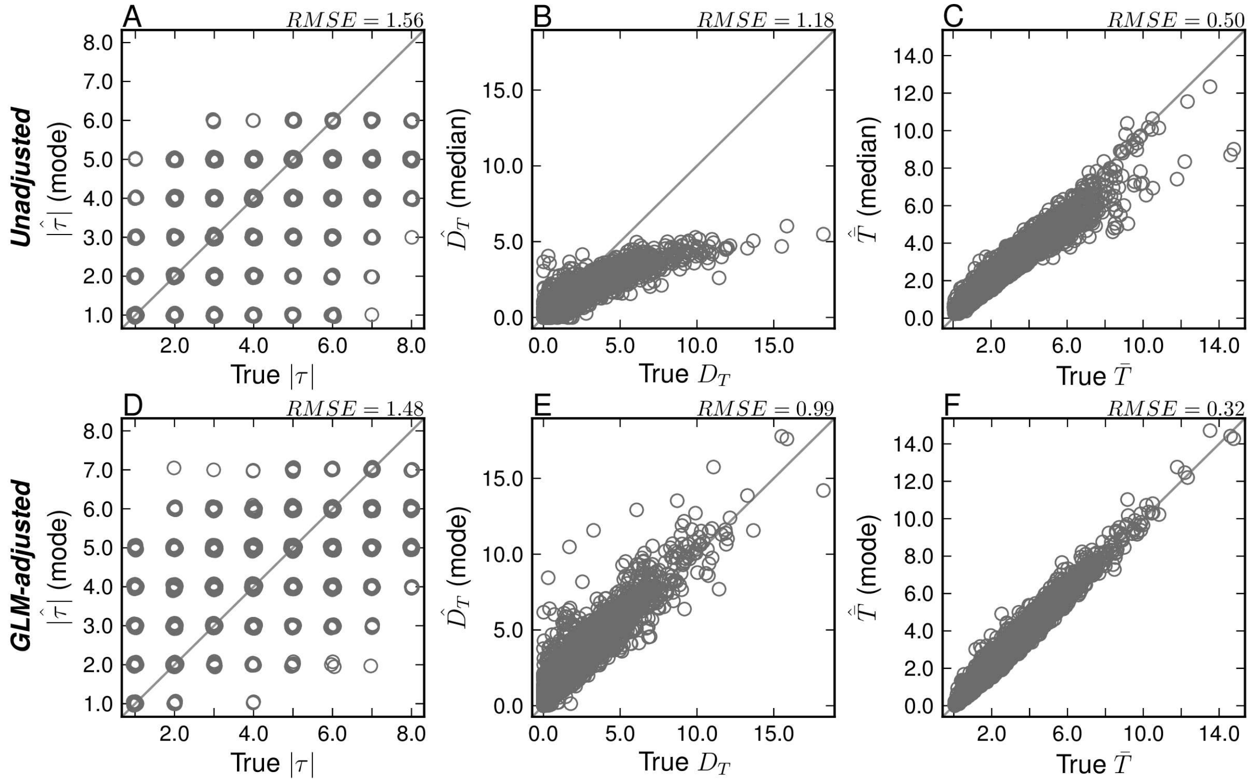

Figure S9: Estimation accuracy for model M°_{DPP} when analyzing data generated under M°_{DPP}. A random sample of 5000 posterior estimates (from 50,000) are plotted, including both (A, B, & C) unadjusted and (D, E, & F) GLM-regression-adjusted estimates. Normal random variates ($N(0, 0.005)$) have been added to the estimates and true values of $|\boldsymbol{\tau}|$ (A & D) to reduce overlap of plot symbols. The root mean square error (RMSE) calculated from the 5000 estimates is provided.

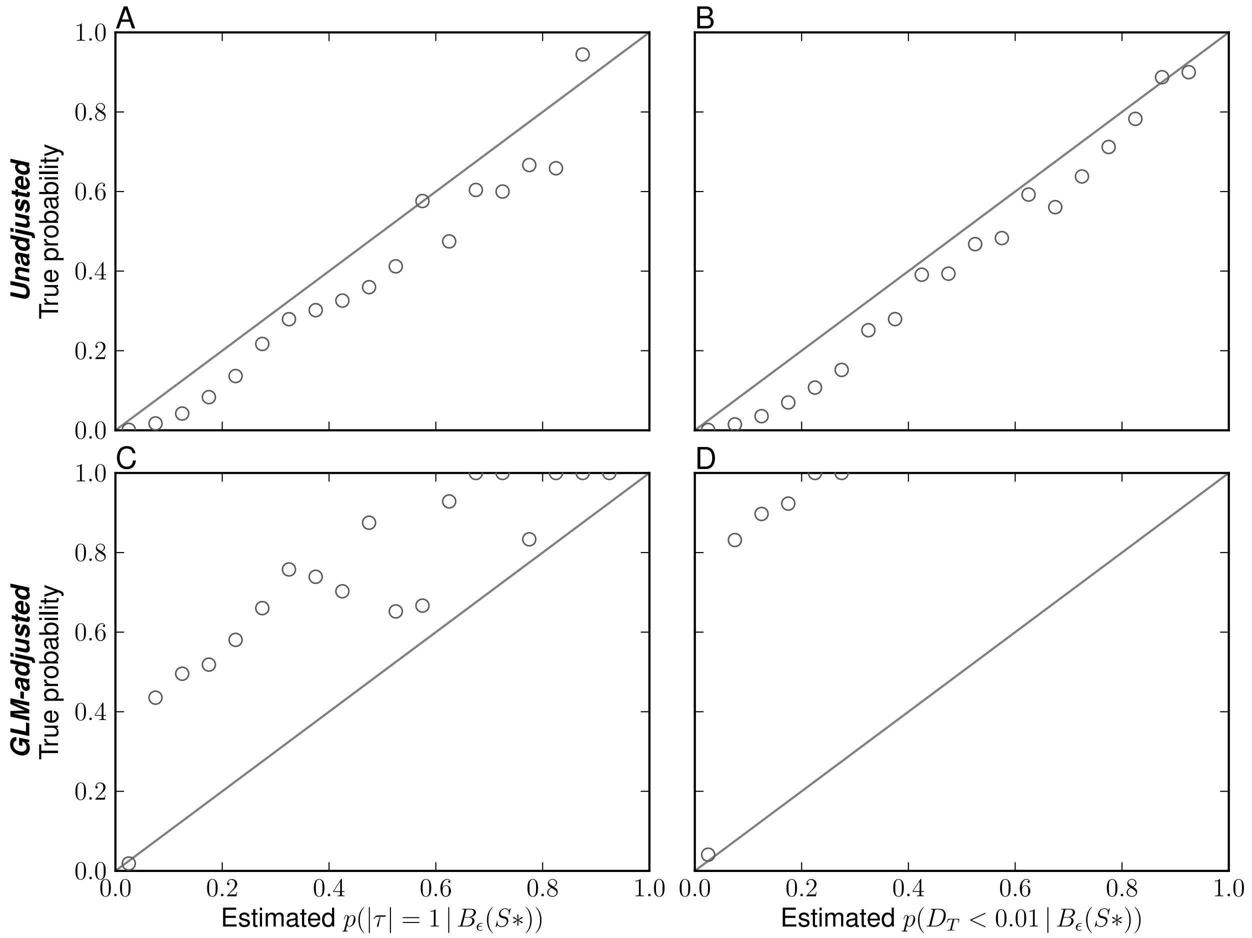

Figure S10: Model-choice accuracy for model M°_{DPP} when analyzing data generated under M°_{DPP}. The estimated posterior probability of a single divergence event, based on (A & C) $|\boldsymbol{\tau}| = 1$ and (B & D) $D_T < 0.01$, from 50,000 posterior estimates are assigned to bins of width 0.05 and plotted against the proportion of replicates in each bin where the truth is $|\boldsymbol{\tau}| = 1$ or $D_T < 0.01$. Results based on the (A & B) unadjusted and (C & D) GLM-adjusted posterior estimates are shown.

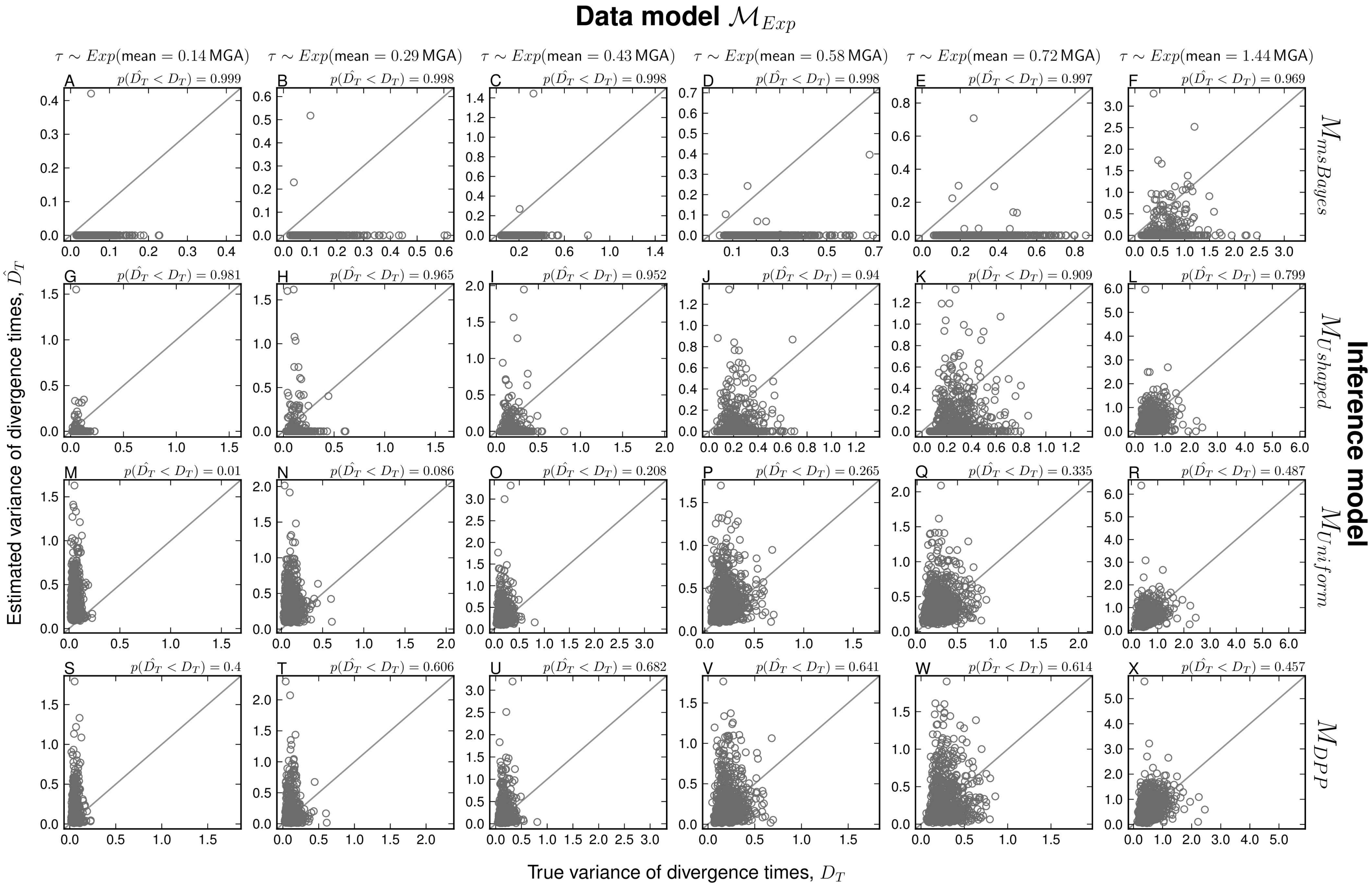

Figure S11: Accuracy of D_T estimates for models (A–F) $M_{msBayes}$, (G–L) $M_{Ushaped}$, (M–R) $M_{Uniform}$, and (S–X) M_{DPP} when analyzing data generated under the $\mathcal{M}_{Exp}$ series of models. The true versus estimated value of the dispersion index of divergence times (D_T, in $4N_C$ generations) is plotted for 1000 datasets simulated under each of the $\mathcal{M}_{Exp}$ models, and the proportion of estimates less than the truth, $p(\hat{D}_T < D_T)$, is shown for each data model. The 22 divergence times were randomly drawn as indicated above each column of plots, where time is respresented as millions of generations ago (MGA) according to a per-site rate of 1×10^{-8} mutations per generation.

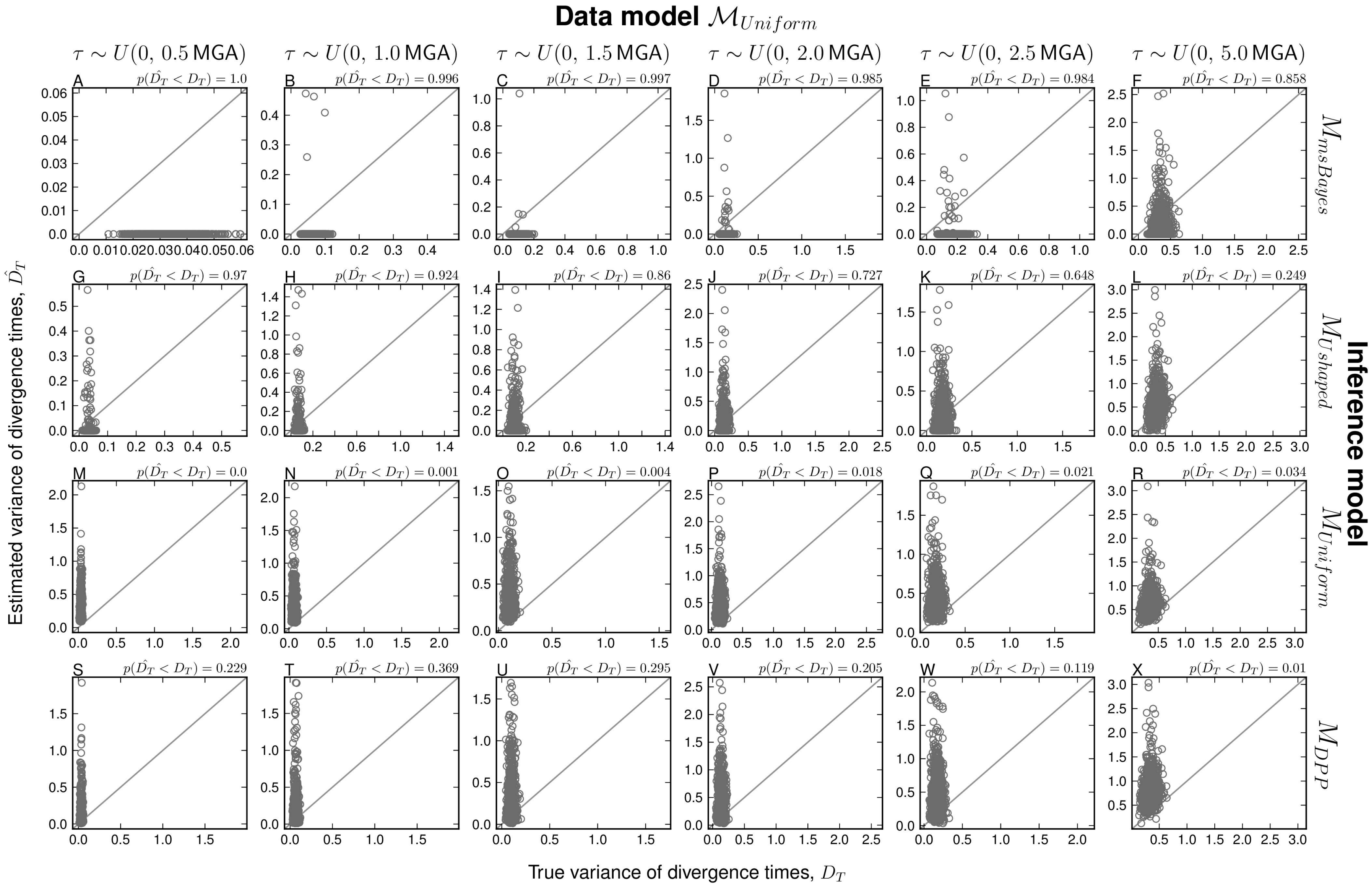

Figure S12: Accuracy of D_T estimates for models (A–F) $M_{msBayes}$, (G–L) $M_{Ushaped}$, (M–R) $M_{Uniform}$, and (S–X) M_{DPP} when analyzing data generated under the $\mathcal{M}_{Uniform}$ series of models. The true versus estimated value of the dispersion index of divergence times (D_T, in $4N_C$ generations) is plotted for 1000 datasets simulated under each of the $\mathcal{M}_{Uniform}$ models, and the proportion of estimates less than the truth, $p(\hat{D}_T < D_T)$, is shown for each data model. The 22 divergence times were randomly drawn as indicated above each column of plots, where time is respresented as millions of generations ago (MGA) according to a per-site rate of $1{\times}10^{-8}$ mutations per generation.

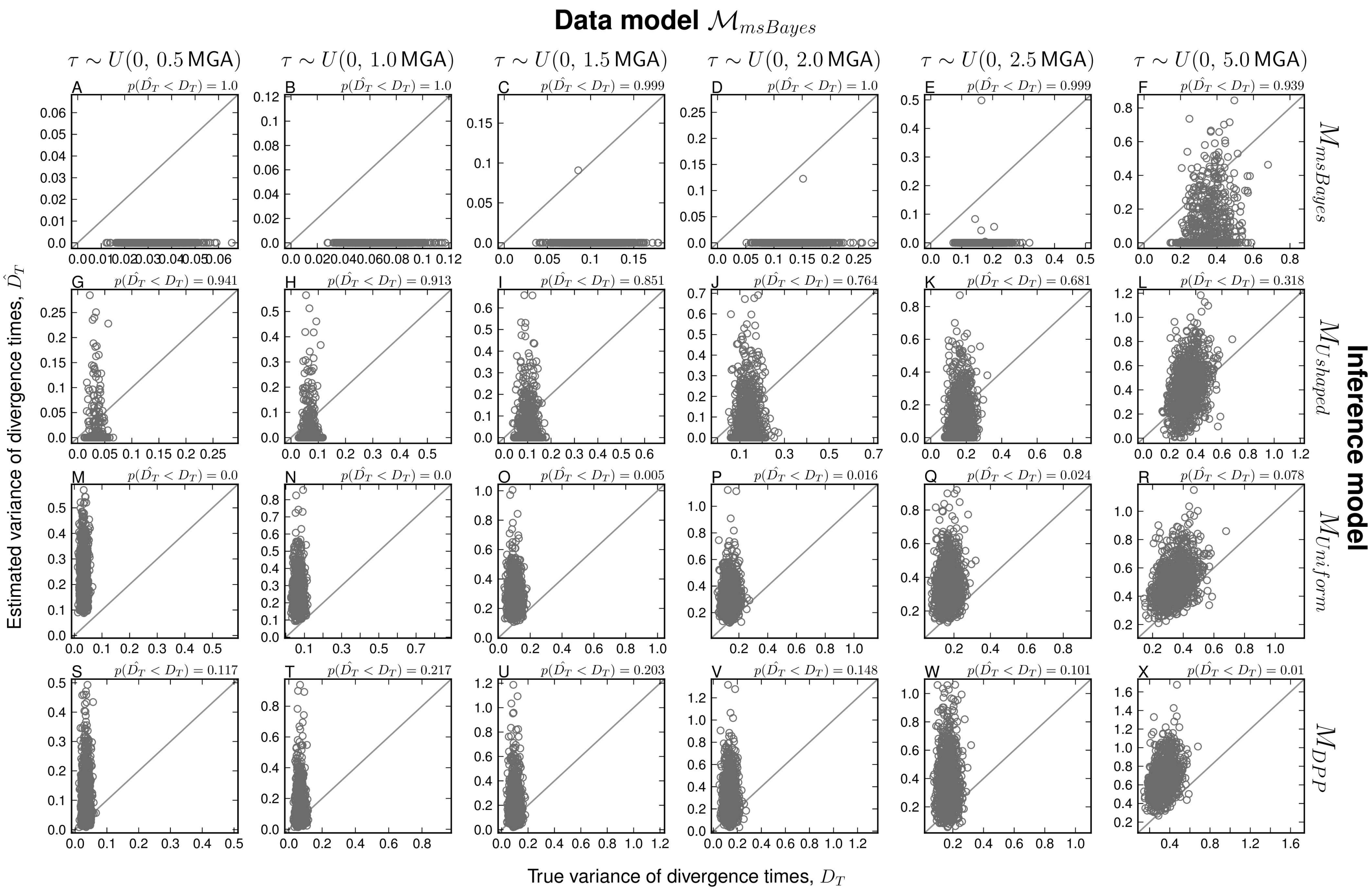

Figure S13: Accuracy of D_T estimates for models (A–F) $M_{msBayes}$, (G–L) $M_{Ushaped}$, (M–R) $M_{Uniform}$, and (S–X) M_{DPP} when analyzing data generated under the $\mathcal{M}_{msBayes}$ series of models. The true versus estimated value of the dispersion index of divergence times (D_T, in $4N_C$ generations) is plotted for 1000 datasets simulated under each of the $\mathcal{M}_{msBayes}$ models, and the proportion of estimates less than the truth, $p(\hat{D}_T < D_T)$, is shown for each data model. The 22 divergence times were randomly drawn as indicated above each column of plots, where time is respresented as millions of generations ago (MGA) according to a per-site rate of 1×10^{-8} mutations per generation.

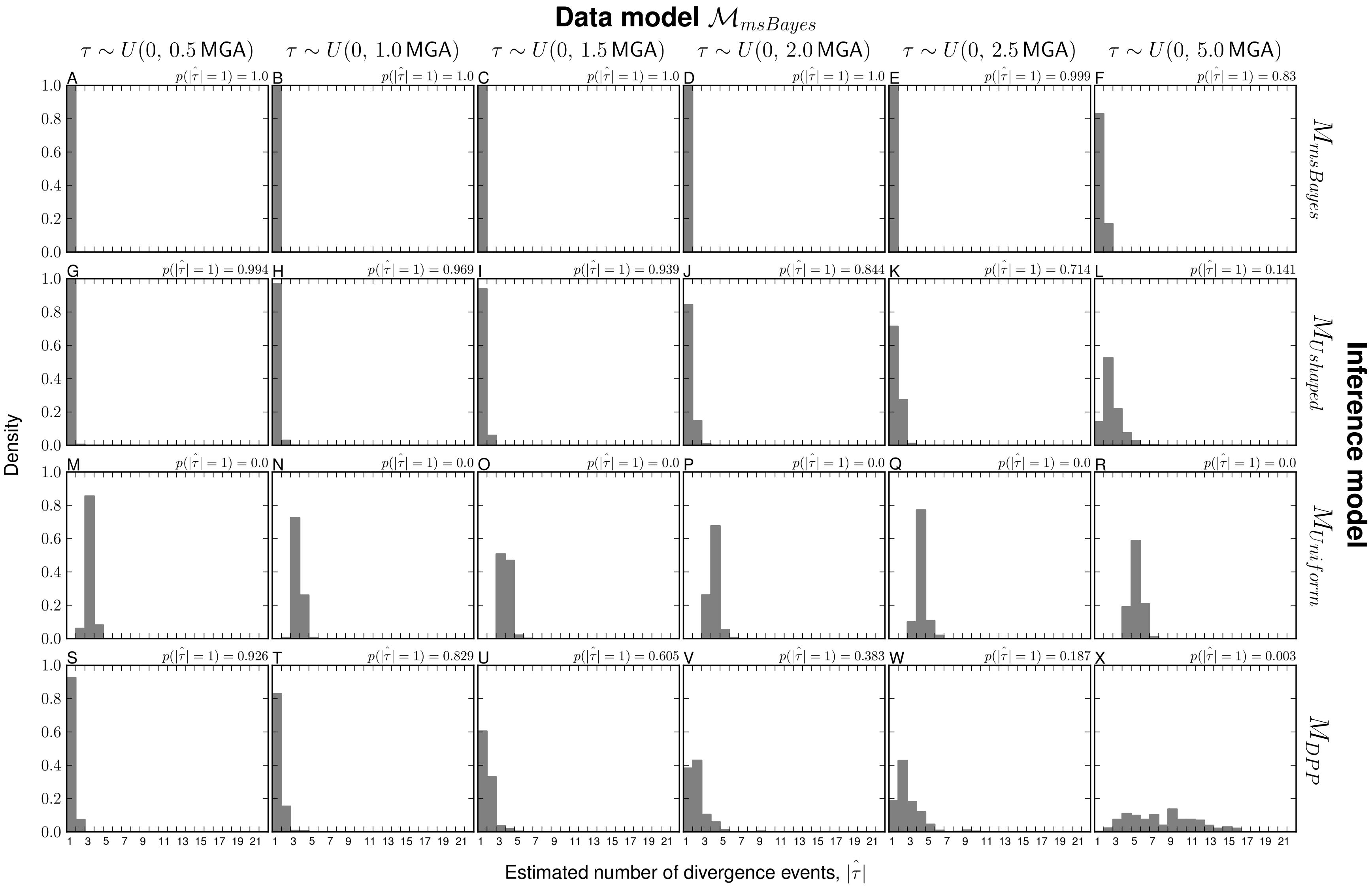

Figure S14: The power of models (A–F) $M_{msBayes}$, (G–L) $M_{Ushaped}$, (M–R) $M_{Uniform}$, and (S–X) M_{DPP} to detect random variation in divergence times as simulated under the $\mathcal{M}_{msBayes}$ series of models. The plots illustrate the estimated number of divergence events ($|\hat{\boldsymbol{\tau}}|$) from analyses of 1000 datasets simulated under each of the $\mathcal{M}_{msBayes}$ models, with the the estimated probability of the model inferring one divergence event, $p(|\hat{\boldsymbol{\tau}}| = 1)$, given for each combination. The 22 divergence times were randomly drawn as indicated above each column of plots, where time is respresented as millions of generations ago (MGA) according to a per-site rate of 1×10^{-8} mutations per generation.

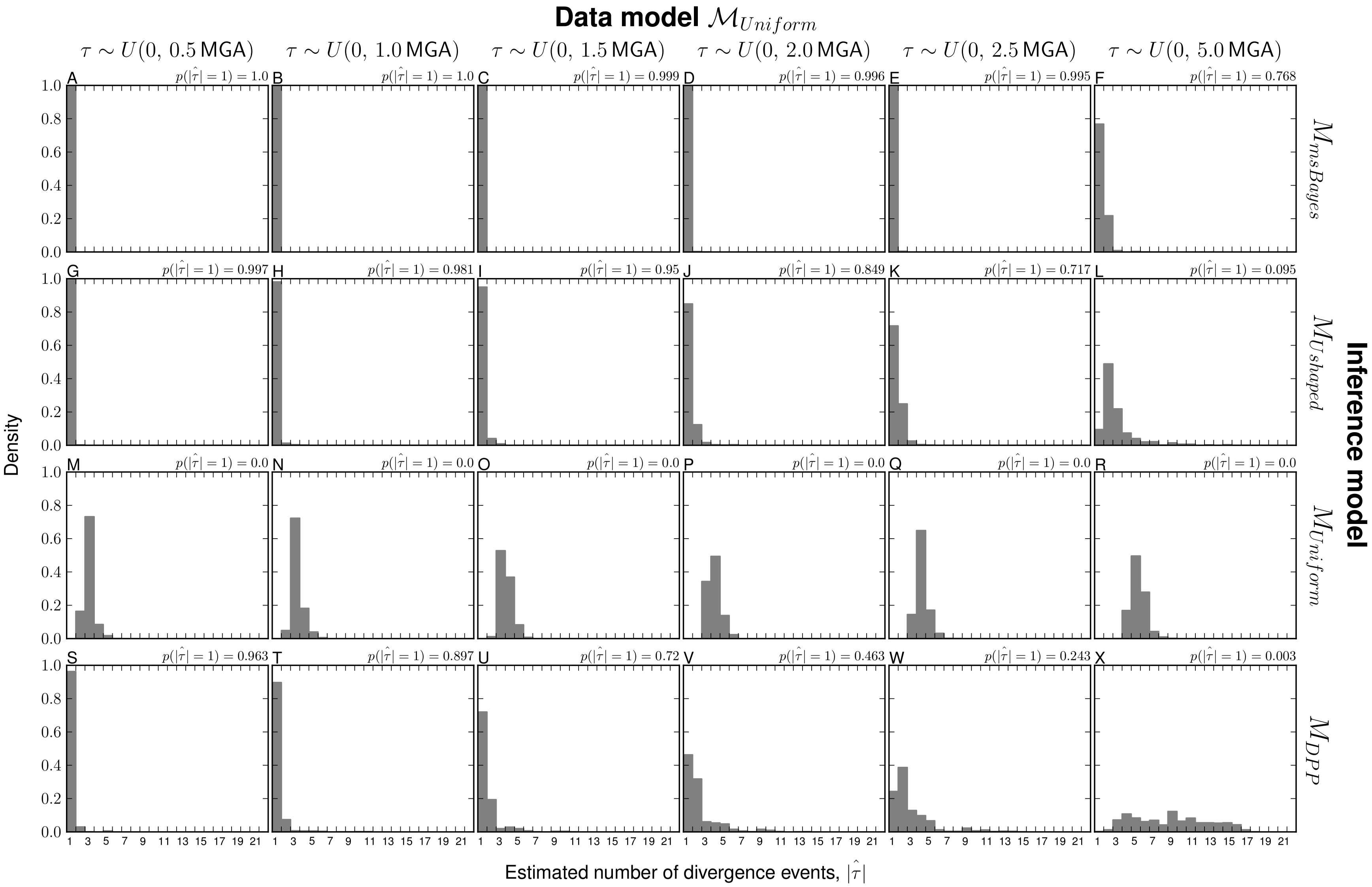

Figure S15: The power of models (A–F) $M_{msBayes}$, (G–L) $M_{Ushaped}$, (M–R) $M_{Uniform}$, and (S–X) M_{DPP} to detect random variation in divergence times as simulated under the $\mathcal{M}_{Uniform}$ series of models. The plots illustrate the estimated number of divergence events ($|\hat{\boldsymbol{\tau}}|$) from analyses of 1000 datasets simulated under each of the $\mathcal{M}_{Uniform}$ models, with the the estimated probability of the model inferring one divergence event, $p(|\hat{\boldsymbol{\tau}}| = 1)$, given for each combination. The 22 divergence times were randomly drawn as indicated above each column of plots, where time is respresented as millions of generations ago (MGA) according to a per-site rate of $1{\times}10^{-8}$ mutations per generation.

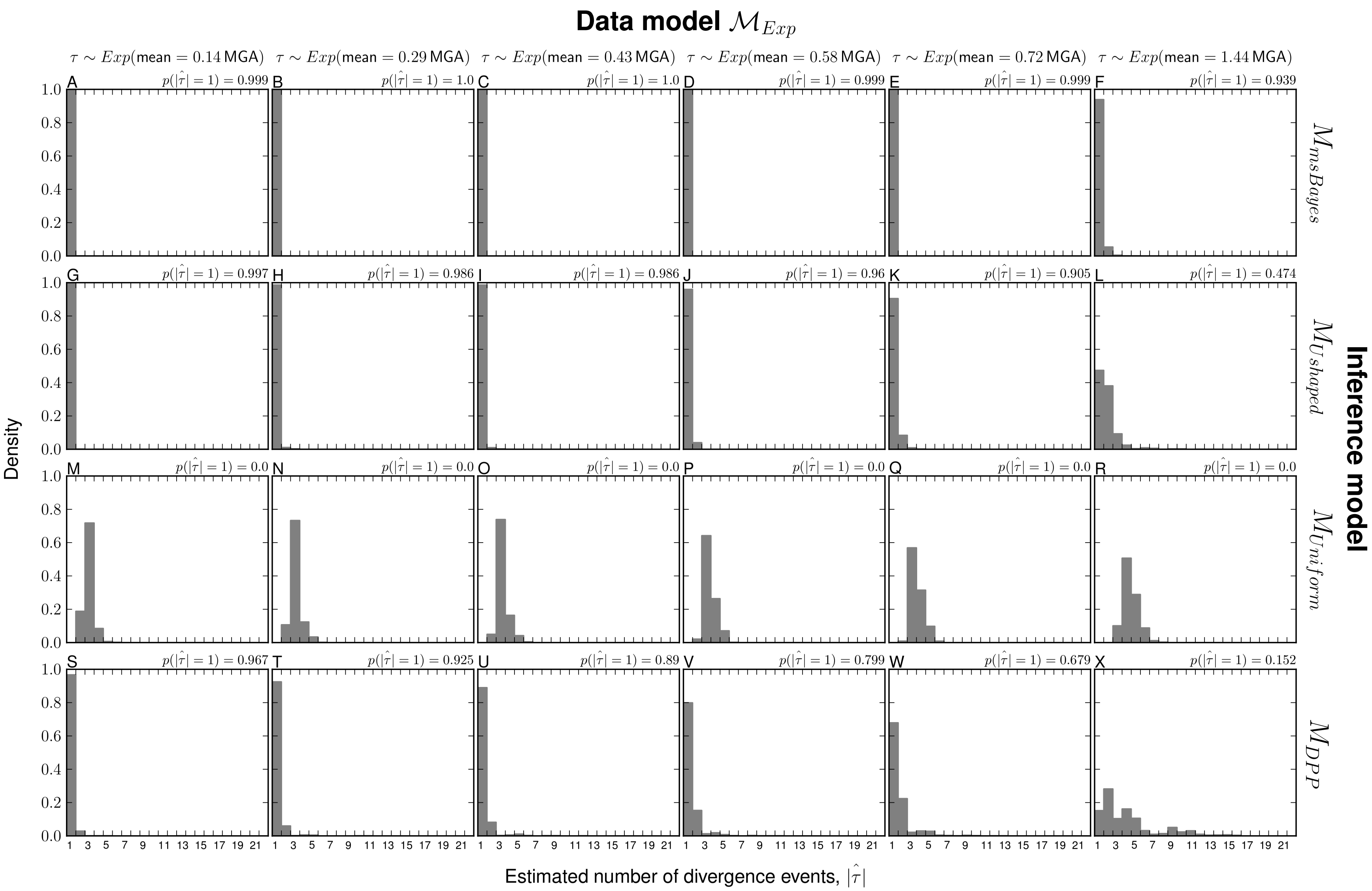

Figure S16: The power of models (A–F) $M_{msBayes}$, (G–L) $M_{Ushaped}$, (M–R) $M_{Uniform}$, and (S–X) M_{DPP} to detect random variation in divergence times as simulated under the $\mathcal{M}_{Exp}$ series of models. The plots illustrate the estimated number of divergence events ($|\hat{\boldsymbol{\tau}}|$) from analyses of 1000 datasets simulated under each of the $\mathcal{M}_{Exp}$ models, with the the estimated probability of the model inferring one divergence event, $p(|\hat{\boldsymbol{\tau}}| = 1)$, given for each combination. The 22 divergence times were randomly drawn as indicated above each column of plots, where time is respresented as millions of generations ago (MGA) according to a per-site rate of 1×10^{-8} mutations per generation.

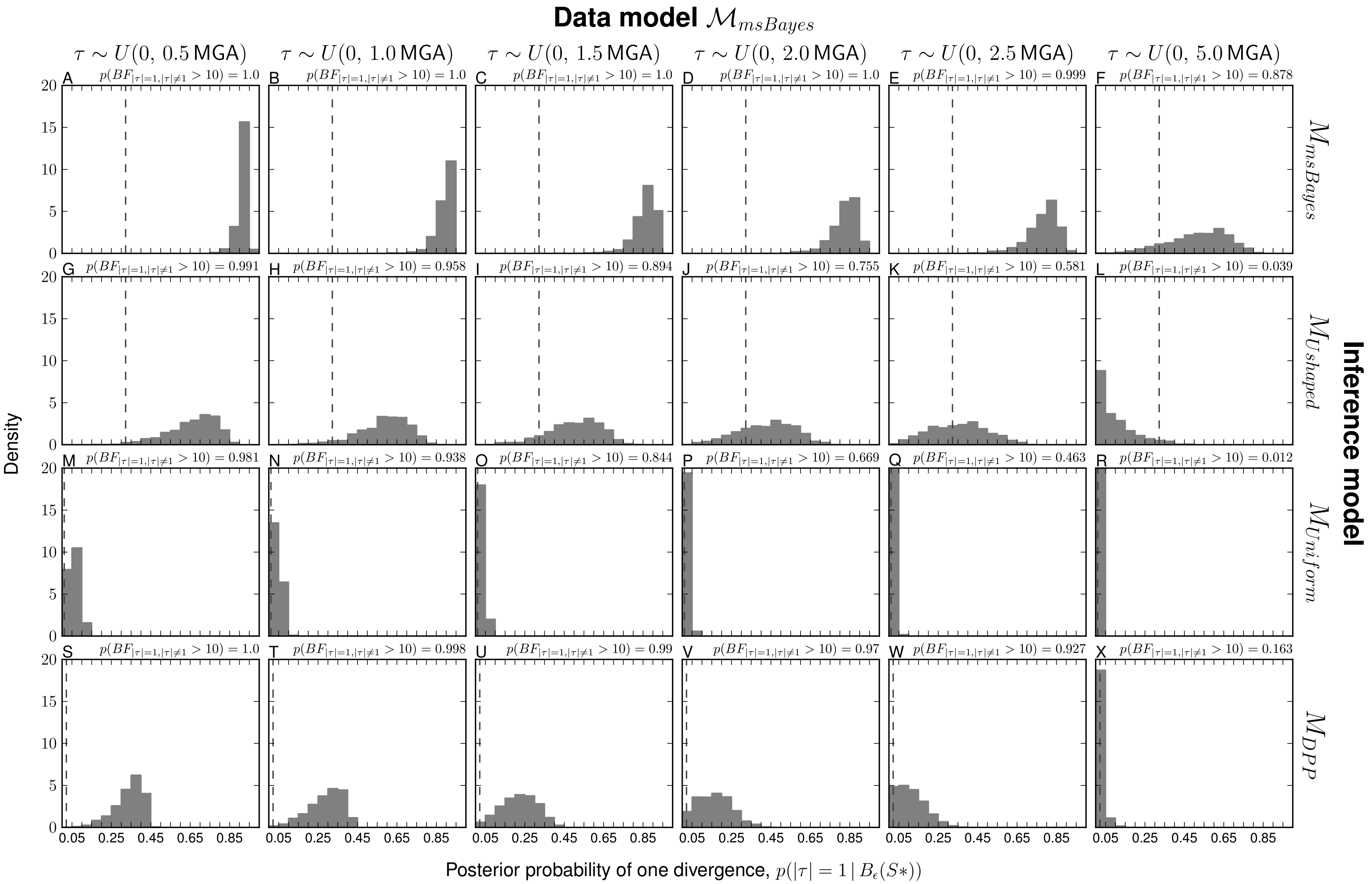

Figure S17: The tendency of models (A–F) $M_{msBayes}$, (G–L) $M_{Ushaped}$, (M–R) $M_{Uniform}$, and (S–X) M_{DPP} to support one divergence event when there is random variation in divergence times as simulated under the $\mathcal{M}_{msBayes}$ series of models. The plots illustrate histograms of the estimated posterior probability of the one divergence model, $p(|\boldsymbol{\tau}| = 1|B_\epsilon(\mathbf{S}^*))$, from analyses of 1000 datasets simulated under each of the $\mathcal{M}_{msBayes}$ models. The 22 divergence times were randomly drawn as indicated above each column of plots, where time is respresented as millions of generations ago (MGA) according to a per-site rate of $1{\times}10^{-8}$ mutations per generation.

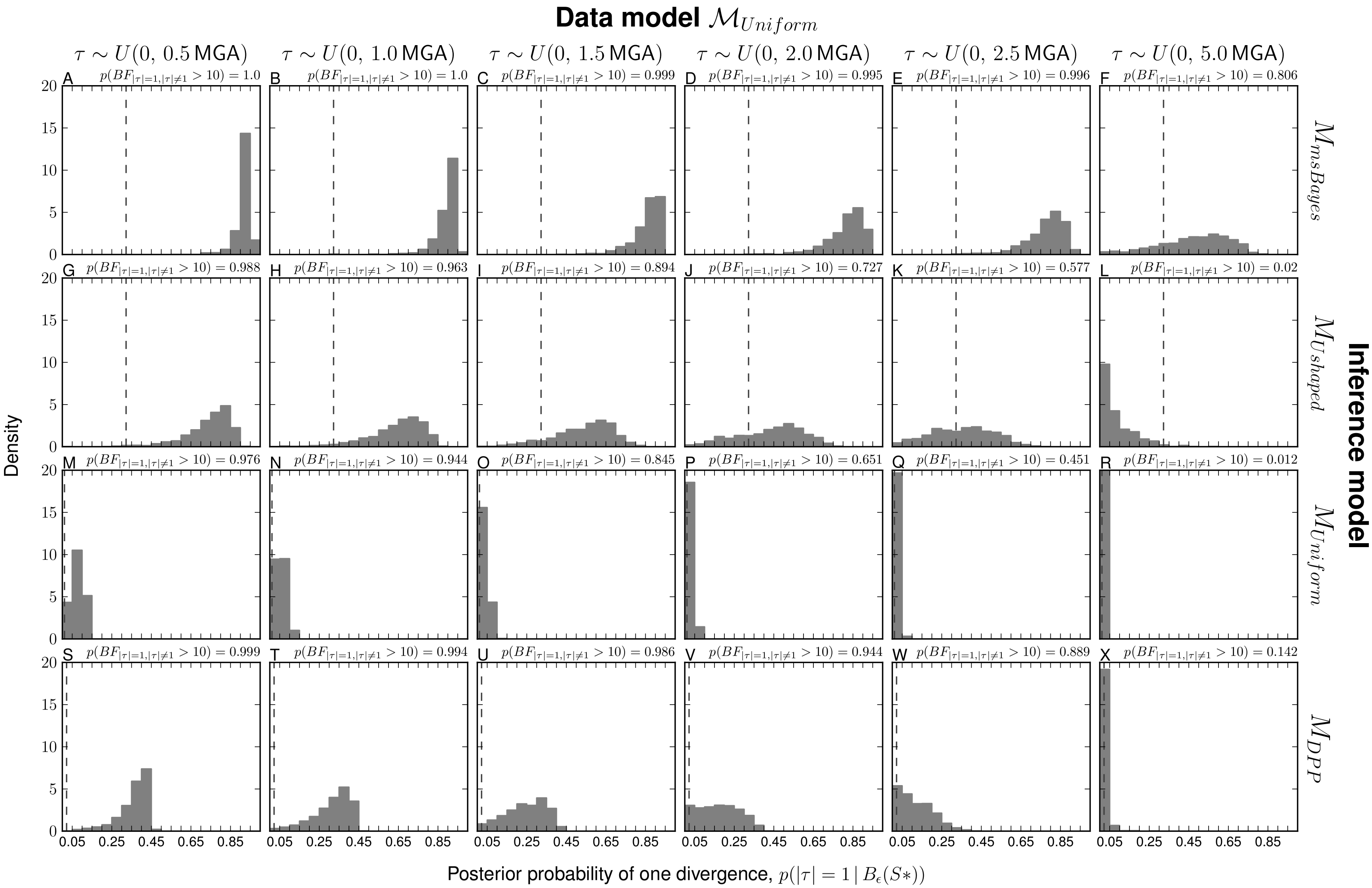

Figure S18: The tendency of models (A–F) $M_{msBayes}$, (G–L) $M_{Ushaped}$, (M–R) $M_{Uniform}$, and (S–X) M_{DPP} to support one divergence event when there is random variation in divergence times as simulated under the $\mathcal{M}_{Uniform}$ series of models. The plots illustrate histograms of the estimated posterior probability of the one divergence model, $p(|\boldsymbol{\tau}| = 1|B_\epsilon(\mathbf{S}^*))$, from analyses of 1000 datasets simulated under each of the $\mathcal{M}_{Uniform}$ models. The 22 divergence times were randomly drawn as indicated above each column of plots, where time is respresented as millions of generations ago (MGA) according to a per-site rate of 1×10^{-8} mutations per generation.

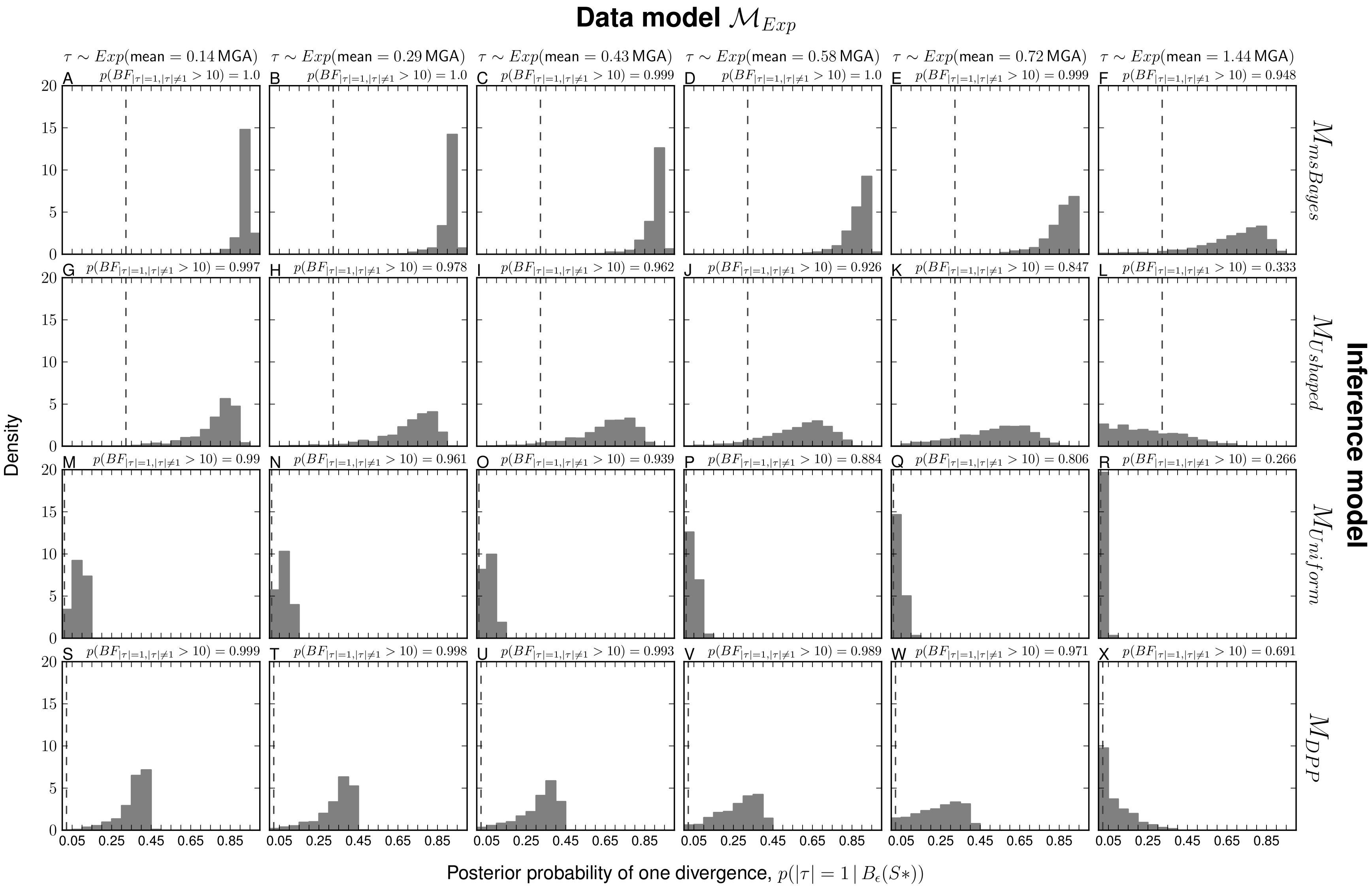

Figure S19: The tendency of models (A–F) $M_{msBayes}$, (G–L) $M_{Ushaped}$, (M–R) $M_{Uniform}$, and (S–X) M_{DPP} to support one divergence event when there is random variation in divergence times as simulated under the $\mathcal{M}_{Exp}$ series of models. The plots illustrate histograms of the estimated posterior probability of the one divergence model, $p(|\boldsymbol{\tau}| = 1|B_\epsilon(\mathbf{S}^*))$, from analyses of 1000 datasets simulated under each of the $\mathcal{M}_{Exp}$ models. The 22 divergence times were randomly drawn as indicated above each column of plots, where time is respresented as millions of generations ago (MGA) according to a per-site rate of 1×10^{-8} mutations per generation.

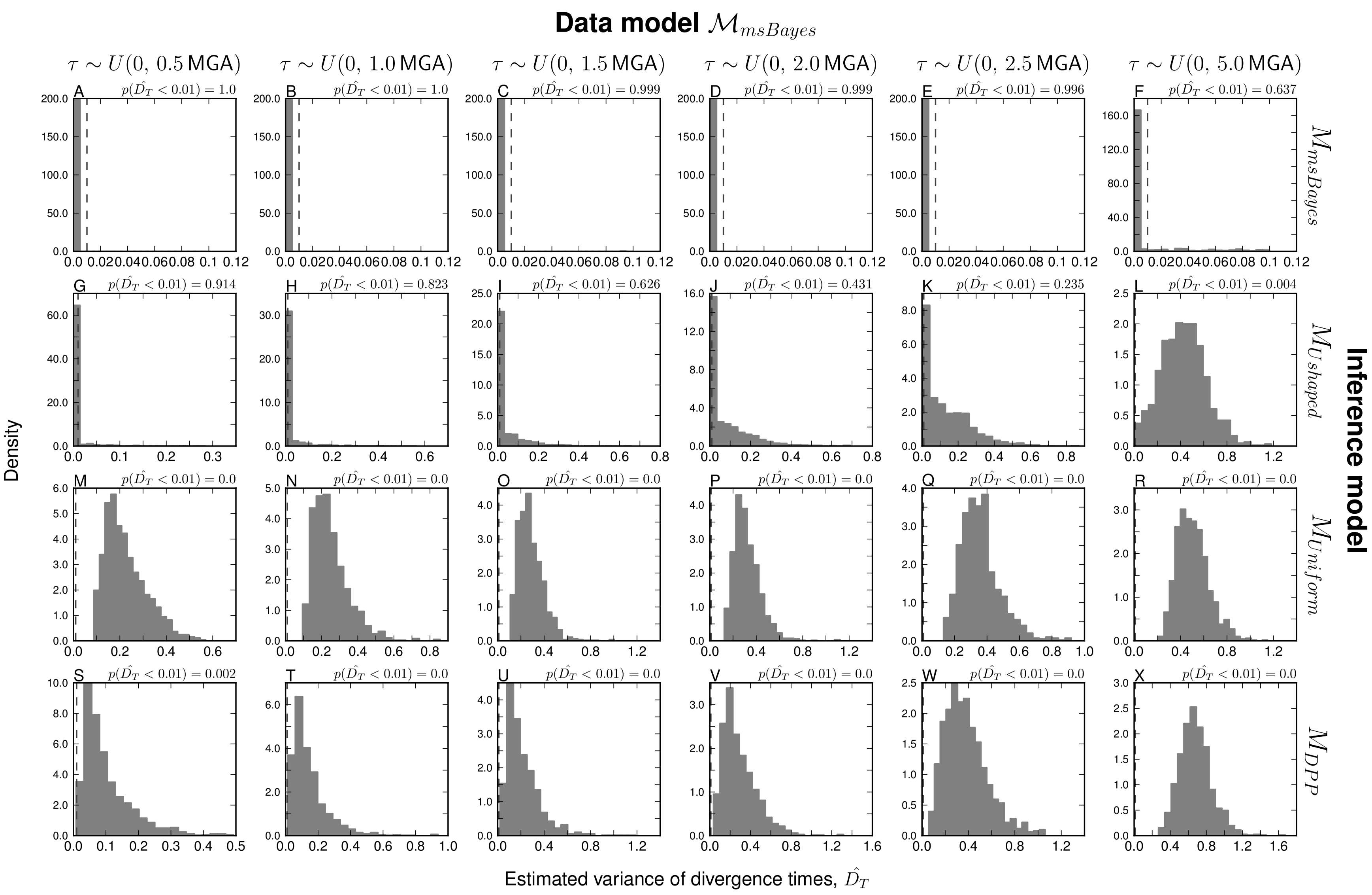

Figure S20: The power of models (A–F) $M_{msBayes}$, (G–L) $M_{Ushaped}$, (M–R) $M_{Uniform}$, and (S–X) M_{DPP} to detect random variation in divergence times as simulated under the $\mathcal{M}_{msBayes}$ series of models. The plots illustrate the estimated dispersion index of divergence times ($\hat{D}_T$) from analyses of 1000 datasets simulated under each of the $\mathcal{M}_{msBayes}$ models, with the the estimated probability of the model inferring one divergence event, $p(\hat{D}_T < 0.01)$, given for each combination. The 22 divergence times were randomly drawn as indicated above each column of plots, where time is respresented as millions of generations ago (MGA) according to a per-site rate of $1{\times}10^{-8}$ mutations per generation.

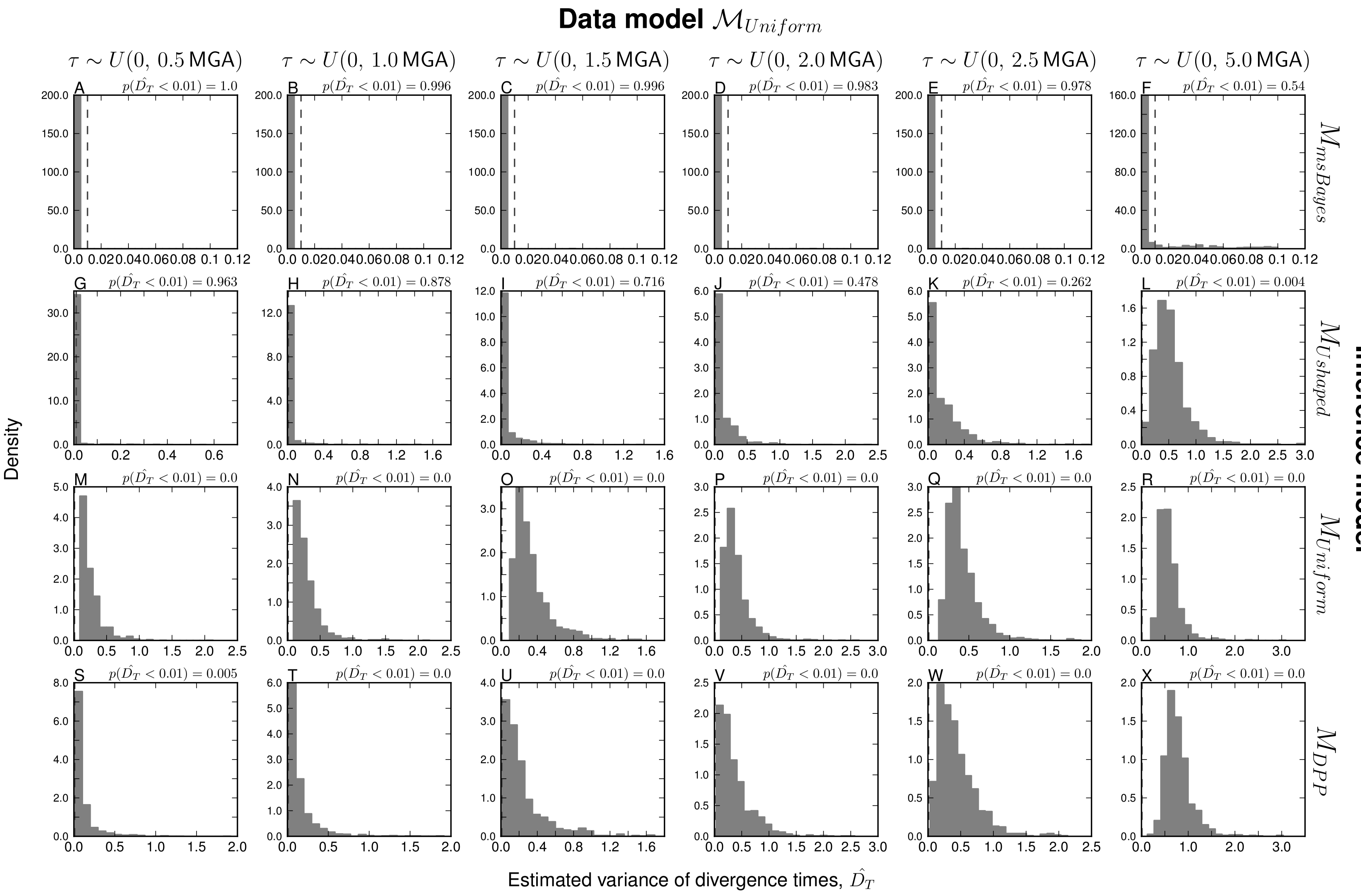

Figure S21: The power of models (A–F) $M_{msBayes}$, (G–L) $M_{Ushaped}$, (M–R) $M_{Uniform}$, and (S–X) M_{DPP} to detect random variation in divergence times as simulated under the $\mathcal{M}_{Uniform}$ series of models. The plots illustrate the estimated dispersion index of divergence times ($\hat{D}_T$) from analyses of 1000 datasets simulated under each of the $\mathcal{M}_{Uniform}$ models, with the the estimated probability of the model inferring one divergence event, $p(\hat{D}_T < 0.01)$, given for each combination. The 22 divergence times were randomly drawn as indicated above each column of plots, where time is respresented as millions of generations ago (MGA) according to a per-site rate of $1{\times}10^{-8}$ mutations per generation.

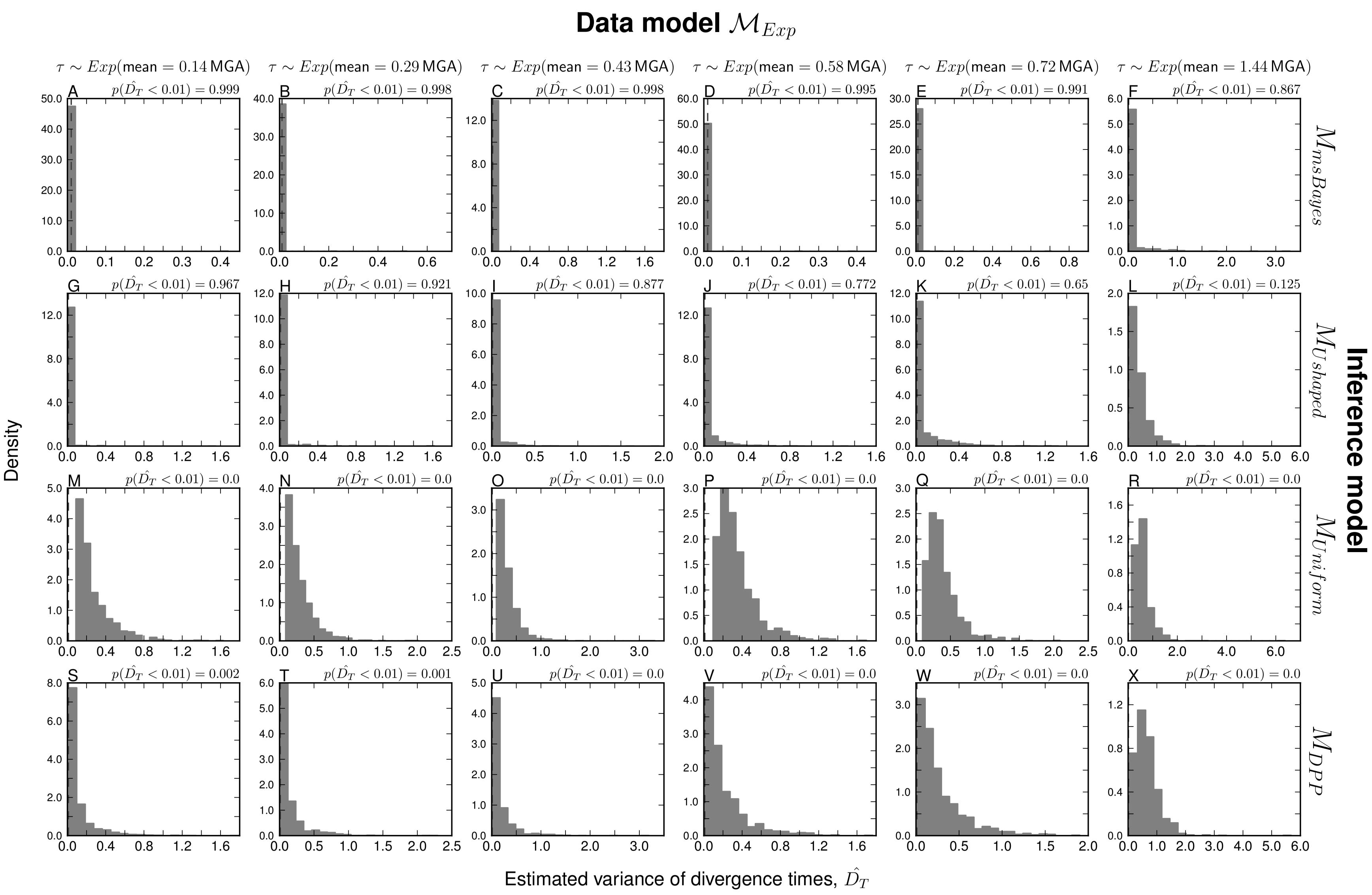

Figure S22: The power of models (A–F) $M_{msBayes}$, (G–L) $M_{Ushaped}$, (M–R) $M_{Uniform}$, and (S–X) M_{DPP} to detect random variation in divergence times as simulated under the $\mathcal{M}_{Exp}$ series of models. The plots illustrate the estimated dispersion index of divergence times ($\hat{D}_T$) from analyses of 1000 datasets simulated under each of the $\mathcal{M}_{Exp}$ models, with the the estimated probability of the model inferring one divergence event, $p(\hat{D}_T < 0.01)$, given for each combination. The 22 divergence times were randomly drawn as indicated above each column of plots, where time is respresented as millions of generations ago (MGA) according to a per-site rate of 1×10^{-8} mutations per generation.

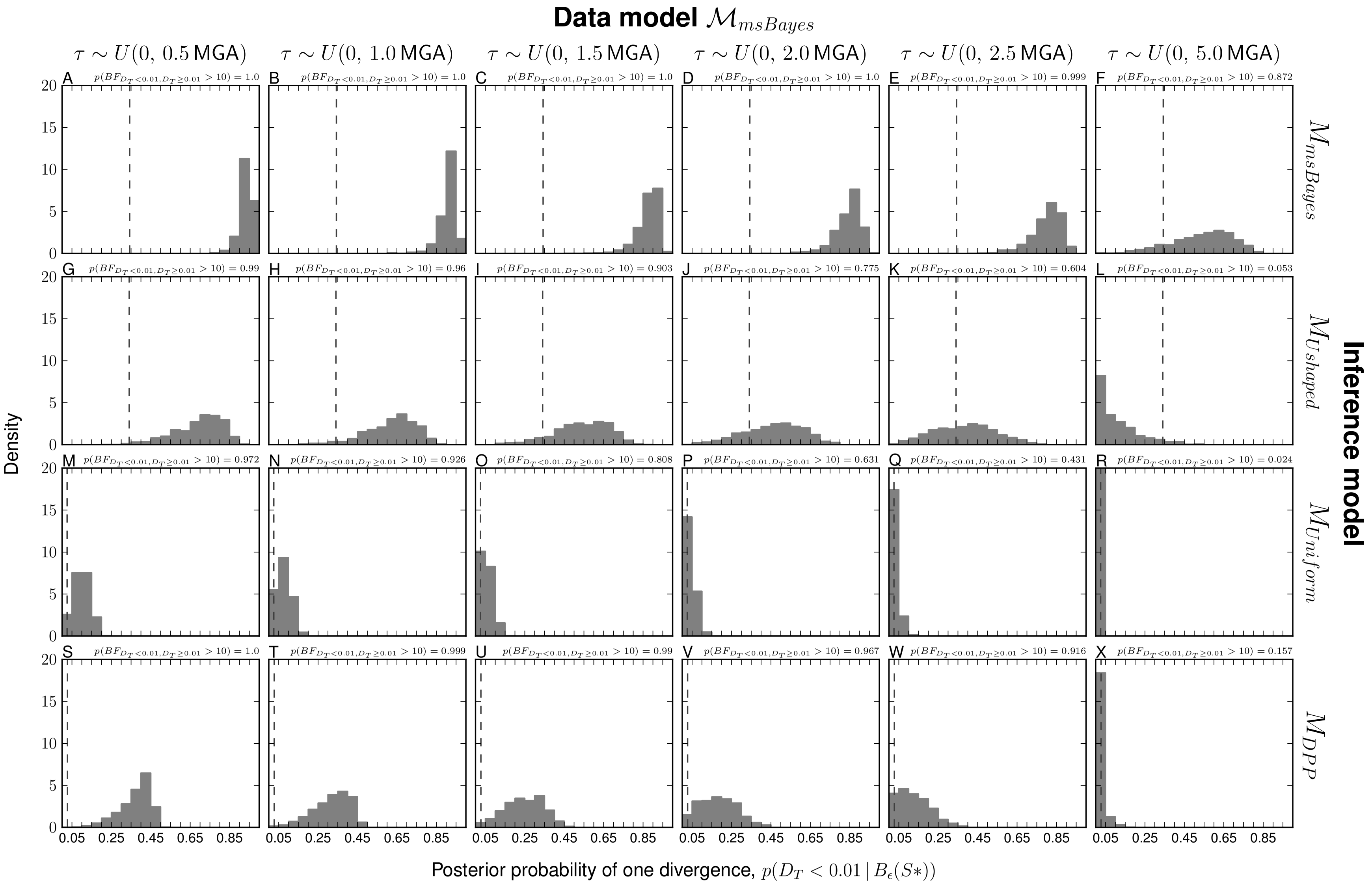

Figure S23: The tendency of models (A–F) $M_{msBayes}$, (G–L) $M_{Ushaped}$, (M–R) $M_{Uniform}$, and (S–X) M_{DPP} to support one divergence event when there is random variation in divergence times as simulated under the $\mathcal{M}_{msBayes}$ series of models. The plots illustrate histograms of the estimated posterior probability of the one divergence model, $p(D_T < 0.01|B_\epsilon(\mathbf{S}^*))$, from analyses of 1000 datasets simulated under each of the $\mathcal{M}_{msBayes}$ models. The 22 divergence times were randomly drawn as indicated above each column of plots, where time is respresented as millions of generations ago (MGA) according to a per-site rate of $1{\times}10^{-8}$ mutations per generation.

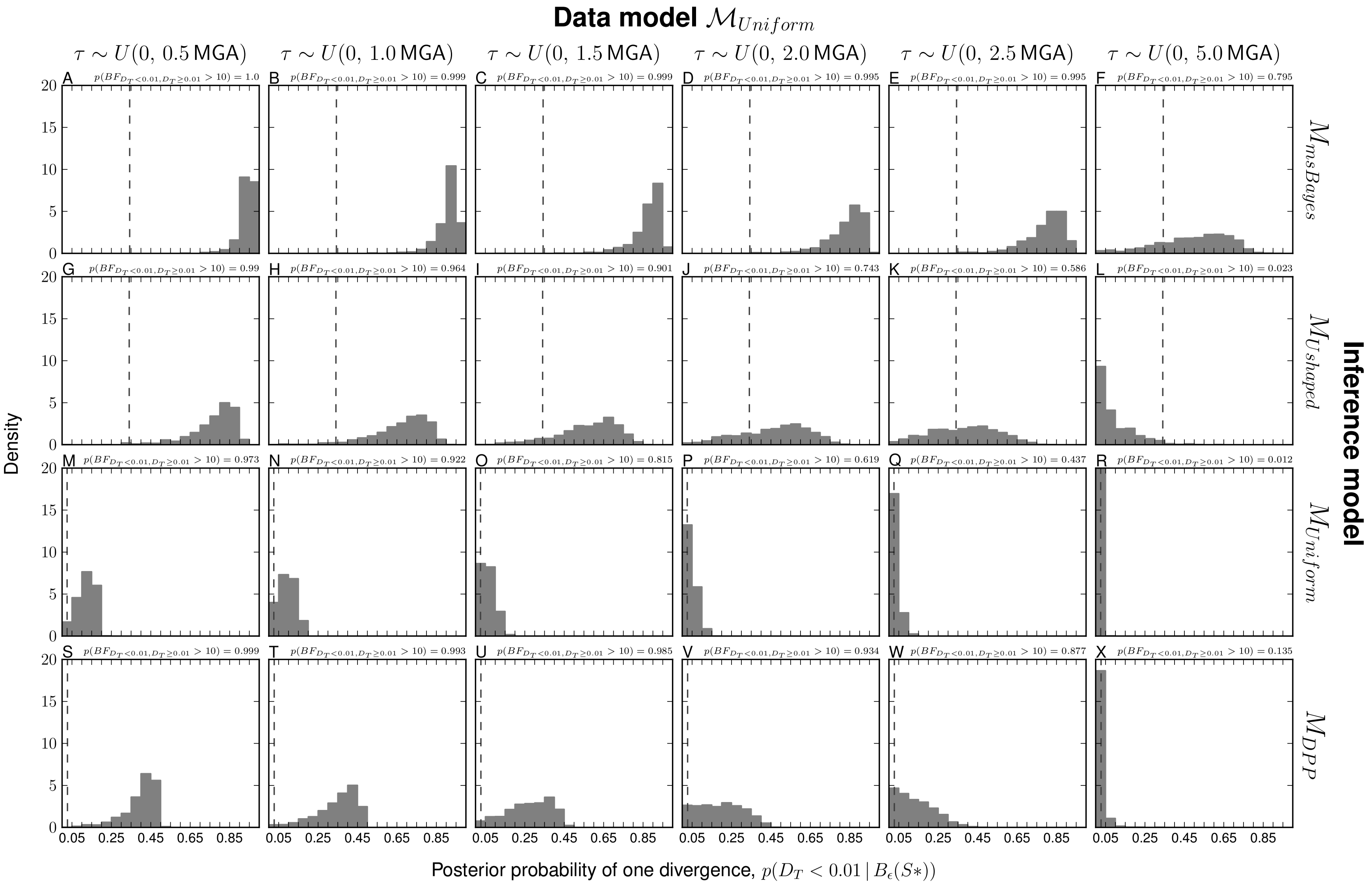

Figure S24: The tendency of models (A–F) $M_{msBayes}$, (G–L) $M_{Ushaped}$, (M–R) $M_{Uniform}$, and (S–X) M_{DPP} to support one divergence event when there is random variation in divergence times as simulated under the $\mathcal{M}_{Uniform}$ series of models. The plots illustrate histograms of the estimated posterior probability of the one divergence model, $p(D_T < 0.01|B_\epsilon(\mathbf{S}^*))$, from analyses of 1000 datasets simulated under each of the $\mathcal{M}_{Uniform}$ models. The 22 divergence times were randomly drawn as indicated above each column of plots, where time is respresented as millions of generations ago (MGA) according to a per-site rate of 1×10^{-8} mutations per generation.

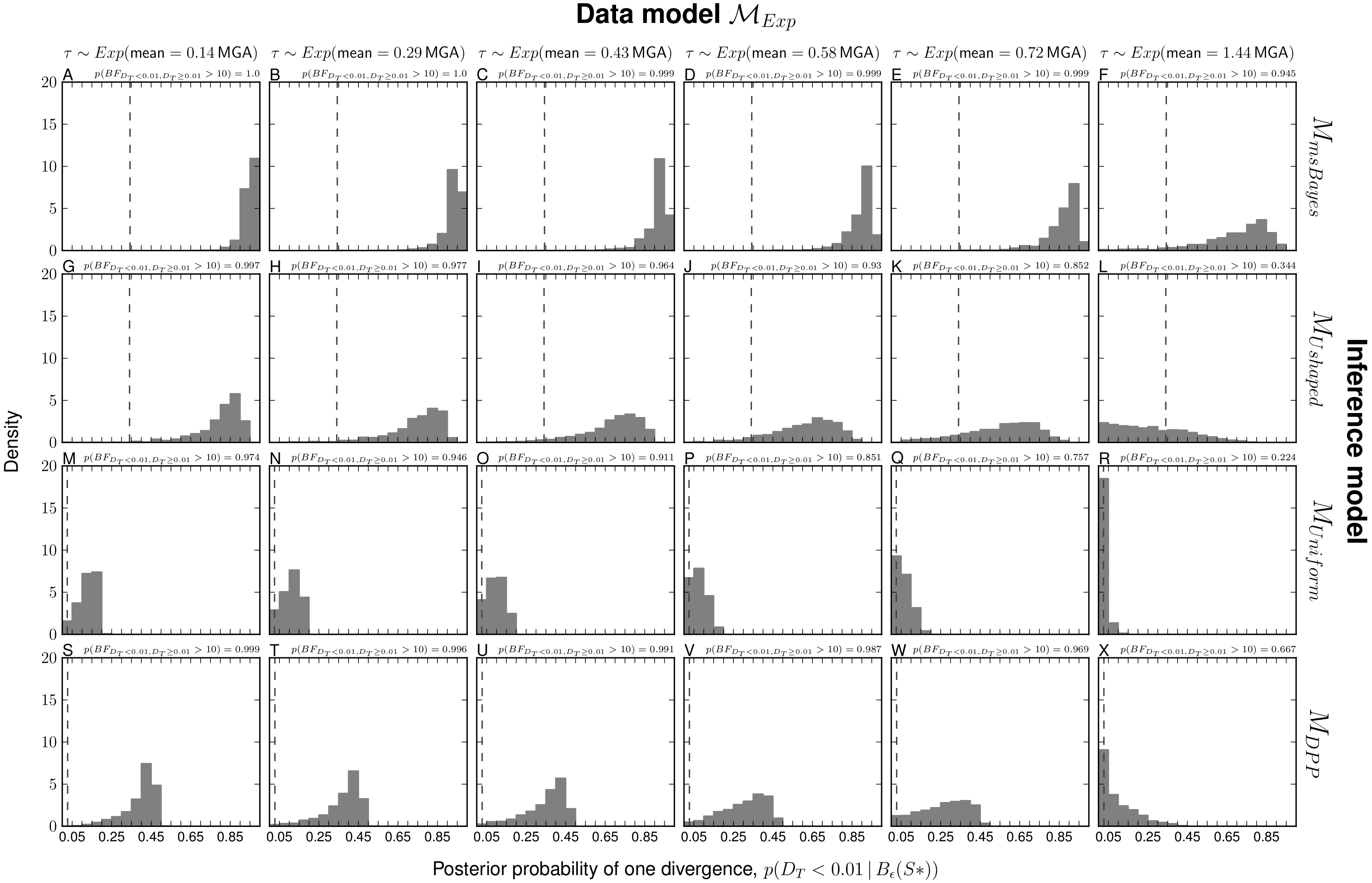

Figure S25: The tendency of models (A–F) $M_{msBayes}$, (G–L) $M_{Ushaped}$, (M–R) $M_{Uniform}$, and (S–X) M_{DPP} to support one divergence event when there is random variation in divergence times as simulated under the $\mathcal{M}_{Exp}$ series of models. The plots illustrate histograms of the estimated posterior probability of the one divergence model, $p(D_T < 0.01|B_\epsilon(\mathbf{S}^*))$, from analyses of 1000 datasets simulated under each of the $\mathcal{M}_{Exp}$ models. The 22 divergence times were randomly drawn as indicated above each column of plots, where time is respresented as millions of generations ago (MGA) according to a per-site rate of 1×10^{-8} mutations per generation.

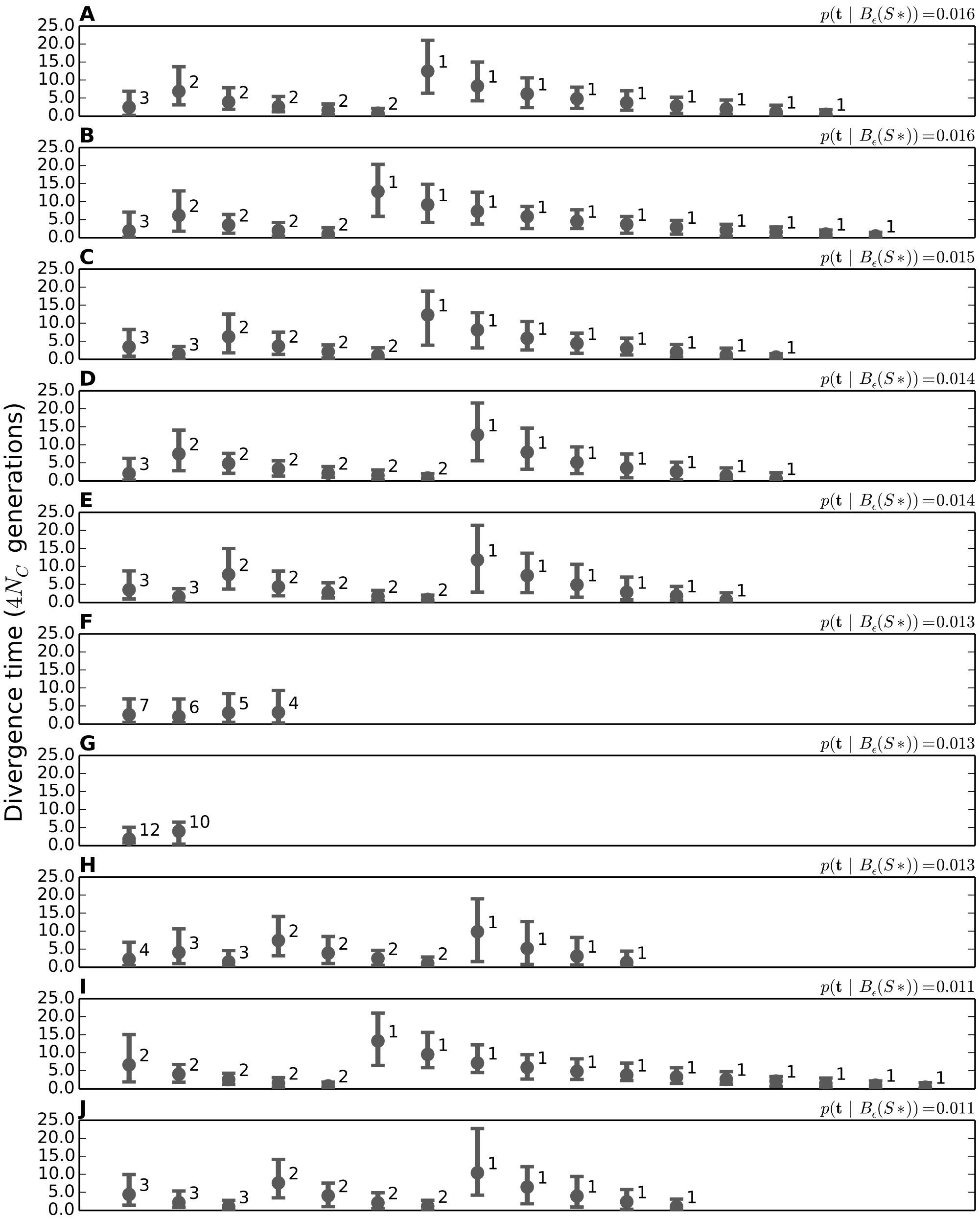

Figure S26: The divergence-model results when the 22 pairs of taxa from the Philippines are analyzed under the $\mathbf{M}_{DPP}$ model (Table 4). The 10 unordered divergence models with highest posterior probability ($p(\mathbf{t} \mid B_\epsilon(\mathbf{S}^*))$) are shown, where the numbers indicate the inferred number of taxon pairs that diverged at each event. The times indicate the posterior median and 95% highest posterior density (HPD) interval conditional on each divergence model. For each model, times are summarized across posterior samples by the number of taxon pairs associated with each divergence. For models in which there are multiple divergence events with the same number of taxon pairs, the events are sorted by time to summarize the divergence times in a consistant way.

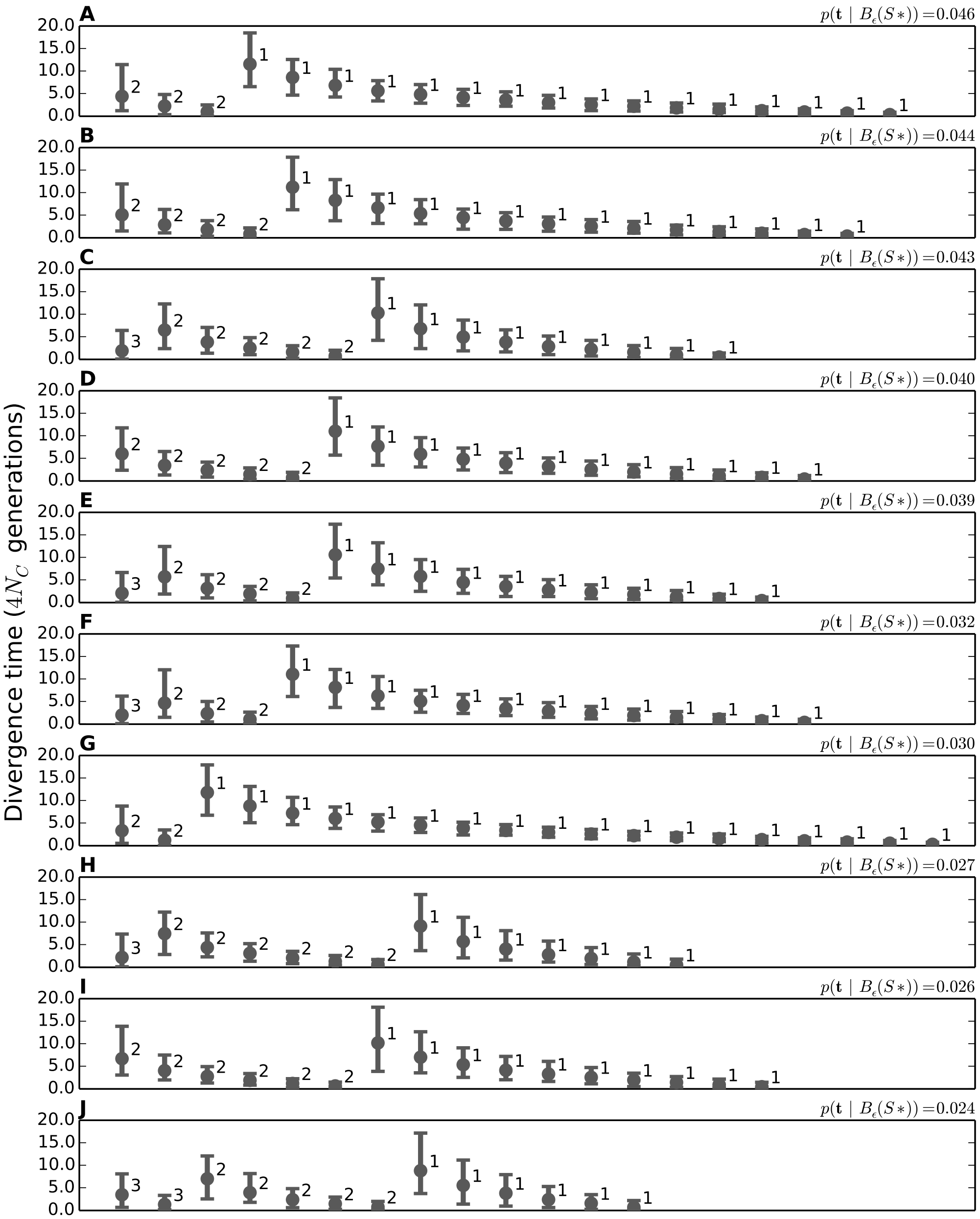

Figure S27: The divergence-model results when the 22 pairs of taxa from the Philippines are analyzed under the $\mathbf{M}_{DPP}^{inform}$ model (Table 4). The 10 unordered divergence models with highest posterior probability ($p(\mathbf{t} \mid B_{\epsilon}(\mathbf{S}^{*}))$) are shown, where the numbers indicate the inferred number of taxon pairs that diverged at each event. The times indicate the posterior median and 95% highest posterior density (HPD) interval conditional on each divergence model. For each model, times are summarized across posterior samples by the number of taxon pairs associated with each divergence. For models in which there are multiple divergence events with the same number of taxon pairs, the events are sorted by time to summarize the divergence times in a consistant way.

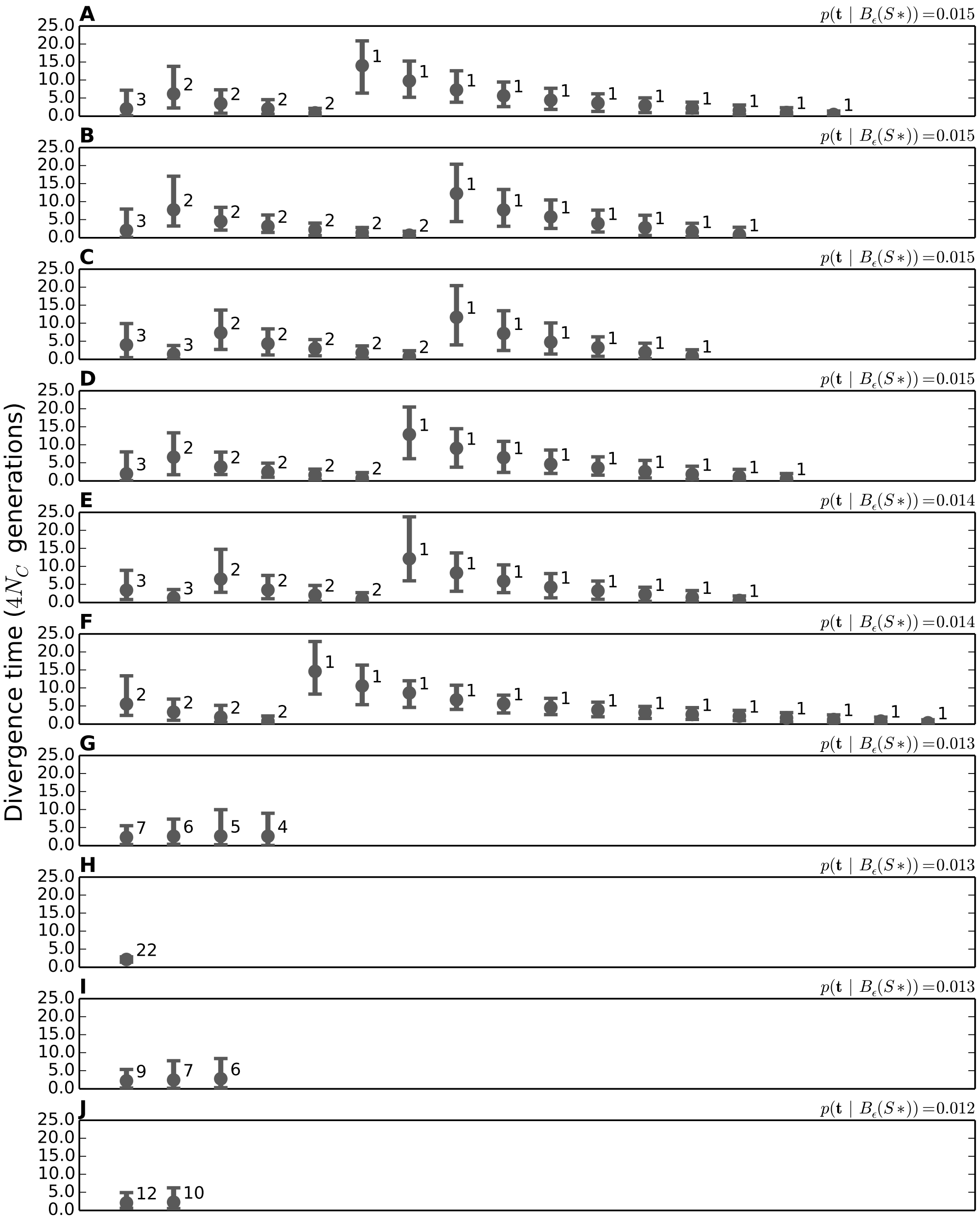

Figure S28: The divergence-model results when the 22 pairs of taxa from the Philippines are analyzed under the $\mathbf{M}_{DPP}^{simple}$ model (Table 4). The 10 unordered divergence models with highest posterior probability ($p(\mathbf{t} \mid B_\epsilon(\mathbf{S}^*))$) are shown, where the numbers indicate the inferred number of taxon pairs that diverged at each event. The times indicate the posterior median and 95% highest posterior density (HPD) interval conditional on each divergence model. For each model, times are summarized across posterior samples by the number of taxon pairs associated with each divergence. For models in which there are multiple divergence events with the same number of taxon pairs, the events are sorted by time to summarize the divergence times in a consistant way.

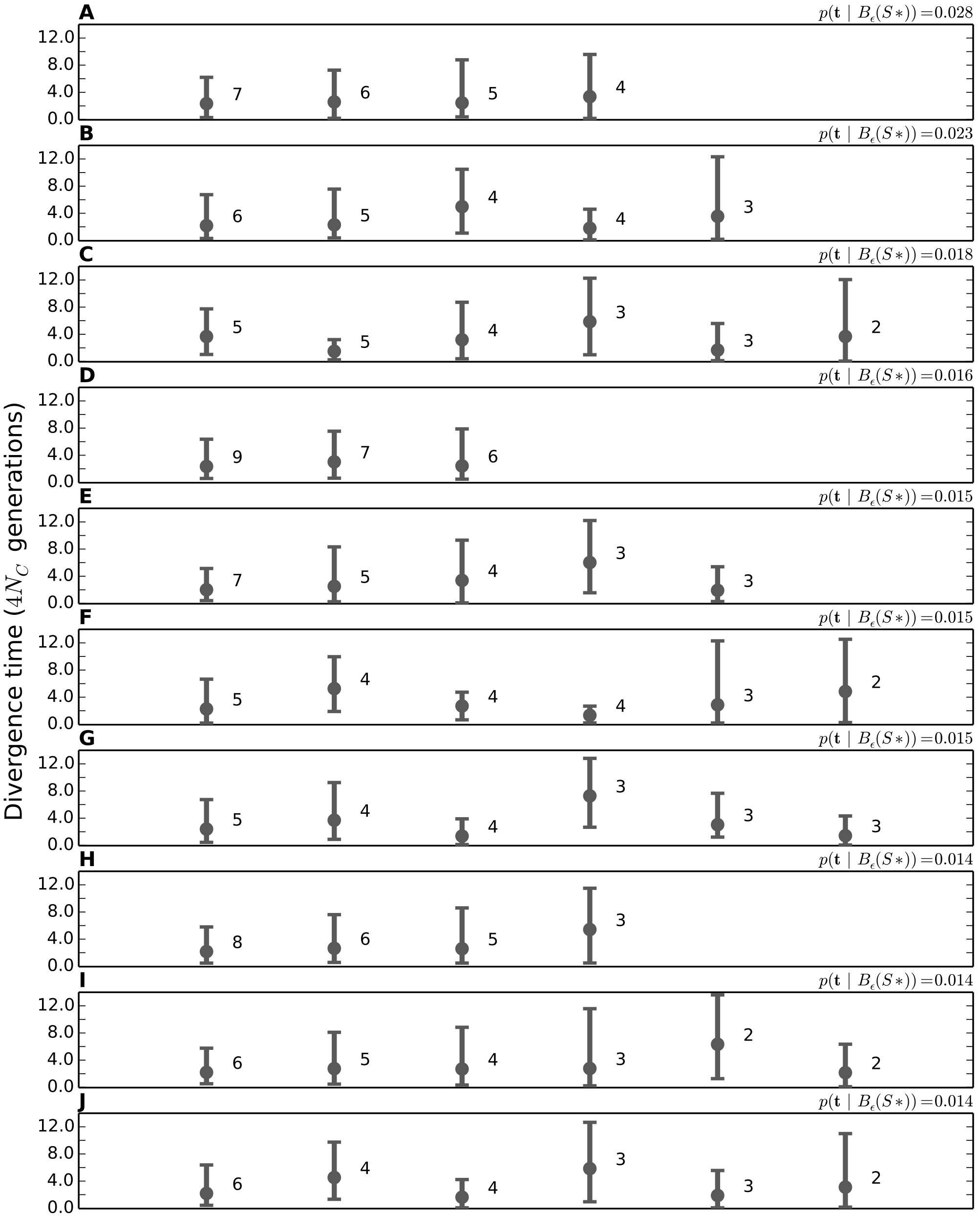

Figure S29: The divergence-model results when the 22 pairs of taxa from the Philippines are analyzed under the $\mathbf{M}_{Uniform}$ model (Table 4). The 10 unordered divergence models with highest posterior probability ($p(\mathbf{t} \mid B_\epsilon(\mathbf{S}^*))$) are shown, where the numbers indicate the inferred number of taxon pairs that diverged at each event. The times indicate the posterior median and 95% highest posterior density (HPD) interval conditional on each divergence model. For each model, times are summarized across posterior samples by the number of taxon pairs associated with each divergence. For models in which there are multiple divergence events with the same number of taxon pairs, the events are sorted by time to summarize the divergence times in a consistant way.

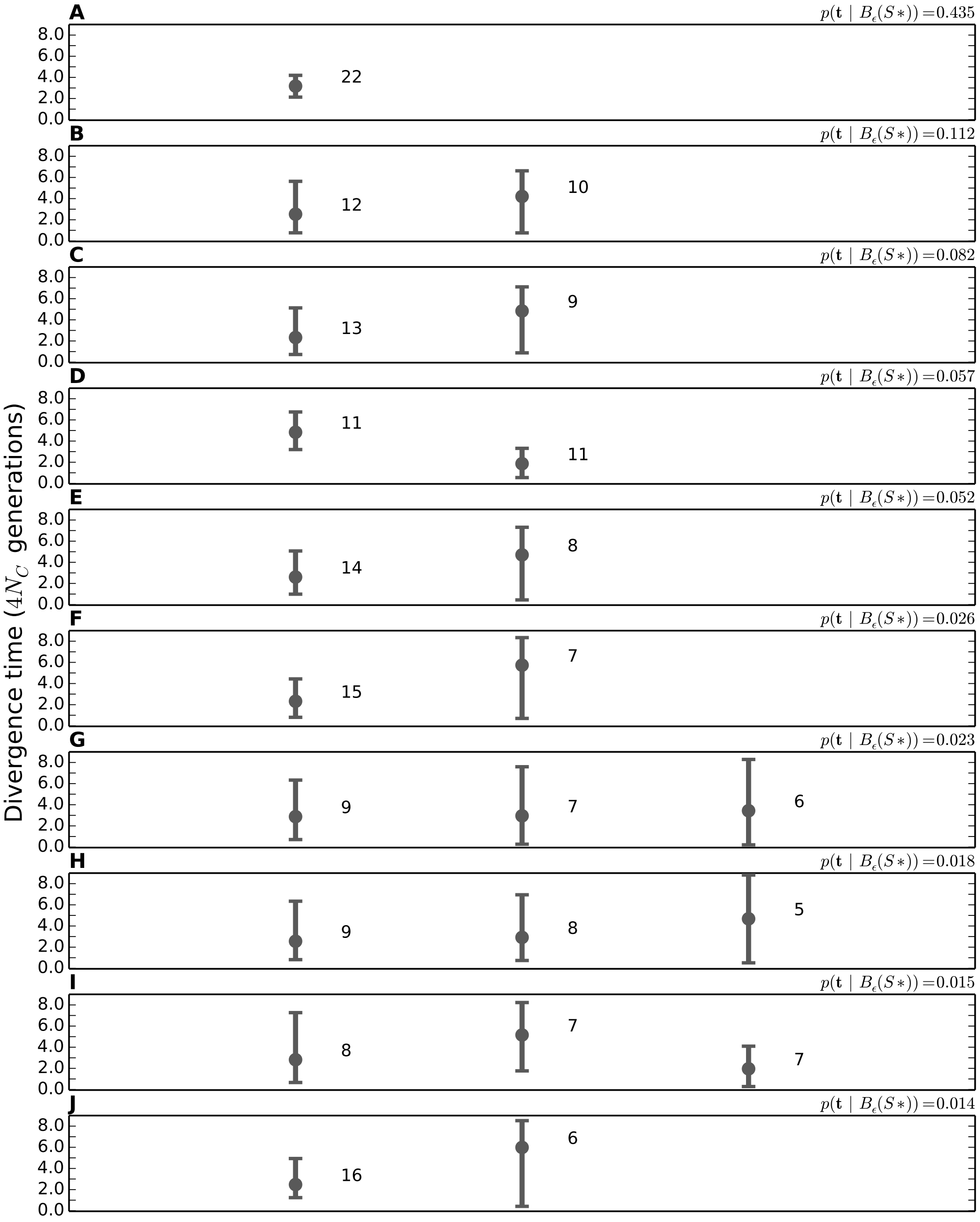

Figure S30: The divergence-model results when the 22 pairs of taxa from the Philippines are analyzed under the $\mathbf{M}_{msBayes}$ model (Table 4). The 10 unordered divergence models with highest posterior probability ($p(\mathbf{t} \mid B_\epsilon(\mathbf{S}^*))$) are shown, where the numbers indicate the inferred number of taxon pairs that diverged at each event. The times indicate the posterior median and 95% highest posterior density (HPD) interval conditional on each divergence model. For each model, times are summarized across posterior samples by the number of taxon pairs associated with each divergence. For models in which there are multiple divergence events with the same number of taxon pairs, the events are sorted by time to summarize the divergence times in a consistant way.

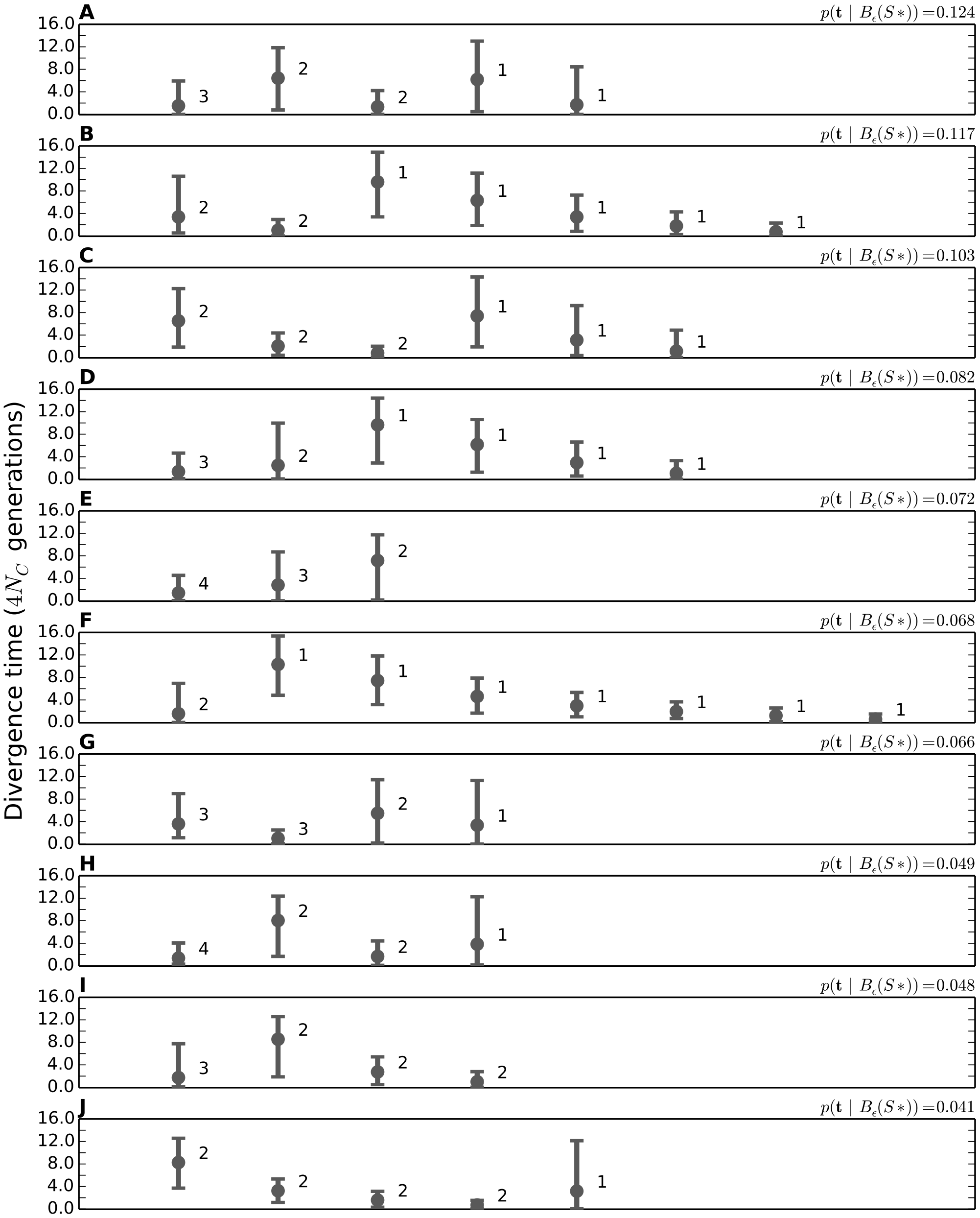

Figure S31: The divergence-model results when the 9 pairs of taxa from the Islands of Negros and Panay are analyzed under the $\mathbb{M}_{DPP}$ model sampling over unordered models of divergence. (Table 4). The 10 unordered divergence models with highest posterior probability ($p(\mathbf{t} \mid B_\epsilon(\mathbf{S}^*))$) are shown, where the numbers indicate the inferred number of taxon pairs that diverged at each event. The times indicate the posterior median and 95% highest posterior density (HPD) interval conditional on each divergence model. For each model, times are summarized across posterior samples by the number of taxon pairs associated with each divergence. For models in which there are multiple divergence events with the same number of taxon pairs, the events are sorted by time to summarize the divergence times in a consistant way.

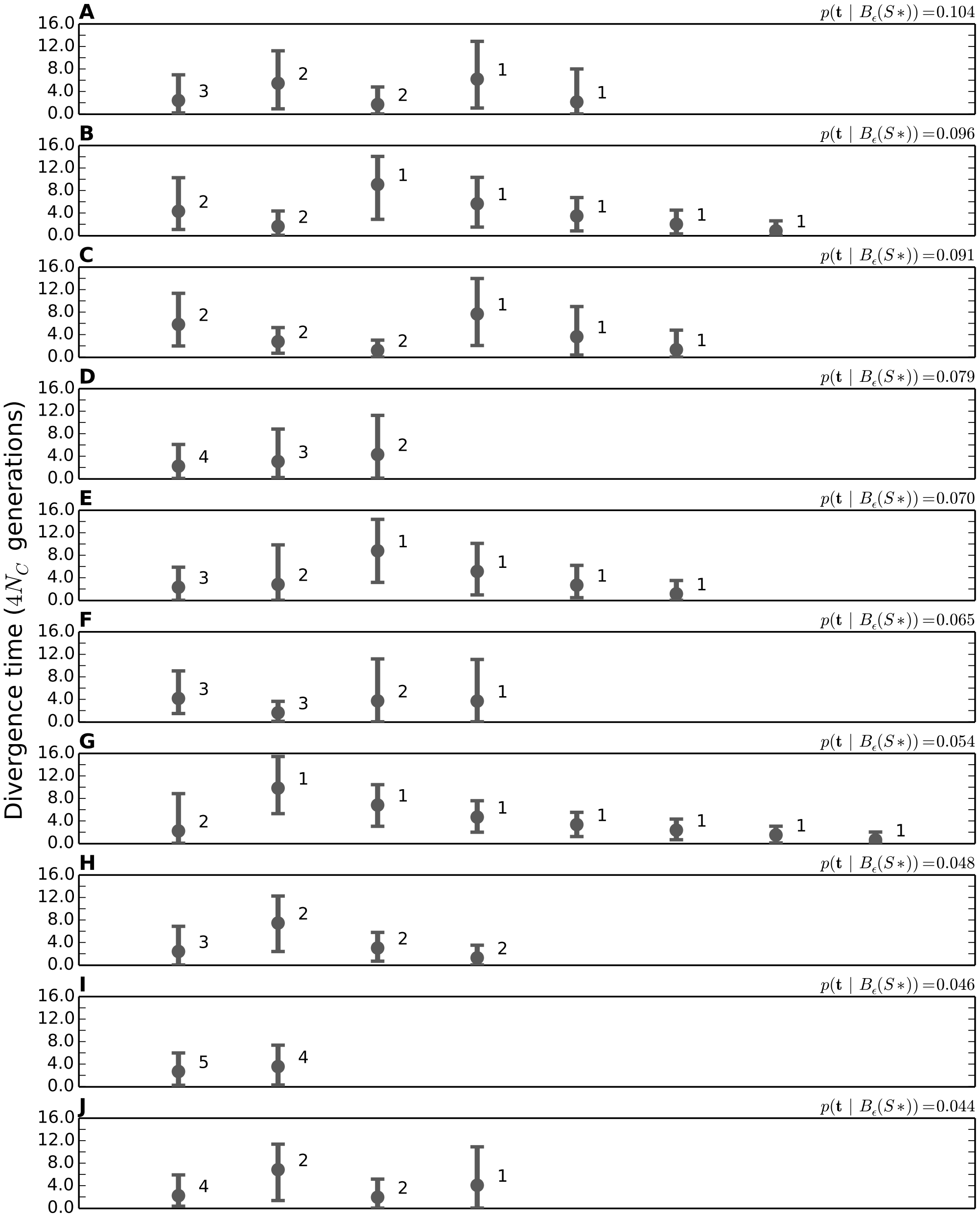

Figure S32: The divergence-model results when the 9 pairs of taxa from the Islands of Negros and Panay are analyzed under the $\mathbb{M}^{\circ}_{DPP}$ model sampling over ordered models of divergence. (Table 4). The posterior sample of divergence models were summarized while ignoring the identity of the taxon pairs in order to compare the results of the $\mathbb{M}_{DPP}$ model. The 10 unordered divergence models with highest posterior probability ($p(\mathbf{t} \mid B_\epsilon(\mathbf{S}^*))$) are shown, where the numbers indicate the inferred number of taxon pairs that diverged at each event. The times indicate the posterior median and 95% highest posterior density (HPD) interval conditional on each divergence model. For each model, times are summarized across posterior samples by the number of taxon pairs associated with each divergence. For models in which there are multiple divergence events with the same number of taxon pairs, the events are sorted by time to summarize the divergence times in a consistant way.

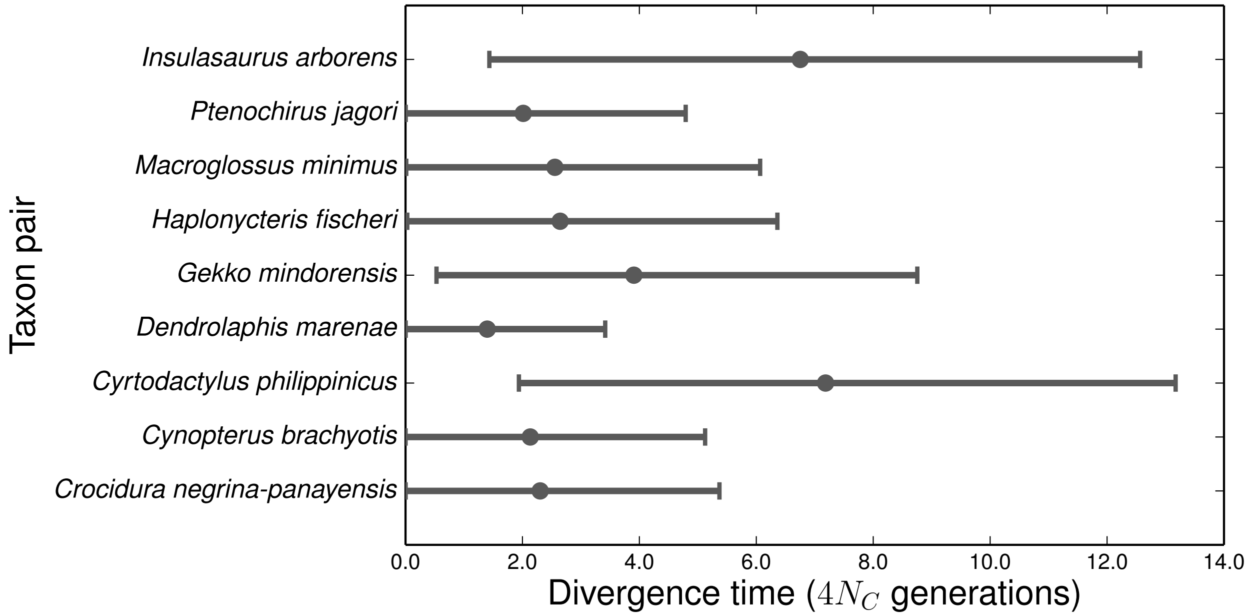

Figure S33: The marginal divergence-time results when the 9 pairs of taxa from the Islands of Negros and Panay are analyzed under the $\mathbb{M}^{\circ}_{DPP}$ model that samples over ordered models of divergence (Table 4). The times indicate the posterior median and 95% highest posterior density (HPD) interval.
